# Human‐specific *ARHGAP11B* ensures human‐like basal progenitor levels in hominid cerebral organoids

**DOI:** 10.15252/embr.202254728

**Published:** 2022-09-13

**Authors:** Jan Fischer, Eduardo Fernández Ortuño, Fabio Marsoner, Annasara Artioli, Jula Peters, Takashi Namba, Christina Eugster Oegema, Wieland B. Huttner, Julia Ladewig, Michael Heide

**Affiliations:** ^1^ Max Planck Institute of Molecular Cell Biology and Genetics Pfotenhauerstrasse 108 Dresden Germany; ^2^ Central Institute of Mental Health University of Heidelberg/Medical Faculty Mannheim Mannheim Germany; ^3^ Hector Institute for Translational Brain Research (HITBR gGmbH) Mannheim Germany; ^4^ German Cancer Research Center (DKFZ) Heidelberg Germany; ^5^ German Primate Center Leibniz Institute for Primate Research Göttingen Germany; ^6^ Present address: Institute for Clinical Genetics University Hospital Carl Gustav Carus Dresden Germany; ^7^ Present address: Neuroscience Center, HiLIFE ‐ Helsinki Institute of Life Science University of Helsinki Helsinki Finland

**Keywords:** *ARHGAP11B*, brain organoids, human‐specific genes, neocortex development, neocortex evolution, Evolution & Ecology, Neuroscience

## Abstract

The human‐specific gene *ARHGAP11B* has been implicated in human neocortex expansion. However, the extent of *ARHGAP11B*'s contribution to this expansion during hominid evolution is unknown. Here we address this issue by genetic manipulation of ARHGAP11B levels and function in chimpanzee and human cerebral organoids. *ARHGAP11B* expression in chimpanzee cerebral organoids doubles basal progenitor levels, the class of cortical progenitors with a key role in neocortex expansion. Conversely, interference with ARHGAP11B's function in human cerebral organoids decreases basal progenitors down to the chimpanzee level. Moreover, *ARHGAP11A* or *ARHGAP11B* rescue experiments in *ARHGAP11A* plus *ARHGAP11B* double‐knockout human forebrain organoids indicate that lack of ARHGAP11B, but not of ARHGAP11A, decreases the abundance of basal radial glia—the basal progenitor type thought to be of particular relevance for neocortex expansion. Taken together, our findings demonstrate that *ARHGAP11B* is necessary and sufficient to ensure the elevated basal progenitor levels that characterize the fetal human neocortex, suggesting that this human‐specific gene was a major contributor to neocortex expansion during human evolution.

## Graphical abstract

Human and chimpanzee cerebral organoids reveal that human‐specific *ARHGAP11B* is necessary and sufficient to ensure the elevated basal progenitor levels that characterize the fetal human neocortex.

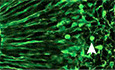

## Introduction

The neocortex, the evolutionarily youngest part of the brain, is the seat of our higher cognitive abilities. It is therefore of crucial importance to investigate the development of the neocortex. This has been done in several model systems and has provided pivotal insight (Rakic, 2009; Lui *et al*, 2011; Florio & Huttner, 2014; Sun & Hevner, 2014; Dehay *et al*, 2015; Molnar *et al*, 2019; Silver *et al*, 2019). Identifying the features that characterize the development specifically of the human neocortex is, however, a fundamental challenge. The human neocortex exhibits an increase in size and in the numbers of neurons compared with non‐human primates. This increase is thought to reflect a greater proliferative capacity of the cortical stem and progenitor cells (collectively referred to as cortical neural progenitor cells (cNPCs)) in human (Fish *et al*, 2008; Lui *et al*, 2011; Florio & Huttner, 2014; Sun & Hevner, 2014; Dehay *et al*, 2015).

Over the past 8 years, genes have been identified that specifically evolved in the human lineage, that are preferentially expressed in cNPCs, and that promote cNPC proliferation (Florio *et al*, 2015, 2018; Fiddes *et al*, 2018; Suzuki *et al*, 2018). Such genes have therefore been implicated in human‐specific features of neocortical development (Florio *et al*, 2015, 2018; Fiddes *et al*, 2018; Suzuki *et al*, 2018; Heide & Huttner, 2021). However, a human–chimpanzee comparison to explore whether such human‐specific genes are responsible for a human‐like cNPC proliferative capacity has not yet been carried out, mainly for the following reason. Whereas tissue of developing human neocortex can, in principle, be obtained and subjected to experimental studies, this is not the case for tissue of developing chimpanzee neocortex.

A way out of this dilemma has been provided by recent, seminal advances in pluripotent stem cell (PSC) research, which led to the development of the brain organoid technology (Watanabe *et al*, 2005; Eiraku *et al*, 2008; Kadoshima *et al*, 2013; Lancaster *et al*, 2013, 2017; Pasca *et al*, 2015; Qian *et al*, 2016; Quadrato *et al*, 2017; Karzbrun *et al*, 2018; Giandomenico *et al*, 2019). A specific subtype of brain organoids, the cerebral organoids are relatively small (a few mm in diameter), three‐dimensional (3D) structured cell assemblies that can be grown from embryonic stem cells (ESCs) (in the case of human) or induced pluripotent stem cells (iPSCs) (in the case of human and chimpanzee) and that emulate cerebral tissue (Lancaster *et al*, 2013, 2017; Kelava & Lancaster, 2016; Di Lullo & Kriegstein, 2017; Arlotta, 2018; Heide *et al*, 2018; Fischer *et al*, 2019).

Thus, cerebral organoids have been shown to exhibit several (albeit not all) hallmarks of developing neocortical tissue, including a ventricular zone (VZ) and subventricular zone (SVZ) as well as the two major classes of cNPCs therein, the apical progenitors (APs) and the basal progenitors (BPs) (Kadoshima *et al*, 2013; Lancaster *et al*, 2013; Qian *et al*, 2016; Quadrato *et al*, 2017; Heide *et al*, 2018). Cerebral organoids also exhibit a cortical plate‐like region with neuronal layers (NLs) containing the various types of cortical neurons (Kadoshima *et al*, 2013; Lancaster *et al*, 2013, 2017; Qian *et al*, 2016; Quadrato *et al*, 2017; Heide *et al*, 2018; Velasco *et al*, 2019). Moreover, human cerebral organoids have been shown to recapitulate gene expression programs of fetal human neocortex development (Camp *et al*, 2015; Velasco *et al*, 2019; Bhaduri *et al*, 2020).

In light of these findings, cerebral organoids have emerged as a promising primate model system to study cortical development and evolution. In addition, cerebral organoids offer the opportunity of extrinsic genetic manipulation (Fischer *et al*, 2019). This is particularly relevant in the case of human‐specific genes that in fetal human neocortex are preferentially expressed in cNPCs and hence have been implicated in human‐specific features of neocortical development (Florio *et al*, 2015, 2018; Fiddes *et al*, 2018; Suzuki *et al*, 2018; Heide & Huttner, 2021). Examining such genes for their function in, and effects on, cNPC proliferation in cerebral organoids of human and chimpanzee, respectively, could not only provide corroborating evidence in support of their presumptive role in neocortical development during human evolution, but also provide further insights into their action and effects.

*ARHGAP11B* is a human‐specific gene (Sudmant *et al*, 2010; Dennis *et al*, 2017) and the first such gene to have been implicated in human neocortical development and evolution (Florio *et al*, 2015, 2016; Kalebic *et al*, 2018; Heide *et al*, 2020; Xing *et al*, 2021). In fetal human neocortex, *ARHGAP11B* is preferentially expressed in cNPCs (Florio *et al*, 2015, 2018). When (over)expressed in embryonic mouse and ferret neocortex, *ARHGAP11B* has been found to increase the proliferation and abundance of BPs (Florio *et al*, 2015; Kalebic *et al*, 2018; Xing *et al*, 2021), the cNPC class implicated in neocortical expansion during human development and evolution (Lui *et al*, 2011; Borrell & Götz, 2014; Florio & Huttner, 2014; Dehay *et al*, 2015). Moreover, a recent study in which *ARHGAP11B* was expressed under the control of its own promoter to physiological levels in the fetal neocortex of the common marmoset has demonstrated that this human‐specific gene can indeed induce the hallmarks of neocortical expansion in this non‐human primate, increasing neocortex size, folding, BP levels, and upper‐layer neuron numbers (Heide *et al*, 2020). Consistent with this, physiological *ARHGAP11B* expression in a transgenic mouse line not only resulted in increased neocortical size and upper‐layer neuron numbers that persist into adulthood, but also in increased cognitive abilities (Xing *et al*, 2021). Importantly, the ability of *ARHGAP11B* to increase the proliferation and abundance of BPs has been attributed not to the gene as it arose ≈ 5 mya by partial duplication of the widespread gene *ARHGAP11A* (Sudmant *et al*, 2010; Dennis *et al*, 2017), referred to as ancestral *ARHGAP11B*, but to an *ARHGAP11B* gene that subsequently underwent a point mutation, referred to as modern *ARHGAP11B* (Florio *et al*, 2016). These studies therefore establish (i) that modern *ARHGAP11B* is sufficient to expand BPs, including in primates, and (ii) that the resulting neocortex expansion and increase in upper‐layer neuron numbers are associated with an increase in cognitive abilities.

Considering these sets of findings together, the question arises to which extent *ARHGAP11B* contributes to the increase in cycling BPs in the context of the expansion of the neocortex in the course of human evolution. A first clue in this regard was obtained by the observation that a truncated form of the ARHGAP11A protein, ARHGAP11A220, which acts in a dominant‐negative manner on ARHGAP11B's ability to amplify BPs in embryonic mouse neocortex, reduces the abundance of cycling BPs in fetal human neocortical tissue *ex vivo* (Namba *et al*, 2020).

Yet, a key question regarding *ARHGAP11B*'s role in human neocortex expansion remains unanswered: Can the human‐specific *ARHGAP11B* gene increase the proliferation and abundance of BPs when expressed in cerebral organoids of the chimpanzee, our closest living relative? And, conversely, regarding human neocortical development: Is *ARHGAP11B* required to maintain the full level of BP proliferation and abundance in human cerebral organoids?

In the present study, we have addressed these questions. In doing so, we provide support for the notion that *ARHGAP11B* is sufficient to increase BP proliferation and abundance to a human‐like level in chimpanzee cerebral organoids. Conversely, we find that dominant‐negative inhibition of ARHGAP11B's function by ARHGAP11A220 reduces cycling BP abundance in human cerebral organoids to the chimpanzee level. Finally, by subjecting *ARHGAP11A* plus *ARHGAP11B* double‐knockout human forebrain organoids to either *ARHGAP11A* or *ARHGAP11B* rescue, we find that *ARHGAP11B* is essential to maintain the level of basal (or outer) radial glia (bRG), the BP type of particular relevance for neocortex expansion. Together, these findings provide direct evidence in support of an indispensable role of *ARHGAP11B* in neocortical expansion during human development and evolution.

## Results

### Human and chimpanzee cerebral organoids as a test system for gene function

**Figure 1:**
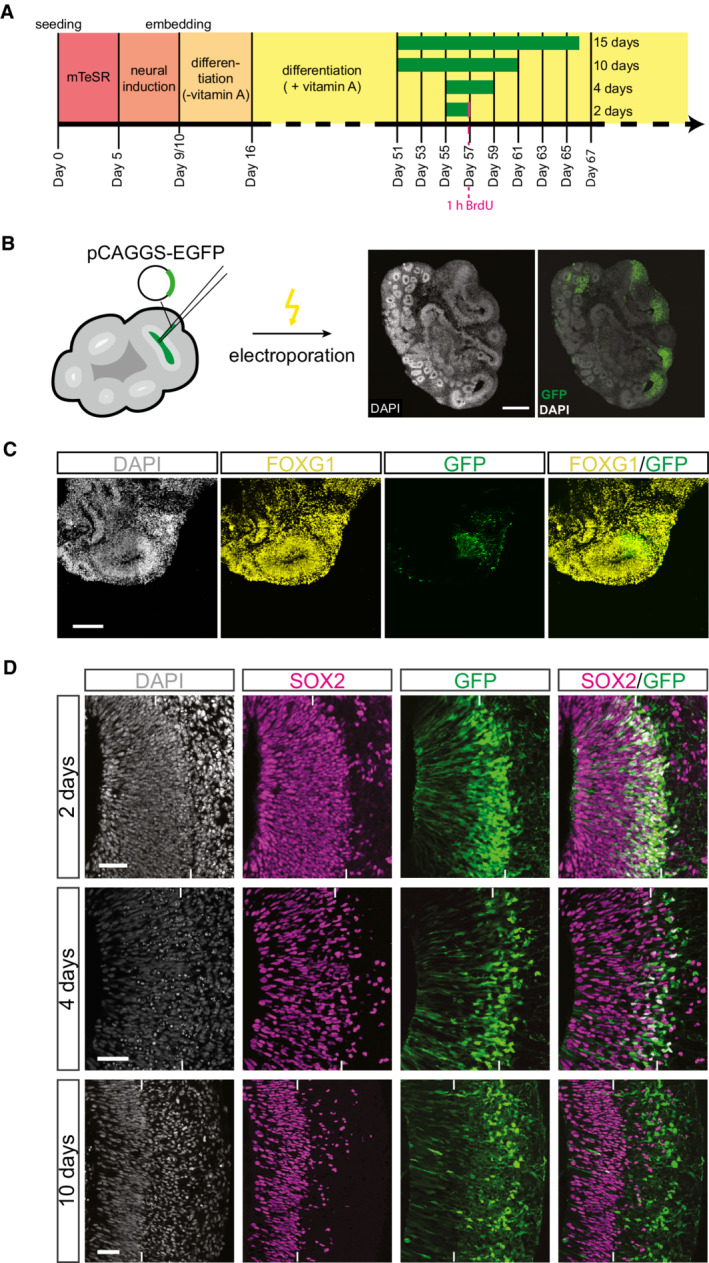
Experimental protocol of cerebral organoid production and time points of electroporation and analyses Timeline of cerebral organoid production detailing media as well as time points of electroporation (beginning of green bars), duration of vector expression (lengths of green bars; 2, 4, 10 and 15 days), and time points of fixation and analysis (end of green bars) of cerebral organoids.Left: Cartoon depicting the microinjection and electroporation of a ventricle‐like structure of a cerebral organoid; Right: Immunofluorescence for GFP (green), combined with DAPI staining (white), of a 57‐day‐old chimpanzee cerebral organoid 2 days after electroporation with GFP expression plasmid plus control plasmid. Scale bar, 500 μm.Double immunofluorescence for GFP (green) and the telencephalic marker FOXG1 (yellow), combined with DAPI staining (white), of a 57‐day‐old chimpanzee cerebral organoid 2 days after electroporation with GFP expression plasmid plus control plasmid. Scale bar, 150 μm.Double immunofluorescence for SOX2 (magenta) and GFP (green), combined with DAPI staining (white), of a 57‐day‐old chimpanzee cerebral organoid 2 days after electroporation with GFP expression plasmid plus control plasmid (first row), of a 59‐day‐old chimpanzee cerebral organoid 4 days after electroporation with GFP expression plasmid plus control plasmid (second row), and a 61‐day‐old chimpanzee cerebral organoid 10 days after electroporation with GFP expression plasmid plus control plasmid (third row). Tick marks indicate the border between VZ and SVZ/NL. Scale bars, 50 μm.

For most of the data presented in this study, human and chimpanzee cerebral organoids were grown from human iPSCs of the line SC102A1 (Camp *et al*, 2015; Mora‐Bermudez *et al*, 2016; Kanton *et al*, 2019) and chimpanzee iPSCs of the line Sandra A (Mora‐Bermudez *et al*, 2016; Kanton *et al*, 2019), respectively (for the iPSC line used to generate knockout forebrain organoids, see below). Cerebral organoids were generated according to an established protocol (Lancaster *et al*, 2013; Lancaster & Knoblich, 2014; Camp *et al*, 2015; Mora‐Bermudez *et al*, 2016; Kanton *et al*, 2019). In brief, iPSCs were aggregated to form embryoid bodies followed by their transformation into 3D cerebral tissue exhibiting numerous ventricular structures (Fig 1A). Cerebral organoids were subjected to manipulations (see below) between day 51 and 55 (see Fig 1A). This time window was chosen based on the known time courses of VZ formation, SVZ formation, and deep‐ and upper‐layer neuron generation. In contrast to macaque organoids, these time courses are roughly comparable between human and chimpanzee cerebral organoids (Mora‐Bermudez *et al*, 2016; Otani *et al*, 2016; Kanton *et al*, 2019). After 51 or 55 days of organoid culture, various mixtures of DNA constructs, consisting of a cytosolic‐GFP expression vector and either an expression vector with the cDNA of interest or the corresponding control vector, were then microinjected into the lumen of the larger ventricle‐like structures within the cerebral organoids, followed by electroporation to transfect the cNPCs in the VZ (Fig 1A and B; Lancaster *et al*, 2013; Li *et al*, 2017; Fischer *et al*, 2019; Giandomenico *et al*, 2019). Depending on the specific scientific question asked, organoid culture was continued for 2–15 days after electroporation followed by fixation of the cerebral organoids, in the case of 2 days with addition of BrdU 1 h prior to fixation as indicated (Fig 1A). Fixed cerebral organoids were subjected to immunohistochemical analyses, using GFP immunofluorescence to identify the targeted cNPCs and their progeny (Fig 1A and B). These organoids were mainly of telencephalic identity as indicated by the expression of the telencephalic marker FOXG1 (Fig 1C).

Representative examples of control vector‐transfected chimpanzee cerebral organoids 2, 4, and 10 days after electroporation are presented in Fig 1D and Appendix Figs S1–S3. These images show that depending on the time after electroporation, different cell populations in distinct zones of the developing cerebral organoid wall contain GFP‐positive cells. This GFP expression reveals the transfected APs and their progeny and hence indicates the cells that, either directly or by inheritance, would be affected by a given electroporated DNA. Two days after electroporation, the majority of the GFP‐positive cells was still observed in the VZ, colocalizing with a marker of proliferating cNPCs, SOX2 (Fig 1D). A minority of the GFP‐positive cells was already observed basal to the VZ in the SVZ and neuronal layers (NL), colocalizing with a marker of basal progenitors, TBR2 (Englund *et al*, 2005; Sessa *et al*, 2008; Appendix Fig S1), but barely colocalizing with a marker of deep‐layer neurons, CTIP2 (Arlotta *et al*, 2005; Molyneaux *et al*, 2007; Appendix Fig S2), and not colocalizing with a marker of upper‐layer neurons, SATB2 (Alcamo *et al*, 2008; Britanova *et al*, 2008; Appendix Fig S3). These data are consistent with the length of the cell cycle of APs observed in chimpanzee cerebral organoids of ≈ 2 days (Mora‐Bermudez *et al*, 2016) and suggest that the GFP‐positive cells observed in the VZ 2 days after electroporation were either targeted APs, daughter APs of targeted APs, newborn BPs derived from targeted APs, or (few) newborn deep‐layer neurons derived from targeted progenitors.

Four days after electroporation, GFP‐positive cells were observed in the basal region of the VZ, at the boundary between VZ and SVZ, and in the SVZ and NL, largely colocalizing with either SOX2 (Fig 1D), TBR2 (Appendix Fig S1) or CTIP2 (Appendix Fig S2) but not with SATB2 (Appendix Fig S3). This suggests that the GFP‐positive cells observed in the VZ 4 days after electroporation were daughter APs of targeted APs, BPs derived from targeted APs, or newborn deep‐layer neurons derived from targeted progenitors.

Ten days after electroporation, GFP‐positive cells were observed mostly in the basal SVZ and NL, colocalizing rarely with SOX2 (Fig 1D), still somewhat with TBR2 (Appendix Fig S1), mostly with CTIP2 (Appendix Fig S2), but not yet often with SATB2 (Appendix Fig S3). This is consistent with the notion that this longer period after electroporation should allow the targeted APs to carry out multiple rounds of BP‐generating cell divisions, with the resulting BPs carrying out neuron‐generating divisions. Accordingly, after the 10‐day period following electroporation, a greater proportion of the targeted AP progeny is neurons, mostly of the deep‐layer type, than after the 4‐day period following electroporation (Appendix Figs S2 and S3).

### Expression of human‐specific *ARHGAP11B* in chimpanzee cerebral organoids increases the abundance of cycling BPs

**Figure 2:**
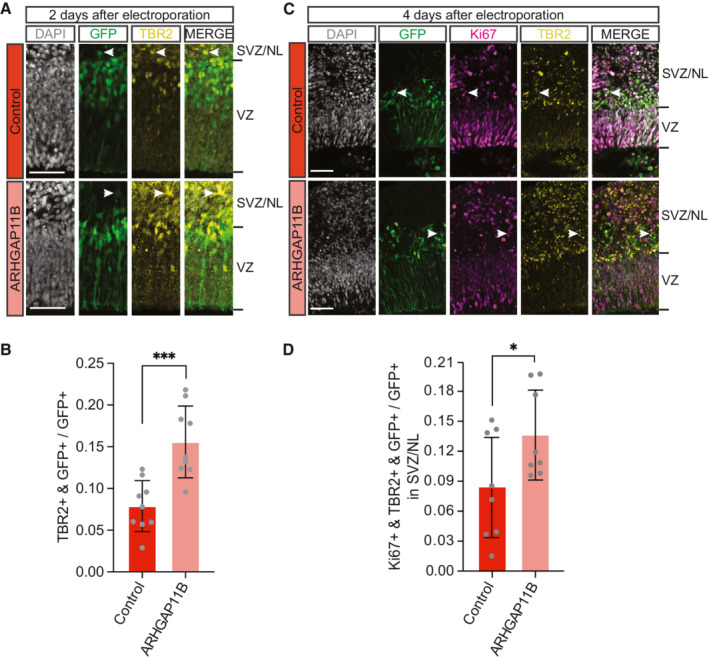
Expression of *ARHGAP11B* in chimpanzee cerebral organoids increases the abundance of cycling BPs Double immunofluorescence for GFP (green) and the BP marker TBR2 (yellow), combined with DAPI staining (white), of a 57‐day‐old chimpanzee cerebral organoid 2 days after electroporation with GFP expression plasmid plus either control plasmid (top) or *ARHGAP11B* expression plasmid (bottom). Tick marks indicate the borders of the VZ and SVZ/NL; arrowheads indicate examples of GFP+ and TBR2+ double‐positive cells. Scale bars, 50 μm.Quantification of the proportion of GFP+ cells that are TBR2+ in 57‐day‐old chimpanzee cerebral organoids 2 days after electroporation with GFP expression plasmid plus either control plasmid (dark red) or *ARHGAP11B* expression plasmid (light red). Data are the mean of nine control and nine *ARHGAP11B*‐transfected cerebral organoids of two independent batches each; error bars indicate SD; ***P < 0.001 (two‐sided Student's *t*‐test).Triple immunofluorescence for GFP (green), the cycling cell marker Ki67 (magenta), and TBR2 (yellow), combined with DAPI staining (white), of a 59‐day‐old chimpanzee cerebral organoid 4 days after electroporation with GFP expression plasmid plus either control plasmid (top) or *ARHGAP11B* expression plasmid (bottom). Tick marks indicate the borders of the VZ and SVZ/NL; arrowheads indicate examples of GFP+, Ki67+ and TBR2+ triple‐positive cells. Scale bars, 50 μm.Quantification of the proportion of GFP+ cells in the SVZ/NL that are Ki67+ and TBR2+ double‐positive in 59‐day‐old chimpanzee cerebral organoids 4 days after electroporation with GFP expression plasmid plus either control plasmid (dark red) or *ARHGAP11B* expression plasmid (light red). Data are the mean of eight control and eight *ARHGAP11B*‐transfected cerebral organoids of four independent batches each; error bars indicate SD; *P < 0.05 (one‐sided Wilcoxon rank sum test).

**Figure EV1:**
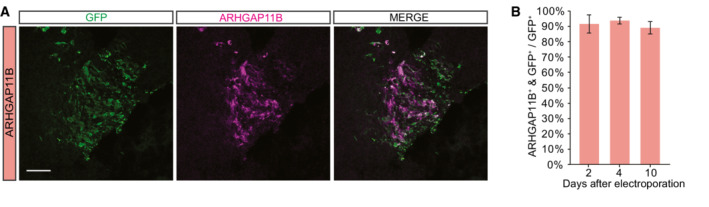
GFP and *ARHGAP11B* are co‐expressed when co‐electroporated in chimpanzee cerebral organoids Double immunofluorescence for GFP (green) and ARHGAP11B (magenta) of a 59‐day‐old chimpanzee cerebral organoid 4 days after electroporation with GFP expression plasmid plus *ARHGAP11B* expression plasmid. Note that GFP and ARHGAP11B immunofluorescence signals do not completely overlap, as GFP is localized in the cytoplasm, whereas ARHGAP11B is localized in mitochondria. Scale bar, 50 μm.Quantification of the percentage of GFP+ cells that are ARHGAP11B+ in 57‐, 59‐, and 61‐day‐old chimpanzee cerebral organoids 2, 4, and 10 days after electroporation with GFP expression plasmid plus *ARHGAP11B* expression plasmid. Data are the mean of five, six, or seven ventricle‐like structures 2, 4, or 10 days after electroporation of three *ARHGAP11B*‐transfected cerebral organoids each; error bars indicate SD.

Having established transfection of cerebral organoids as our test system, we first investigated whether, similar to the other non‐human model systems of neocortex development previously used to study the effects of *ARHGAP11B* (Florio *et al*, 2015, 2016; Kalebic *et al*, 2018; Heide *et al*, 2020; Xing *et al*, 2021), *ARHGAP11B* would increase BP proliferation and abundance when expressed in chimpanzee cerebral organoids. For this purpose, we employed a previously used construct leading to ectopic expression of *ARHGAP11B* under the constitutive CAGGS promoter (pCAGGS‐*ARHGAP11B*; Florio *et al*, 2015). (For details of this overexpression construct, and the justification and appropriateness of its use, please see Materials and Methods). We used the experimental protocol described above and in Fig 1 to determine the co‐electroporation efficiency of the pCAGGS‐*EGFP* and the pCAGGs‐*ARHGAP11B* vectors in chimpanzee cerebral organoids. We found that ≥ 90% of the GFP‐positive progeny of the targeted APs was also positive for ARHGAP11B by immunofluorescence at 2, 4, and 10 days after electroporation (Fig EV1A and B), indicating a high electroporation efficiency. Hence, we used this experimental protocol with a 2‐day period between the electroporation of chimpanzee cerebral organoids with the *ARHGAP11B* expression vector and analysis of the transfected organoids by immunofluorescence for the BP marker TBR2. We found a marked, twofold increase in the proportion of the GFP‐positive progeny of the targeted APs that were TBR2‐positive, and hence newborn BPs, in the *ARHGAP11B*‐transfected chimpanzee organoids in comparison to control‐transfected organoids (Fig 2A and B). This increase by ≈ 10% points of the total GFP+ cells likely occurred at the expense of the APs, as *ARHGAP11B* has previously been shown to induce symmetric, consumptive BP‐genic divisions of these cNPCs. These data therefore indicate that *ARHGAP11B* increases the abundance of BPs upon expression in chimpanzee cerebral organoids.

To further analyze the effect of *ARHGAP11B* on BP abundance in chimpanzee cerebral organoids, we used the same experimental protocol as described above and in Fig 1, but this time with a 4‐day period between electroporation and analysis. Compared with control, transfection of the chimpanzee cerebral organoids with *ARHGAP11B* caused, again, an increase in the proportion of the GFP‐positive progeny of the targeted APs in the SVZ and NL that were Ki67+ and TBR2+ double‐positive, that is, cycling BPs (Fig 2C and D). In this case, the increase in Ki67+ TBR2+ cells most likely occurred at the expense of newborn neurons in the SVZ and NL (see below).

Taken together, these results indicate that, similar to results previously obtained in embryonic mouse (Florio *et al*, 2015; Xing *et al*, 2021), embryonic ferret (Kalebic *et al*, 2018), and fetal marmoset (Heide *et al*, 2020) neocortex, the human‐specific gene *ARHGAP11B* can substantially increase the abundance of cycling BPs in developing cerebral cortex‐like tissue of our closest living relative, the chimpanzee.

### *ARHGAP11B* expression in chimpanzee cerebral organoids increases the abundance of bRG

**Figure 3:**
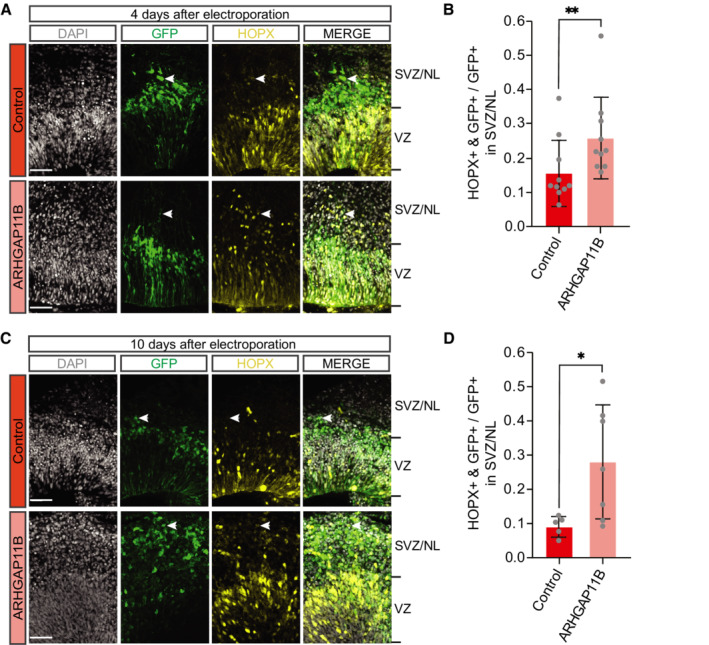
Expression of *ARHGAP11B* in chimpanzee cerebral organoids increases the abundance of HOPX‐positive cells in the SVZ Double immunofluorescence for GFP (green) and the radial glia marker HOPX (yellow), combined with DAPI staining (white), of a 59‐day‐old chimpanzee cerebral organoid 4 days after electroporation with GFP expression plasmid plus either control plasmid (top) or *ARHGAP11B* expression plasmid (bottom). Tick marks indicate the borders of the VZ and SVZ/NL; arrowheads indicate examples of GFP+ and HOPX+ double‐positive cells. Scale bars, 50 μm.Quantification of the proportion of GFP+ cells in the SVZ/NL that are HOPX+ in 59‐day‐old chimpanzee cerebral organoids 4 days after electroporation with GFP expression plasmid plus either control plasmid (dark red) or *ARHGAP11B* expression plasmid (light red). Data are the mean of 10 control and 10 *ARHGAP11B*‐transfected cerebral organoids of three independent batches each; error bars indicate SD; **P < 0.01 (one‐sided Wilcoxon rank sum test).Double immunofluorescence for GFP (green) and HOPX (yellow), combined with DAPI staining (white), of a 61‐day‐old chimpanzee cerebral organoid 10 days after electroporation with GFP expression plasmid plus either control plasmid (top) or *ARHGAP11B* expression plasmid (bottom). Tick marks indicate the borders of the VZ and SVZ/NL; arrowheads indicate examples of GFP+ and HOPX+ double‐positive cells. Scale bars, 50 μm.Quantification of the proportion of GFP+ cells in the SVZ/NL that are HOPX+ in 61‐day‐old chimpanzee cerebral organoids 10 days after electroporation with GFP expression plasmid plus either control plasmid (dark red) or *ARHGAP11B* expression plasmid (light red). Data are the mean of five control and seven *ARHGAP11B*‐transfected cerebral organoids of three independent batches each; error bars indicate SD; *P < 0.05 (two‐sided Student's *t*‐test).

In principle, two different types of BPs can be distinguished, i.e., basal intermediate progenitors (bIPs) and basal radial glia (bRG, also called outer radial glia; Haubensak *et al*, 2004; Miyata *et al*, 2004; Noctor *et al*, 2004; Fietz *et al*, 2010; Hansen *et al*, 2010; Reillo *et al*, 2011; Betizeau *et al*, 2013). bRG, in particular, are thought to be key for mammalian neocortex evolution and to drive expansion and folding of the human neocortex (Lui *et al*, 2011; Borrell & Götz, 2014; Florio & Huttner, 2014; Dehay *et al*, 2015; Fernandez *et al*, 2016; Llinares‐Benadero & Borrell, 2019). In light of the increase in BP abundance upon *ARHGAP11B* expression in chimpanzee cerebral organoids (Fig 2), we next asked whether this increase applied to bRG. For this purpose, we used immunofluorescence for HOPX, a marker of radial glia (Pollen *et al*, 2015; Vaid *et al*, 2018), and quantified, specifically in regions basal to the VZ, i.e., in the SVZ and NL, the proportion of the GFP‐positive progeny of the targeted APs that were HOPX‐positive, which would be indicative of bRG. Four days after electroporation, we found almost a doubling in this proportion in the *ARHGAP11B*‐transfected chimpanzee cerebral organoids in comparison to control‐transfected organoids (Fig 3A and B). This increase by ≈ 10% points presumably occurred, again, at the expense of newborn neurons in the SVZ and NL (see below).

**Figure EV2:**
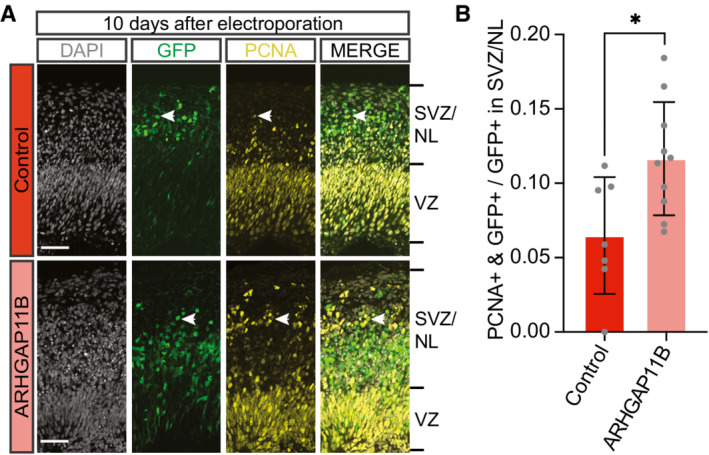
Expression of *ARHGAP11B* in chimpanzee cerebral organoids increases the abundance of PCNA‐positive cells in the SVZ Double immunofluorescence for GFP (green) and the cycling cell marker PCNA (yellow), combined with DAPI staining (white), of a 61‐day‐old chimpanzee cerebral organoids 10 days after electroporation with GFP expression plasmid plus either control plasmid (top) or *ARHGAP11B* expression plasmid (bottom). Tick marks indicate the borders of the VZ and SVZ/NL; arrowheads indicate examples of GFP+ and PCNA+ double‐positive cells. Note that the same electroporated regions are depicted in Fig 4 with a different marker (Hu). Scale bars, 50 μm.Quantification of the proportion of GFP+ cells in the SVZ/NL that are PCNA+ in 61‐day‐old chimpanzee cerebral organoids 10 days after electroporation with GFP expression plasmid plus either control plasmid (dark red) or *ARHGAP11B* expression plasmid (light red). Data are the mean of seven control and 10 *ARHGAP11B*‐transfected cerebral organoids of two independent batches each; error bars indicate SD; *P < 0.05 (two‐sided Student's *t*‐test). Source data are available online for this figure.

To corroborate this result, we used a 10‐day period between electroporation and analysis to allow a larger proportion of the GFP‐positive progeny to migrate to the SVZ and NL (see Fig 1A and D, and Appendix Figs S1–S3). We first analyzed the transfected chimpanzee cerebral organoids by PCNA immunofluorescence to quantify cycling cells in the SVZ. Compared with control, transfection of the chimpanzee cerebral organoids with *ARHGAP11B* caused a doubling in the proportion of the GFP‐positive progeny of the targeted APs in the SVZ that were PCNA‐positive (Fig EV2A and B), consistent with a doubling of cycling BPs also after this longer post‐electroporation period. We next performed HOPX immunofluorescence and found a marked, threefold increase in the proportion of the GFP‐positive progeny of the targeted APs in the SVZ that were HOPX‐positive in the *ARHGAP11B*‐transfected chimpanzee cerebral organoids in comparison to control‐transfected organoids (Fig 3C and D).

**Figure 4:**
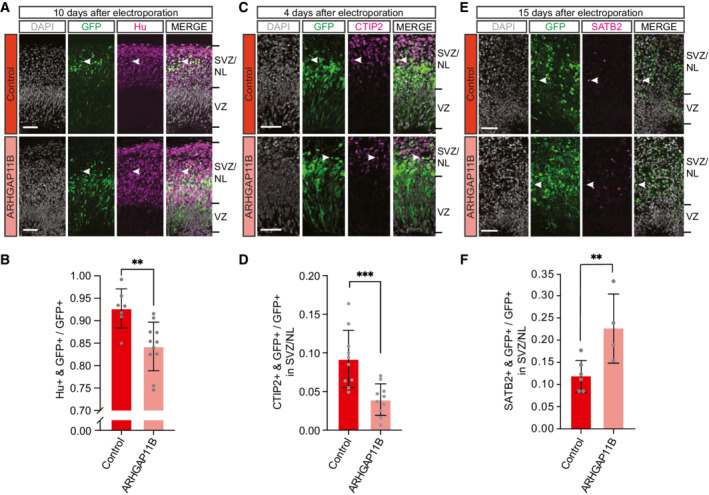
Expression of *ARHGAP11B* in chimpanzee cerebral organoids differentially affects the generation of deep‐layer vs. upper‐layer cortical neurons Double immunofluorescence for GFP (green) and the neuron marker Hu (magenta), combined with DAPI staining (white), of a 61‐day‐old chimpanzee cerebral organoid 10 days after electroporation with GFP expression plasmid plus either control plasmid (top) or *ARHGAP11B* expression plasmid (bottom). Tick marks indicate the borders of the VZ and SVZ/NL; arrowheads indicate examples of GFP+ and Hu+ double‐positive cells. Note that the same electroporated regions are depicted in Fig EV2 with a different marker (PCNA). Scale bars, 50 μm.Quantification of the proportion of GFP+ cells that are Hu+ in 61‐day‐old chimpanzee cerebral organoids 10 days after electroporation with GFP expression plasmid plus either control plasmid (dark red) or *ARHGAP11B* expression plasmid (light red). Data are the mean of seven control and 11 *ARHGAP11B*‐transfected cerebral organoids of two independent batches each; error bars indicate SD; **P < 0.01 (two‐sided Student's *t*‐test).Double immunofluorescence for GFP (green) and the deep‐layer neuron marker CTIP2 (magenta), combined with DAPI staining (white), of a 59‐day‐old chimpanzee cerebral organoid 4 days after electroporation with GFP expression plasmid plus either control plasmid (top) or *ARHGAP11B* expression plasmid (bottom). Tick marks indicate the borders of the VZ and SVZ/NL; arrowheads indicate examples of GFP+ and CTIP2+ double‐positive cells. Scale bars, 50 μm.Quantification of the proportion of GFP+ cells in the SVZ/NL that are CTIP2+ in 59‐day‐old chimpanzee cerebral organoids 4 days after electroporation with GFP expression plasmid plus either control plasmid (dark red) or *ARHGAP11B* expression plasmid (light red). Data are the mean of 10 control and 10 *ARHGAP11B*‐transfected cerebral organoids of two independent batches each; error bars indicate SD; ***P < 0.001 (two‐sided Student's *t*‐test).Double immunofluorescence for GFP (green) and the upper‐layer neuron marker SATB2 (magenta), combined with DAPI staining (white), of a 66‐day‐old chimpanzee cerebral organoid 15 days after electroporation with GFP expression plasmid plus either control plasmid (top) or *ARHGAP11B* expression plasmid (bottom). Tick marks indicate the borders of the VZ and SVZ/NL; arrowheads indicate examples of GFP+ and SATB2+ double‐positive cells. Scale bars, 50 μm.Quantification of the proportion of GFP+ cells in the SVZ/NL that are SATB2+ in 66‐day‐old chimpanzee cerebral organoids 15 days after electroporation with GFP expression plasmid plus either control plasmid (dark red) or *ARHGAP11B* expression plasmid (light red). Data are the mean of six control and four *ARHGAP11B*‐transfected cerebral organoids of two independent batches each; error bars indicate SD; **P < 0.01 (one‐sided Wilcoxon rank sum test). Source data are available online for this figure.

In summary, we conclude that the increase in cycling BP abundance upon expression of the human‐specific gene *ARHGAP11B* in chimpanzee cerebral organoids includes—similar to results obtained in animal model systems (Florio *et al*, 2015; Kalebic *et al*, 2018; Heide *et al*, 2020; Xing *et al*, 2021)—an increase in bRG abundance.

### Increased abundance of cycling BPs in chimpanzee cerebral organoids upon *ARHGAP11B* expression is initially associated with reduced deep‐layer neuron generation but eventually results in increased upper‐layer neuron generation

**Figure EV3:**
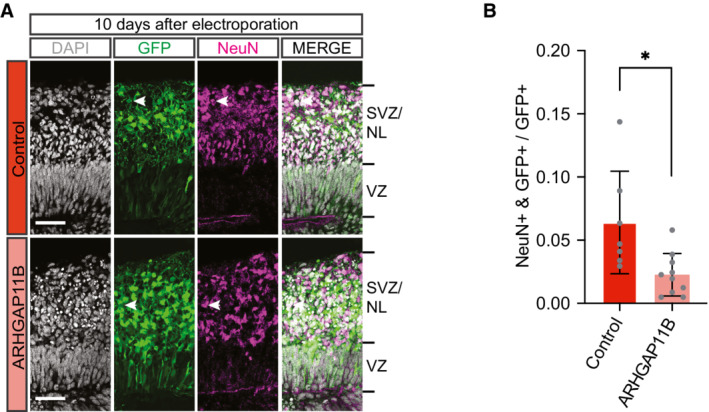
Expression of *ARHGAP11B* in chimpanzee cerebral organoids decreases the abundance of NeuN‐positive cells Double immunofluorescence for GFP (green) and the neuron marker NeuN (magenta), combined with DAPI staining (white), of a 61‐day‐old chimpanzee cerebral organoid 10 days after electroporation with GFP expression plasmid plus either control plasmid (top) or *ARHGAP11B* expression plasmid (bottom). Tick marks indicate the borders of the VZ and SVZ/NL; arrowheads indicate examples of GFP+ and NeuN+ double‐positive cells. Scale bars, 50 μm.Quantification of the proportion of GFP+ cells that are NeuN+ in 61‐day‐old chimpanzee cerebral organoids 10 days after electroporation with GFP expression plasmid plus either control plasmid (dark red) or *ARHGAP11B* expression plasmid (light red). Data are the mean of seven control and 10 *ARHGAP11B*‐transfected cerebral organoids of two independent batches each; error bars indicate SD; *P < 0.05 (two‐sided Student's *t*‐test).

If the observed increase in the abundance of cycling BPs (Fig EV2A and B) in chimpanzee cerebral organoids upon *ARHGAP11B* expression reflected indeed an increased proliferation of BPs, that is, BPs dividing to generate more BPs rather than neurons, one would expect a concomitant reduction in the generation of neurons from these cNPCs, as implied in our interpretation of the data in Figs 1 and 2. We explored this possibility by performing immunohistochemistry for neuronal markers 10 days after electroporation, to allow neuron generation from BPs to proceed. Compared with control, expression of *ARHGAP11B* in chimpanzee cerebral organoids resulted in a reduction in the proportion of the GFP‐positive progeny of the targeted APs that were positive for the neuron markers Hu (Fig 4A and B) and NeuN (Fig EV3A and B). This reduction by ≈ 10% points was consistent with the increases by in BPs and bRGs observed in Figs 1 and 2. In line with this GFP‐positive progeny being neurons, the majority of the Hu‐positive (Fig 4A) and NeuN‐positive (Fig EV3A) cells were located basal to the SVZ in the NL. We conclude that the increased abundance of cycling BPs observed after a 10‐day period of expression of *ARHGAP11B* in chimpanzee cerebral organoids reflects an increased generation of BPs from BPs, at the expense of—and hence resulting in a reduction in—the generation of cortical neurons from BPs during this time period.

In the developing neocortex, the generation of cortical neurons begins with the production of deep‐layer neurons, followed by the production of upper‐layer neurons (Molyneaux *et al*, 2007; Cooper, 2008; Agirman *et al*, 2017). In light of the decrease in neuron generation upon *ARHGAP11B* expression in chimpanzee cerebral organoids, we investigated whether this decrease applied to the generation of deep‐layer neurons, by performing immunohistochemistry for the deep‐layer neuron marker CTIP2 4 days after the electroporation of the organoids. This time period should be just sufficient for two sequential rounds of cNPC division, (i) targeted APs generating GFP‐positive BPs, and (ii) GFP‐positive BPs generating either GFP‐positive neurons (control) or GFP‐positive BPs (*ARHGAP11B*). Quantification of the occurrence of CTIP2 in GFP+ cells indicated that compared with control, expression of *ARHGAP11B* in chimpanzee cerebral organoids resulted in a reduction in the proportion of the GFP‐positive progeny of the targeted APs in the SVZ and NL that were positive for this deep‐layer neuron marker (Fig 4C and D). We conclude that the decrease in neuron generation observed upon *ARHGAP11B* expression in chimpanzee cerebral organoids involves the generation of deep‐layer neurons.

Previous studies on *ARHGAP11B* showed that its expression in developing mouse (Xing *et al*, 2021), ferret (Kalebic *et al*, 2018), and marmoset (Heide *et al*, 2020) neocortex can increase the abundance of upper‐layer neurons—the cortical neuron type that expanded disproportionally during primate evolution (Hutsler *et al*, 2005; Molnar *et al*, 2006; Fame *et al*, 2011). Hence, the question arises if expression of *ARHGAP11B* in chimpanzee cerebral organoids can eventually increase the generation of upper‐layer neurons. To address this, we extended the time period between electroporation and analysis from 10 to 15 days (Fig 1A), as 10 days after electroporation GFP‐positive cells were barely colocalizing with the upper‐layer neuron marker SATB2 (Appendix Fig S3).

The rationale for this extension was twofold. First, it can be assumed that due to the repeated divisions of cNPCs during this 15‐day period, notably after 10 days (see Fig EV1), the original level of the *ARHGAP11B* expression plasmid introduced into the targeted APs by electroporation and inherited by the BPs generated therefrom would decline over time, resulting in progressively lower ARHGAP11B levels in the BPs. This in turn should progressively reduce the tendency of BPs to undergo symmetric proliferative rather neuron‐generating divisions and increasingly promote the latter type of BP division. Second, during this 15‐day period, in particular after 10 days, the BPs should either switch from deep‐ to upper‐layer neuron production or at least start to produce also upper‐layer neurons. If so, the increase in BP abundance due to ARHGAP11B's action during the early phase of this 15‐day period should result in an increase in upper‐layer neuron production by the end of this period. To this end, the electroporated chimpanzee organoids were analyzed by immunofluorescence for the upper‐layer neuron marker SATB2. Indeed, compared with control, expression of *ARHGAP11B* in chimpanzee cerebral organoids resulted in an increase in the proportion of the GFP‐positive progeny of the targeted APs in the SVZ and NL that were positive for SATB2 (Fig 4E and F). In line with the data shown in Fig 4D, this increase likely occurred at the expense of deep‐layer neuron production. We conclude that expression of *ARHGAP11B* in chimpanzee cerebral organoids eventually leads to an increase in upper‐layer neuron generation.

We have so far implied that the observed decrease in deep‐layer neuron production observed 4 days after *ARHGAP11B* expression and the observed increase in upper‐layer neuron production 15 days after electroporation are linked to *ARHGAP11B*'s action in BPs that fades with time. Could these changes in deep‐layer vs. upper‐layer neuron levels be also, or perhaps only, be due to an effect of *ARHGAP11B* on neuronal fate? We find this scenario unlikely, as all previous studies from our lab have shown that *ARHGAP11B*, consistent with its action in mitochondria to increase glutaminolysis, only affects cycling cells, not post‐mitotic cells such as neurons (Florio *et al*, 2015, 2016; Kalebic *et al*, 2018; Heide *et al*, 2020; Namba *et al*, 2020; Xing *et al*, 2021).

### Dominant‐negative inhibition of ARHGAP11B's function in human cerebral organoids reduces cycling BPs to the chimpanzee level

The increase in cycling BP levels upon *ARHGAP11B* expression in chimpanzee cerebral organoids (Figs 1 and 2) is fully consistent with previous findings in another primate model, i.e., transgenic marmoset fetuses with physiological‐like *ARHGAP11B* expression (Heide *et al*, 2020), and provides strong further support for the notion that this human‐specific gene is a prime candidate to have caused neocortex expansion in the course of human evolution. We chose two approaches to determine to which extent the cycling BP levels in human cerebral cortex tissue depend on *ARHGAP11B* and hence to gain insight into *ARHGAP11B's* contribution to the expansion of the human neocortex during development and evolution.

**Figure 5:**
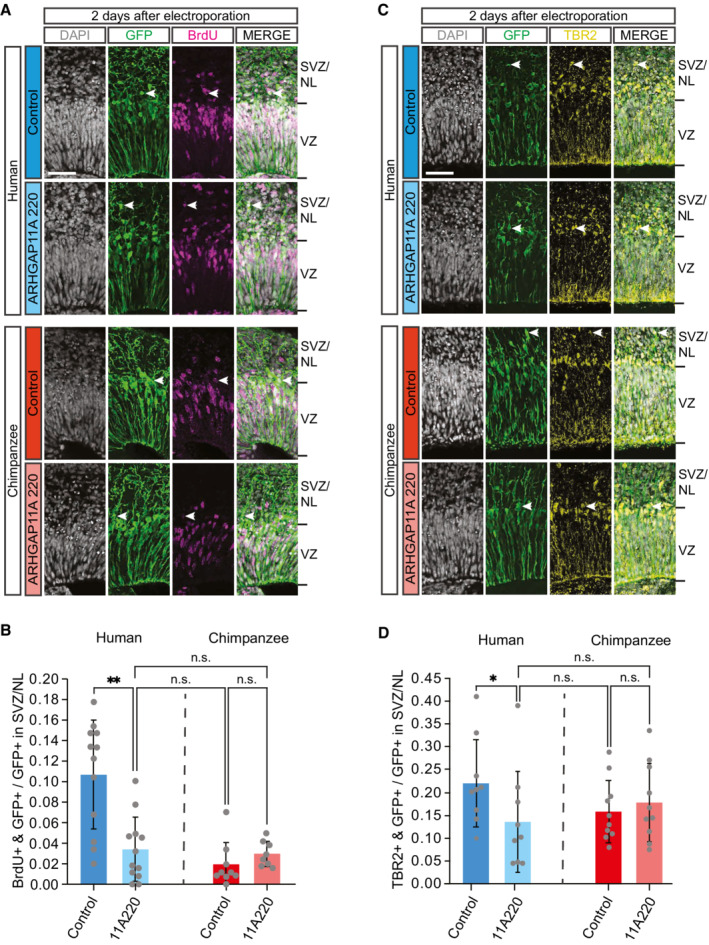
Dominant‐negative ARHGAP11A220 reduces the level of cycling BPs in ARHGAP11B‐expressing human cerebral organoids to the chimpanzee level but has no effect on BP levels in chimpanzee cerebral organoids Double immunofluorescence for GFP (green) and the thymidine analog BrdU (magenta, see Fig 1A), combined with DAPI staining (white), of 57‐day‐old human (top panels) and chimpanzee (bottom panels) cerebral organoids 2 days after electroporation with GFP expression plasmid plus either control plasmid (upper panels) or *ARHGAP11A220* expression plasmid (lower panels). Tick marks indicate the borders of the VZ and SVZ/NL; arrowheads indicate examples of GFP+ and BrdU+ double‐positive cells. Scale bar, 50 μm.Quantification of the proportion of GFP+ cells in the SVZ/NL that are BrdU+ in 57‐day‐old human (blue) and chimpanzee (red) cerebral organoids 2 days after electroporation with GFP expression plasmid plus either control plasmid (dark blue and dark red) or *ARHGAP11A220* expression plasmid (light blue and light red). Data are the mean of 12 human control, 12 human *ARHGAP11A220*‐transfected, 9 chimpanzee control, and 8 chimpanzee *ARHGAP11A220*‐transfected cerebral organoids (grown from one human and one chimpanzee iPSC line, respectively) of six (human) and four (chimpanzee) independent batches each; error bars indicate SD; n.s., not significant; **P < 0.01 (Kruskal–Wallis test).Double immunofluorescence for GFP (green) and TBR2 (yellow), combined with DAPI staining (white), of 57‐day‐old human (top panels) and chimpanzee (bottom panels) cerebral organoids 2 days after electroporation with GFP expression plasmid plus either control plasmid (upper panels) or *ARHGAP11A220* expression plasmid (lower panels). Tick marks indicate the borders of the VZ and SVZ/NL; arrowheads indicate examples of GFP+ and TBR2+ double‐positive cells. Scale bar, 50 μm.Quantification of the proportion of GFP+ cells in the SVZ/NL that are TBR2+ in 57‐day‐old human (blue) and chimpanzee (red) cerebral organoids 2 days after electroporation with GFP expression plasmid plus either control plasmid (dark blue and dark red) or *ARHGAP11A220* expression plasmid (light blue and light red). Data are the mean of 9 human control, 9 human *ARHGAP11A220*‐transfected, 10 chimpanzee control, and 10 chimpanzee *ARHGAP11A220*‐transfected cerebral organoids (grown from one human and one chimpanzee iPSC line, respectively) of four (human) and three (chimpanzee) independent batches each; error bars indicate SD; n.s., not significant; *P < 0.05 (Kruskal–Wallis test).

First, we made use of a truncated form of the ARHGAP11A protein (ARHGAP11A220) that has previously been shown to act in a dominant‐negative manner on ARHGAP11B's ability to amplify BPs (Namba *et al*, 2020). This dominant‐negative action on ARHGAP11B and not on ARHGAP11A can be explained by the following two findings. (i) ARHGAP11A220, similar to ARHGAP11B and in contrast to full‐length ARHGAP11A, localizes to mitochondria and not (like ARHGAP11A) to the nucleus (Namba *et al*, 2020). (ii) ARHGAP11A220, via its truncated GAP domain, can interact with the same downstream effector system in mitochondria as ARHGAP11B, however, without being able to change its activity, which requires the human‐specific C‐terminal domain of ARHGAP11B (Namba *et al*, 2020). We examined the effects of ARHGAP11A220 on cycling BP levels in human cerebral organoids 2 days after electroporation of the corresponding expression plasmid, with a 1 h BrdU pulse prior to fixation (Fig 1A). The pattern of SOX2 immunostaining was used to distinguish cNPCs in the VZ vs. SVZ and to attribute the GFP‐positive progeny to either of these two germinal zones (Fig 1D). We found that compared with control, transfection of the cNPCs in the VZ of human cerebral organoids with the dominant‐negative ARHGAP11A220 resulted in a marked reduction, virtually down to the level observed in chimpanzee cerebral organoids, in the proportion of the GFP‐positive progeny of the targeted APs found in the SVZ that had incorporated BrdU (Fig 5A and B). As inhibition of ARHGAP11B function is not known to result in a shortening of S‐phase (and hence in reduced BrdU incorporation), these data suggest that inhibition of ARHGAP11B function reduces the level of cycling BPs. We therefore conclude that *ARHGAP11B* is required to maintain the elevated level of cycling BPs in human cerebral organoids.

To corroborate this conclusion, we analyzed the *ARHGAP11A220*‐transfected human cerebral organoids for GFP‐positive cells containing the BP marker TBR2, which in fetal human neocortex is typically expressed in the bIP subpopulation of BPs (Fietz *et al*, 2010). Transfection of the human cerebral organoids with ARHGAP11A220 caused after 2 days a reduction down to half of control in the proportion of the GFP‐positive progeny of the targeted APs in the SVZ and NL that were TBR2‐positive (Fig 5C and D). This decrease in the proportion of BPs among the GFP+ cells in SVZ and NL implied an apparent increase in the proportion of TBR‐negative cells, which we believe reflected an actually constant proportion of neurons among the GFP+ cells in SVZ and NL due to a decrease in the absolute pool size of GFP+ cells. Interestingly, for the human and chimpanzee iPSC lines used in the present study to generate cerebral organoids, this reduction brought the level of the BrdU+ BPs (Fig 5B) and the TBR2+ BPs (Fig 5D) down to that observed in control chimpanzee cerebral organoids. We therefore conclude that *ARHGAP11B* is required to maintain the elevated level of cycling BPs that is characteristic of human cerebral organoids.

### ARHGAP11A220 specifically inhibits ARHGAP11B function

We electroporated *ARHGAP11A220* into chimpanzee cerebral organoids, which lack ARHGAP11B, to determine whether the effects of ARHGAP11A220 observed in human cerebral organoids were specific for ARHGAP11B. Indeed, 2 days after transfection of the APs in the VZ of chimpanzee cerebral organoids with *ARHGAP11A220* vs. control, we observed no change in the proportion of the GFP‐positive progeny of the targeted APs in the SVZ, i.e., of BPs, that had incorporated BrdU (Fig 5A and B), or that were TBR2‐positive (Fig 5C and D). Hence, the reduction in the level of cycling BPs observed upon transfection of human cerebral organoids with the dominant‐negative *ARHGAP11A220* reflected a specific inhibition of ARHGAP11B's ability to amplify BPs.

### Knockout of *ARHGAP11B* in human forebrain organoids results in reduced bRG abundance

**Figure EV4:**
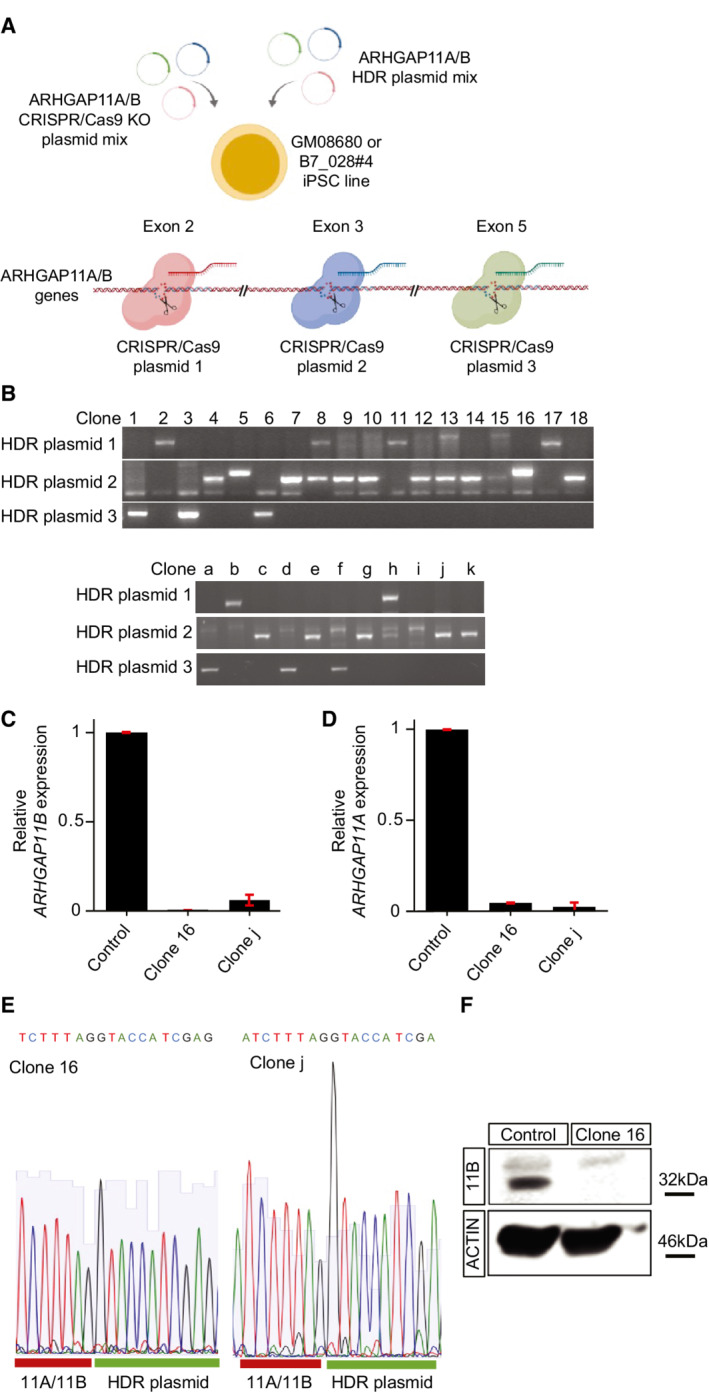
Validation of the human *ARHGAP11A* plus *ARHGAP11B* double‐knockout iPSC clones 16 and j A
Schematic overview of the knockout strategy (image created with BioRender.com). A mixture of three CRISPR/Cas9 plasmids, each encoding the gRNA targeting either exon 2, 3, or 5 and SpCas9 ribonuclease, and three Homology‐Directed Repair (HDR) plasmids, each containing the corresponding homology arms and encoding a puromycin resistance cassette under the control of the EF‐1α promoter, were nucleofected into human GM08680 and B7_028#4 iPSCs. Due to the targeting by the CRISPR/Cas9 plasmids of the sites in exon 2, 3, and 5 of the *ARHGAP11A* and/or *ARHGAP11B* genes, each of these sites should show integration of the corresponding HDR plasmid.B
Genomic PCR validation of the indicated human iPSC clones for integration of the HDR plasmid. PCR primers were designed to recognize the integration of the HDR plasmid in exon 2 (HDR plasmid 1), exon 3 (HDR plasmid 2), or exon 5 (HDR plasmid 3) of *ARHGAP11B*. The image shows 18 of the 24 clones analyzed for the GM08680 iPSC line (clone 1–18) and 11 of the 14 clones analyzed for the B7_028#4 iPSC line (clone a‐k).C, D
Analysis of the relative expression levels of the *ARHGAP11B* (C) and *ARHGAP11A* (D) mRNAs by q‐RT‐PCR of control, clone 16, and clone j iPSCs, showing virtually complete lack of expression for both genes in clone 16 and clone j, indicative of a homozygous *ARHGAP11A* plus *ARHGAP11B* double‐knockout. Data are the mean of three independent experiments; error bars indicate SD.E
Sequencing analysis of clone 16 and clone j iPSCs confirmed the disruption of the *ARHGAP11A* plus *ARHGAP11B* genes in exon 3 in both clones due to the integration of the HDR plasmid, indicative of a double‐knockout.F
Immunoblot for ARHGAP11B (11B, top) and actin (bottom) of cleared lysates from control (left) and clone 16 (right) iPSCs.

The second approach to determine the contribution of *ARHGAP11B* in the maintenance of the elevated level of cycling BPs that is characteristic of human cerebral cortex tissue was to generate a homozygous *ARHGAP11B* knockout using the CRISPR/Cas9 technology. However, due to the genomic near‐identity of *ARHGAP11A* and *ARHGAP11B* in exons 1–5, we were unable to find guide RNAs that would efficiently and specifically target only *ARHGAP11B*. We therefore decided to use a guide RNA mixture that targets also the *ARHGAP11A* gene. To this end, we made use of an organoid system distinct from the human cerebral organoids studied so far (Fig 1), that is, human forebrain organoids grown from the human iPSC lines GM08680 (Iefremova *et al*, 2017) and B7_028#4 (Jabali *et al*, 2022). Specifically, we subjected GM08680 and B7_028#4 cells to CRISPR/Cas9‐mediated gene disruption. Three sites in exons 2, 3, and 5 of the *ARHGAP11A* and *ARHGAP11B* genes were targeted in a combined approach (Fig EV4A). Out of more than 70 picked iPSC clones, 38 survived puromycin selection and showed integration of the homology‐directed repair (HDR) plasmid (shown for 18 clones of the line GM08680 and 11 of the line B7_028#4 in Fig EV4B). When investigating the expression levels of the *ARHGAP11B* mRNA; however, we found only one clone per line (clone #16 for GM08680 and clone j for B7_028#4) exhibiting virtually no *ARHGAP11B* gene expression (Fig EV4C). These clones also showed a massively reduced *ARHGAP11A* expression level (Fig EV4D), suggesting a homozygous *ARHGAP11A* and *ARHGAP11B* double‐knockout. DNA sequencing confirmed the double‐knockout in clone 16 and in clone j by showing the disruption of the *ARHGAP11A* and *ARHGAP11B* coding sequences due to the integration, in exon 3 of either gene, of the HDR plasmid containing a puromycin resistance cassette with a translational stop codon followed by a polyadenylation signal (Fig EV4E). Immunoblot analysis of the ARHGAP11B protein level corroborated the lack of detectable ARHGAP11B protein, in contrast to control iPSCs, as shown for clone 16 cells in Fig EV4F. In addition, we investigated potential off‐target integrations of the gene‐editing by Whole Genome Sequencing (Datasets [Link], [Link], [Link]). None of the potential off‐target integrations were identical between the two *ARHGAP11A* and *ARHGAP11B* double‐knockout clones (Dataset EV1), indicating that any effect observed for both *ARHGAP11A* and *ARHGAP11B* double‐knockout clones would not be due to an off‐target integration but rather specific to the double knockout.

**Figure 6:**
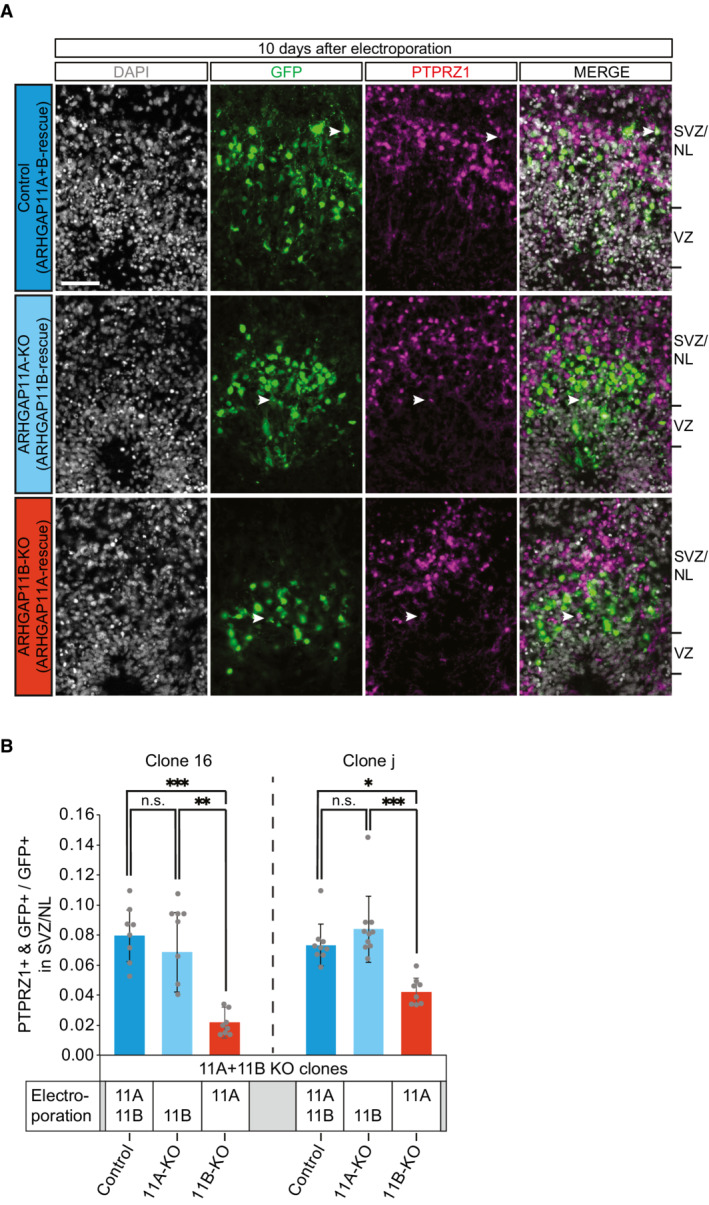
*ARHGAP11B* knockout leads to a substantial reduction of bRG abundance in human forebrain organoids Double immunofluorescence for GFP (green) and the bRG marker PTPRZ1 (magenta), combined with DAPI staining (white), of 60‐day‐old human forebrain organoids, generated from *ARHGAP11A* plus *ARHGAP11B* double‐knockout clone 16. Images were obtained 10 days after electroporation with GFP expression plasmid plus either *ARHGAP11A* plus *ARHGAP11B* expression plasmids (ARHGAP11A + B‐rescue, referred to as Control), *ARHGAP11B* expression plasmid only (ARHGAP11B‐rescue, referred to as ARHGAP11A‐KO), or *ARHGAP11A* expression plasmid only (ARHGAP11A‐rescue, referred to as ARHGAP11B‐KO). Tick marks indicate the borders of the VZ and SVZ/NL; arrowheads indicate examples of GFP+ and PTPRZ1+ double‐positive cells. Scale bar, 50 μm.Quantification of the proportion of GFP+ cells in the SVZ/NL that are PTPRZ1+ in 60‐day‐old *ARHGAP11A* plus *ARHGAP11B* double‐knockout (11A + 11B KO) human forebrain organoids. Organoids were generated from clone 16 (left) and clone j (right), and data were obtained 10 days after the indicated electroporation with GFP expression plasmid plus either *ARHGAP11A* (11A) plus *ARHGAP11B* (11B) expression plasmids (referred to as Control, as this constitutes an ARHGAP11A+B double‐rescue in the 11A+11B KO organoids; dark blue), *ARHGAP11B* (11B) expression plasmid only (referred to as 11A‐KO, as this constitutes a selective ARHGAP11B rescue in the 11A+11B KO organoids; light blue), or *ARHGAP11A* (11A) expression plasmid only (referred to as 11B‐KO, as this constitues a selective ARHGAP11A rescue in the 11A+11B KO organoids; dark red). For clone 16, data are the mean of eight control, eight 11A‐KO, and eight 11B‐KO human forebrain organoids of two independent batches each; for clone j, data are the mean of 9 control, 10 11A‐KO, and 8 11B‐KO human forebrain organoids of three independent batches each; error bars indicate SD; n.s., not significant; *P < 0.05; **P < 0.01; ***P < 0.001 (Kruskal–Wallis test).

We used clone 16 and clone j iPSCs to grow forebrain organoids according to a protocol previously established in one of our labs (Iefremova *et al*, 2017; Krefft *et al*, 2018). Ventricle‐like structures of day‐50 forebrain organoids were then subjected to electroporation with the GFP‐expressing plasmid and either (i) the *ARHGAP11A* plus *ARHGAP11B* expression plasmids, (ii) the *ARHGAP11B* expression plasmid only or (iii) the *ARHGAP11A* expression plasmid only. We considered the overexpression of ARHGAP11A plus ARHGAP11B in *ARHGAP11A* plus *ARHGAP11B* double‐knockout organoids to serve as control by constituting an *ARHGAP11A* plus *ARHGAP11B* double rescue. Accordingly, we considered the overexpression of ARHGAP11B in *ARHGAP11A* plus *ARHGAP11B* double‐knockout organoids to provide a selective *ARHGAP11A* knockout by constituting an *ARHGAP11B* rescue. Likewise, we considered the overexpression of ARHGAP11A in *ARHGAP11A* plus *ARHGAP11B* double‐knockout organoids to provide a selective *ARHGAP11B* knockout by constituting an *ARHGAP11A* rescue. In line with our previous finding in the *ARHGAP11B* overexpression experiments in chimpanzee cerebral organoids, which showed that the effect on bRG levels is stronger 10 days than 4 days after electroporation (Fig 3B and D), we performed immunohistochemistry for the bRG marker PTPRZ1 (Pollen *et al*, 2015) 10 days after electroporation (Fig 6A).

These experiments revealed a substantial decrease in the proportion of the GFP‐positive progeny of the targeted APs found in the SVZ that were positive for PTPRZ1, in the *ARHGAP11B* knockout forebrain organoids grown from the two independent iPSC clones in comparison to the respective control (Fig 6B). No significant difference between control and *ARHGAP11A* knockout forebrain organoids was observed. Moreover, overexpression of ARHGAP11A, ARHGAP11B, or a combination of ARHGAP11A plus ARHGAP11B in human forebrain organoids grown from control iPSCs did not result in significantly increased bRG levels (Appendix Fig S4; for a comparison of bRG levels between double‐knockout organoids and organoids grown from control iPSCs, see Appendix Fig S5). These data (Appendix Fig S4) suggested that when using the *ARHGAP11A* plus *ARHGAP11B* double‐knockout forebrain organoids, the reduced bRG abundance in the *ARHGAP11B* knockout (ARHGAP11A overexpression) organoids in comparison to the *ARHGAP11A* knockout (ARHGAP11B overexpression) or control (ARHGAP11A plus ARHGAP11B overexpression) organoids did not involve an ARHGAP11B overexpression effect in the latter two types of electroporated organoids. We conclude that the lower bRG abundance in the *ARHGAP11B* knockout forebrain organoids as compared with the control forebrain organoids indicates a major role of *ARHGAP11B* in the maintenance of the bRG level in human forebrain organoids. Together with the results of the dominant‐negative ARHGAP11A220 in human cerebral organoids (Fig 5), these data demonstrate that *ARHGAP11B* is required to maintain the elevated levels of cycling BPs, notably bRG, which are characteristic of human cerebral cortex tissue.

## Discussion

In the present study, the use of human and chimpanzee cerebral organoids and human forebrain organoids has allowed us to answer two key questions about the role of the human‐specific gene *ARHGAP11B* in the expansion of the human neocortex in development and evolution. First, is *ARHGAP11B* essential to maintain the elevated levels of cycling BPs that are characteristic of human cerebral cortex tissue and that are thought to be required for human neocortex expansion? Second, is *ARHGAP11B* sufficient to cause such elevated levels of cycling BPs?

We have addressed these questions using brain organoids (discussed below) and an electroporation‐based experimental protocol. Two technical aspects need to be considered using the latter approach. First, the APs would experience the highest *ARHGAP11B* expression due to the use of the strong constitutive CAGGS promoter in combination with a high amount of plasmid, which these cells—as primary targets of electroporation—would receive. However, APs have been found to not show an increase in their pool size upon *ARHGAP11B* expression (Florio *et al*, 2015), indicating that the receptiveness of the progenitor cell type (AP vs. BP) rather than changes in the *ARHGAP11B* expression level is the major determinant for ARHGAP11B's effects. Second, with each cNPC division the number of plasmids in the daughter cells would be diluted resulting in a reduced *ARHGAP11B* expression in the daughter cells. However, the ARHGAP11B protein generated from the expression plasmid will also be inherited by the progeny of the transfected cells. Together with the inheritance of the expression plasmid, this should ensure sufficient ARHGAP11B protein levels in the progeny of the transfected cells to drive the machinery downstream of ARHGAP11B. Consistent with this assumption, we observe the effect of *ARHGAP11B* expression on the level of BPs, notably bRG, that is, their doubling, equally at 2, 4, and 10 days post‐electroporation, although several rounds of progenitor cell division will have occurred between day 2 and 10.

Moreover, the use of human and chimpanzee iPSCs, which were used here to generate brain organoids, has unique advantages for addressing the two questions specified above. First, the use of chimpanzee cerebral organoids has allowed us to determine, in the evolutionarily closest living species to human, *ARHGAP11B*'s role in ensuring the elevated cycling BP levels implicated in neocortex expansion, and hence the likely contribution of this human‐specific gene to neocortex expansion during hominid evolution. Another human‐specific gene, *NOTCH2NL*, which when expressed in embryonic mouse neocortex increases cycling BPs (Florio *et al*, 2018), has previously been studied in human and mouse brain organoids (Fiddes *et al*, 2018). This approach provided important insight into the function of NOTCH2NL and its potential role in neocortex expansion (Fiddes *et al*, 2018). However, to precisely determine the contribution of a human‐specific gene to human neocortex expansion, it is necessary to study this gene in a model system that is evolutionarily as close as possible to humans. With regard to the human‐specific gene *ARHGAP11B*, besides previous studies in developing mouse (Florio *et al*, 2015, 2016; Xing *et al*, 2021) and ferret (Kalebic *et al*, 2018) neocortex, the model system closest to human studied so far has been the fetal neocortex of the common marmoset (Heide *et al*, 2020), a non‐human primate. However, when one considers the time point of origin of *ARHGAP11B* in the human lineage ≈ 5 mya (Sudmant *et al*, 2010; Dennis *et al*, 2017), that is, shortly after the split from the lineage leading to the chimpanzee and bonobo ≈ 7 mya (Brunet *et al*, 2005, 2002), the marmoset is evolutionarily quite distant from human, as the split of the lineage leading to hominins from the lineage leading to the marmoset happened ≈ 40 mya. While our previous expression of *ARHGAP11B* in fetal marmoset neocortex made the point that *ARHGAP11B* can expand the primate neocortex (Heide *et al*, 2020), the present expression of *ARHGAP11B* in chimpanzee cerebral organoids provides insight into the actual contribution of this human‐specific gene to neocortex expansion during hominid evolution. Thus, our finding that *ARHGAP11B* expression in chimpanzee cerebral organoids results in a doubling of cycling BP levels demonstrates that *ARHGAP11B* alone is capable of increasing the abundance of cycling BPs to a human‐like level and hence of providing a crucial basis for the evolutionary expansion of the human neocortex.

Second, with regard to the human brain organoids used here, which have been shown to recapitulate many key features of fetal human neocortical tissue (Kadoshima *et al*, 2013; Lancaster *et al*, 2013, 2017; Qian *et al*, 2016; Quadrato *et al*, 2017; Heide *et al*, 2018; Karzbrun *et al*, 2018; Giandomenico *et al*, 2019), this model system provides a readily available source of human neocortex‐like tissue to investigate *ARHGAP11B*'s role during human neocortex development. Compared with fetal human neocortical tissue that can be obtained in principle, albeit only at an early stage of neocortex development, and studied *ex vivo*, human brain organoids offer a broader range of developmental stages. Moreover, because they originate from iPSCs, human brain organoids allow modes of genetic manipulation that are not possible with fetal human neocortical tissue *ex vivo*, such as the comprehensive ablation of a gene of interest, a potential we have exploited. Also, studying the long‐term effects of a manipulation, such as the effects of *ARHGAP11B* 15 days after electroporation into human brain organoids as done here, would be very difficult, if not impossible, with fetal human neocortical tissue *ex vivo*. We have made use of these advantages and have employed human brain organoids to investigate to which extent *ARHGAP11B* is necessary to maintain the elevated levels of cycling BPs that are characteristic of fetal human neocortex (Lui *et al*, 2011; Florio & Huttner, 2014). We find that interference with ARHGAP11B's function results in a drastic decrease in the level of cycling BPs, down to that observed in chimpanzee cerebral organoids. Moreover, a selective *ARHGAP11B* knockout, achieved by an *ARHGAP11A* rescue in *ARHGAP11A* plus *ARHGAP11B* double‐knockout forebrain organoids, resulted in a massive decrease in bRG, i.e., BP, abundance. Together, these data demonstrate that *ARHGAP11B* is essential for maintaining the elevated levels of cycling BPs as found in human cerebral cortical tissue and characteristically in fetal human neocortex and imply that this human‐specific gene was indispensable for the expansion of the neocortex during hominid evolution.

## Materials and Methods

### Cell culture and generation of brain organoids

Human SC102A‐1 (System Bioscience) and chimpanzee Sandra A iPSC lines (Camp *et al*, 2015; Mora‐Bermudez *et al*, 2016; Kanton *et al*, 2019) were cultivated using standard feeder‐free conditions in mTeSR1 (StemCell Technologies) on Matrigel (Corning)‐coated plates and differentiated into cerebral organoids using previously published protocols (Lancaster *et al*, 2013; Lancaster & Knoblich, 2014; Camp *et al*, 2015; Mora‐Bermudez *et al*, 2016; Kanton *et al*, 2019) (Fig 1A). Briefly, 10,000 cells per well were seeded into 96‐well ultra‐low attachment plates (Corning) in mTeSR containing 50 μM Y27632 (StemCell Technologies). Medium was changed after 48 h to mTeSR without Y27632. On day 5 after seeding, medium was changed to neural induction medium (DMEM/F12 (Gibco) containing 1% N2 supplement (Gibco), 1% Glutamax supplement (Gibco), 1% MEM non‐essential amino acids (Gibco), and 1 μg/ml heparin (Sigma‐Aldrich)) and changed every other day. On day 9 or 10 after seeding, embryoid bodies were embedded in Matrigel, transferred to differentiation medium (1:1 DMEM/F12 (Gibco)/Neuralbasal (Gibco) containing 0.5% N2 supplement (Gibco), 0.025% insulin solution (Sigma‐Aldrich), 1% Glutamax supplement (Gibco), 0.5% MEM non‐essential amino acids (Gibco), 1% B27 supplement (without vitamin A, Gibco), 1% penicillin–streptomycin, and 0.00035% 2‐mercaptoethanol (Merck)), and placed on an orbital shaker. Medium was changed every other day and on day 16 after seeding switched to differentiation medium containing B27 supplement with vitamin A (Gibco). Cerebral organoids were further cultured in this differentiation medium until fixation, with medium changes every 3 days and electroporation and (if applicable) BrdU labeling as indicated (see Fig 1A). All cell and organoid cultures were performed in an incubator in a humidified atmosphere of 5% CO_2_, 95% air at 37°C.

In the case of *ARHGAP11A* plus *ARHGAP11B* double‐knockout experiments, human forebrain organoids were generated from the iPSC lines GM08680 (Iefremova *et al*, 2017) and B7_028#4 (44‐year‐old healthy female derived with given informed consent within the collaborative research center project SFB636 B7) and the respective *ARHGAP11A* plus *ARHGAP11B* double‐knockout lines (see KO description below), according to previously published protocols (Iefremova *et al*, 2017; Krefft *et al*, 2018) with the following minor modifications. At day 35, organoid cultures were switched to maturation medium (DMEM/F12 (Gibco) containing 1% N2 supplement (Gibco), 1% B27 with vitamin A (Gibco), 1% Glutamax supplement (Gibco), 1% MEM non‐essential amino acids (Gibco), 0.8 ng/ml glucose (Carl Roth GmbH&co.), 0.15 μg/ml cAMP (Sigma‐Aldrige), 0.1 nM ß‐mercaptoethanol (Gibco), 0.01 μg/ml GDNF (Cell Guidance Systems Ltd), 1 μM LM22A (Sigma‐Aldrich), 1 μM LM22B (Tocris Bioscience), 1.25 μg/ml insulin, 0.2 μM ascorbic acid (Cell Guidance Systems Ltd), 0.1% GelTrex™ (Gibco)). At day 40, the forebrain organoids were sliced according to Qian *et al* (2020). In brief, forebrain organoids were immersed in 4% low‐melting‐point agarose dissolved in DMEM/F12 (Gibco) in a 1.5‐cm wide mold. The block was cooled down for 10 min on ice to solidify and then glued on a vibratome (Microm HM650V, Thermo Fisher Scientific). The samples were sliced into 400‐μm thick sections at 0.1 mm/s speed and 100 Hz frequency. The sections were transferred into a 6‐cm dish with maturation medium. One day later, the medium was changed and the dish was placed on an orbital shaker and maintained in culture, with medium change every 3 days. On day 47, the forebrain organoid cultures were transferred in pre‐warmed and gassed maturation medium from the Mannheim institute to MPI‐CBG, where they received fresh maturation medium followed by culture until day 50. On day 50, the forebrain organoids were subjected to electroporation as is described below, followed by culture in maturation medium until fixation at day 60, with medium changes every 3 days. Forebrain organoids not subjected to electroporation were cultured until day 55 and then fixed. Except for the 18‐h transfer between institutions, all cell and organoid cultures were performed in an incubator in a humidified atmosphere of 5% CO_2_, 95% air at 37°C.

### Generation and validation of *ARHGAP11A* plus *ARHGAP11B* double‐knockout iPSCs

*ARHGAP11A* plus *ARHGAP11B* double‐knockout iPSCs were generated using a commercial kit (Santa Cruz). This kit is composed of a mixture of three different CRISPR/Cas9 knockout plasmids and three different Homology‐Directed Repair (HDR) plasmids. Specifically, the CRISPR/Cas9 knockout plasmids constitute a mixture of three plasmids, each encoding a different gRNA targeting exon 2, 3, or 5 and a SpCas9 ribonuclease. The three HDR plasmids contain HDR arms specific for each of the three gRNAs, and a puromycin resistance gene under the control of the EF‐1α promoter flanked by loxP sites. Use of this plasmid mixture would be expected to result in site‐specific double‐strand breaks followed by integration of the respective HDR plasmid, leading to the disruption of exon 2, 3, and/or 5 of the *ARHGAP11A* and/or *ARHGAP11B* genes. In brief, 1.5x10^6^ iPSCs per line were transfected with the three different CRISPR/Cas9 knockout plasmids (1 μg in total) directed against exons 2, 3, or 5 of the *ARHGAP11A* and *ARHGAP11B* genes, together with the corresponding three HDR plasmids (1 μg in total), using the Nucleofector™2b (Lonza) and the Cell Line Nucleofector^®^ Kit V (Lonza) according to the manufacturer's protocol. Following nucleofection, cells were plated on Geltrex‐coated cell culture plates in E8 medium (Thermo Fisher Scientific) supplemented with 5 μM Y‐27632. Puromycin (1 μg/ml, Merck Milipore) selection was initiated 48 h following transfection. Clones were picked manually 5–12 days following nucleofection and transferred into Geltrex‐coated 48‐well cell culture plates. Integration of the HDR plasmids was validated by PCR of genomic DNA. To this end, genomic DNA was isolated using the Extractme genomic DNA kit (7Bioscience) according to the manufacturer's protocol. PCR primers were designed such that one would be complementary to the *ARHGAP11A/B* wild‐type alleles and the other to the HDR plasmid. *ARHGAP11B* and *ARHGAP11A* gene expression was investigated using q‐RT‐PCR. To this end, RNA was isolated from each of the clones using peqGOLD TriFast (VWR) following the supplier's instructions. The iScript cDNA synthesis kit (BioRad) was used for the reverse transcription of 1 μg RNA following the manufacturer's protocol. Each primer pair was first analyzed, using Taq polymerase (Biozym), for specific DNA amplification, and the PCR conditions and cycle numbers were optimized, using cDNA obtained from commercially available human fetal brain (single donor, female, 19 weeks of gestation). For quantitative RT‐PCR (qRT‐PCR), the PCR products were generated and analyzed by the QuantStudio7 Flex Real‐Time PCR System (Thermo Fisher Scientific) with SYBR®Green detection. The data were normalized to the 18S rRNA level using the ΔΔ Ct method. The primers used were: ARHGAP11B_cas_1, forward, aggtctgtagtgcttggcctg; reverse, aaggagagatgcgagcccct; ARHGAP11B_cas_2, forward, gtggcgaacgagggtcag; reverse, ggcaaacccgttgcgaaaaa; ARHGAP11B_cas_3, forward, tcacgtttggctccatctaat; reverse, actcaaccggcgtggatg; ARHGAP11AB_RT, forward, gctactccatcactggaaggc; reverse, gtgctccactaacaaaatctcct; ARHGAP11A_RT, forward, cgatacaagctcagaagggtca; reverse, gcttttcctgattccactctgc; ARHGAP11B_RT, forward, aactgccagagcccattctc; reverse, gtctggtacacgcccttcttt; 18S, forward, ttccttggaccggcgcaag; reverse, gccgcatcgccggtcgg.

Validation of the reduction in the ARHGAP11B protein level in KO‐iPSCs was performed by immunoblot analysis. In brief, control and KO‐iPSCs were washed twice with ice‐cold PBS, scraped off from the dish into PBS, and collected via centrifugation. Cell pellets were lysed in RIPA buffer (150 mM NaCl, 0.2% SDS (Carl Roth), 0.2% Triton X‐100 (Merck Milipore), 25 mM EDTA, 50 mM Tris–HCl pH 7.4) containing PierceTM protease inhibitor (Thermo Fisher Scientific), and PierceTM phosphatase inhibitor (Thermo Fisher Scientific) for 1 h on ice. Genomic DNA was fragmented, and cell and organelle lysis further promoted, by sonication (Branson Sonifier 250; 5 pulses, duty cycle 20%, output control 5.5). Residual cell and large organelle remnants were sedimented by centrifugation at 16,000 *g* for 15 min at 4°C. Protein concentration of cleared cell lysates was determined using the bicinchoninic acid (BCA) protein assay kit (Thermo Fisher Scientific). For immunoblotting, 20 μg of protein was boiled in 6× SDS sample buffer for 5 min at 95°C. Lysates were resolved on 10% gels and transferred onto 0.2 μm nitrocellulose membranes by semi‐dry blotting. Nitrocellulose membranes were blocked in 5% BSA in 1× TBST (25 mM Tris–HCl pH7.4, 137 mM NaCl, 2.7 mM KCl, 0.1% (v/v) Tween20) for 1 h at room temperature and subsequently incubated overnight with primary antibody in blocking solution at 4°C. The next day, membranes were washed three times with TBST, incubated with infrared fluorescent dye (IRDye)‐conjugated secondary antibodies (DyLight™, Cell Signaling Technology) diluted 1:15,000 in 1× TBST for 1 h at room temperature. Subsequently, membranes were washed three times in 1× TBST before visualization of the antigens of interest using an Odyssey IR imaging system (LI‐COR Biosciences). Primary antibodies and concentrations were as follows: anti‐ARHGAP11B (rabbit polyclonal, Origene, TA334130, 1:500), anti‐β‐actin (mouse monoclonal, Cell Signaling Technologies, 3700S, 1:10,000).

### *ARHGAP11B* expression construct

For the expression of *ARHGAP11B* in brain organoids, we used a previously described construct, pCAGGS‐*ARHGAP11B* (Florio *et al*, 2015). This construct encompasses *ARHGAP11B*'s coding sequence but excludes its untranslated regions as this would have increased the size of the expression plasmid and hence reduced the efficiency of electroporation. As this construct would produce high *ARHGAP11B* expression levels, we were also considering to use the previously described ≈3 kb human *ARHGAP11B* promoter (Heide *et al*, 2020) rather than the CAGGS promoter. However, we expected, in contrast to the lentivirus injection into oocytes in the marmoset study, the efficiency of *ARHGAP11B* expression upon electroporation of a human *ARHGAP11B* promoter–containing construct to be rather low. This low transfection efficiency would be caused either by the greater size of this construct as compared with the pCAGGS‐*ARHGAP11B* vector, which may reduce its entry into the transfected cells, or by the lack of efficient entry into the nucleus of the electroporated cells to reach the relevant transcription machinery. Furthermore, our previous reports studying the effects of *ARHGAP11B* expression using the constitutive CAGGS promoter in mouse and ferret embryos (Florio *et al*, 2015, 2016; Kalebic *et al*, 2018) vs. using the physiological human promoter in marmoset fetuses (Heide *et al*, 2020) have established that this is a valid approach to study the functions of ARHGAP11B.

### Electroporation of brain organoids

For cerebral organoid and forebrain organoid electroporation, organoids were placed in an electroporation chamber filled with pre‐warmed DMEM/F12 medium. Three to six ventricle‐like structures per organoid were microinjected with a solution containing 0.1% Fast Green (Sigma) in sterile PBS, 500 ng/μl of either empty pCAGGS vector (control; Florio *et al*, 2015), pCAGGS‐*ARHGAP11B* vector (Florio *et al*, 2015), pCAGGS‐*ARHGAP11A220* vector (Namba *et al*, 2020), or pCAGGS‐*ARHGAP11A* vector (Namba *et al*, 2020), in all cases together with 500 ng/μl pCAGGS‐*EGFP* (Florio *et al*, 2015). In the case of the *ARHGAP11A/B* double‐rescue experiments, a solution containing 0.1% Fast Green (Sigma) in sterile PBS, 250 ng/μl pCAGGS‐*ARHGAP11B* vector, 250 ng/μl pCAGGS‐*ARHGAP11A* vector, and 500 ng/μl pCAGGS‐*EGFP* was used. The ventricle‐like structures were microinjected with the Fast Green–containing solution until visibly filled. Electroporations were performed with five 50‐msec pulses of 80 V at 1 s intervals. Electroporated organoids were further cultured (i) in differentiation medium containing vitamin A for cerebral organoids, and (ii) in maturation medium for forebrain organoids, for the indicated time until fixation (Fig 1A), with medium changes every 3 days.

### BrdU labeling of cerebral organoids

To label cerebral organoid cells in S‐Phase, a 1 h BrdU pulse was applied 47 h after electroporation by replacing the culture medium (differentiation medium containing vitamin A) with culture medium containing in addition 15 μM BrdU. Cerebral organoids were fixed 1 h later (see below).

### Fixation and cryosectioning of brain organoids

Brain organoids were fixed at the indicated time points (see also Fig 1A) in 4% paraformaldehyde in 120 mM phosphate buffer pH 7.4 for 2 h at 4°C. Fixed brain organoids were sequentially incubated in phosphate‐buffer‐containing 15% sucrose and then 30% sucrose, each time overnight, at 4°C, embedded in Tissue‐Tek OCT (Sakura), and frozen on dry ice. Cryosections of 20 μm thickness were cut and stored at −20°C until further use.

### Immunohistochemistry

Immunohistochemistry was performed as previously described (Mora‐Bermudez *et al*, 2016; Namba *et al*, 2020). The following primary antibodies were used: ARHGAP11B (mouse monoclonal, MPI‐CBG (Namba *et al*, 2020), 1:200), BrdU (mouse monoclonal, EXBIO, 11‐286‐C100, RRID:AB_10732986, 1:300), Ctip2 (rat monoclonal, Abcam, ab18465, RRID:AB_2064130, 1:500), FOXG1 (rabbit polyclonal, Abcam, ab18259, RRID:AB_732415, 1:300), GFP (chicken polyclonal, Aves Labs, GFP‐1020, RRID:AB_10000240, 1:500), HOPX (rabbit polyclonal, Sigma‐Aldrich, HPA030180, RRID:AB_10603770, 1:300) Hu (mouse monoclonal, Thermo Fisher, A‐21271, RRID:AB_221488, 1:200), Ki67 (mouse monoclonal, Agilent, M7249, RRID:AB_2250503, 1:300), NeuN (rabbit polyclonal, Abcam, ab104225, RRID:AB_10711153, 1:300), PCNA (mouse monoclonal, Millipore, CBL407, RRID:AB_93501, 1:300), PTPRZ1 (rabbit polyclonal, Atlas Antibodies, HPA015103, RRID:AB_1855946, 1:500), SATB2 (mouse monoclonal, Abcam, ab51502, RRID: AB_882455, 1:300) Sox2 (goat polyclonal, R + D Systems, AF2018, RRID:AB_355110, 1:150), Tbr2 (rabbit polyclonal, Abcam, ab23345, RRID:AB_778267, 1:500).

For all immunostainings on brain organoids, antigen retrieval was performed in 0.01 M sodium citrate buffer (pH 6.0) for 1 h at 70°C, prior to the overnight incubation with primary antibodies. The following secondary antibodies were used at a concentration of 1:500: Cy2: anti‐chicken (donkey polyclonal, Dianova, 703–225‐155, RRID:AB_2340370); Alexa Fluor 488: anti‐chicken (goat polyclonal, Thermo Fisher, A‐11039, RRID:AB_142924); Alexa Fluor 555: anti‐goat (donkey polyclonal, Thermo Fisher, A‐21432, RRID:AB_141788), anti‐mouse (donkey polyclonal, Thermo Fisher, A‐31570, RRID:AB_2536180), anti‐rabbit (donkey polyclonal, Thermo Fisher, A‐31572, RRID:AB_162543), anti‐rat (goat polyclonal, Thermo Fisher, A‐21434, RRID:AB_2535855); Alexa Fluor 594: anti‐goat (donkey polyclonal, Thermo Fisher, A‐11058, RRID:AB_2534105), anti‐mouse (donkey polyclonal, Thermo Fisher, A‐21203, RRID:AB_141633), anti‐rabbit (donkey polyclonal, Thermo Fisher, A‐21207, RRID:AB_141637); Alexa Fluor 647: anti‐mouse (donkey polyclonal, Thermo Fisher, A‐31571, RRID:AB_162542), anti‐rabbit (donkey polyclonal, Thermo Fisher, A‐31573, RRID:AB_2536183). All immunostained cryosections were counterstained with DAPI.

### Image acquisition

Images were acquired using a Zeiss LSM 880 with 10×, 20×, and 40× objectives, a Zeiss LSM 800 with a 20× objective, a Zeiss LSM 780 NLO microscope with 10× and 20× objectives, or a Leica DM6 B microscope with a 20× objective. For the Zeiss microscopes, images were taken as stacks of five 1‐μm optical sections. When images were taken as tile scans, they were stitched together using the Zeiss ZEN software.

### Quantifications

All quantifications were performed blindly. Primary data were processed and results plotted using Prism (GraphPad Software), R software version 4.0.2 (R Development Core Team, 2020), and SPSS Statistics (IBM). For all quantifications, brain organoids from at least two independent batches were typically used. For comparative analyses, ventricle‐like structures were quantified, which had similar morphology with regard to the VZ (packing density of nuclei, radial organization), SVZ, and NL, and exhibited a sufficiently high and roughly equal number of electroporated cells. The borders between VZ and SVZ/NL were defined on the basis of radial organization and density of nuclei. Cell counts were performed in Fiji or Imaris. The mean of several electroporated ventricle‐like structures was calculated. The data obtained from the quantifications of electroporated samples are expressed as a proportion of the GFP‐positive cell population. Data obtained from non‐electroporated samples are expressed per area.

### Statistical analysis

All statistical analyses were conducted using Prism (GraphPad Software), R software version 4.0.2 (R Development Core Team, 2020), and SPSS Statistics (IBM). Sample sizes (number of organoids per condition) are indicated in the figure legends. Data were analyzed for normal distribution using the Shapiro–Wilk test. If the criteria for normal distribution were fulfilled, statistical significance was tested using the two‐sided Student's *t*‐test. If the criteria for normal distribution were not fulfilled, statistical significance was tested using the one‐sided Wilcoxon rank sum test. Moreover, for experiments including more than two groups, one‐way analysis of variance (ANOVA) followed by Bonferroni's multiple comparison test was applied, if the criteria for normal distribution were fulfilled, and the Kruskal–Wallis test was applied, if the criteria for normal distribution were not fulfilled. Statistical significances are indicated in the figure legends.

## Author contributions

**Jan Fischer:** Conceptualization; formal analysis; investigation; writing – original draft. **Eduardo Fernández Ortuño:** Formal analysis; investigation. **Fabio Marsoner:** Conceptualization; formal analysis; investigation; writing – original draft; writing – review and editing. **Annasara Artioli:** Formal analysis; investigation. **Jula Peters:** Investigation. **Takashi Namba:** Resources. **Christina Eugster Oegema:** Investigation. **Wieland B Huttner:** Conceptualization; supervision; funding acquisition; writing – original draft; writing – review and editing. **Julia Ladewig:** Conceptualization; supervision; funding acquisition; writing – original draft; writing – review and editing. **Michael Heide:** Conceptualization; formal analysis; supervision; funding acquisition; investigation; writing – original draft; writing – review and editing.

## Disclosure and competing interest statement

The authors declare that they have no conflict of interest.

## Supporting information



Source Data for Expanded View and Appendix

Appendix

Expanded View Figures PDF

Dataset EV1

Dataset EV2

Dataset EV3

PDF+

Source Data for Figure 4

## Data Availability

The DNA sequencing data produced in this study are available in the European Nucleotide Archive PRJEB53539 (https://www.ebi.ac.uk/ena/browser/view/PRJEB53539).
