# Supplementary material for: Human‐specific ARHGAP11B ensures human‐like basal progenitor levels in hominid cerebral organoids
Source: EMBO Rep. 2022 Sep 13;23(11):e54728. doi: 10.15252/embr.202254728 (PMC9646322; doi:10.15252/embr.202254728)
Supplement: Supplementary file 2 — Appendix [file EMBR-23-e54728-s008.pdf]

Appendix

Table of contents

Appendix Materials and Methods	page 2
Appendix Figure S1	page 4
Appendix Figure S2	page 6
Appendix Figure S3	page 8
Appendix Figure S4	page 10
Appendix Figure S5	page 11

Appendix Materials and Methods

Whole Genome Sequencing

Genomic DNA was isolated using the Quick-DNA Miniprep Plus Kit (ZYMO Research) and eluted in low TE buffer (10 mM Tris-HCl, 0.1 mM EDTA pH 8.5). DNA concentration was determined by NanoDrop. For DNA library generation, 1 µg of DNA was sheared using Covaris shearing (400 bp) and libraries were constructed using the KAPA HyperPlus Kit (KAPABIOSYSTEMS). Libraries were sequenced as paired-end 150 bp reads to a target of 50x coverage on an Illumina NovaSeq 6000.

Bioinformatics analysis for off-target integration

Whole Genome DNA-Sequencing 2x150 bp paired-end reads (clone j (sample a23): 441 Mio pairs, B7_028#4 iPSC line (sample s28): 670 Mio, clone 16 (sample a40): 492 Mio and GM08680 iPSC line (sample s80): 1011 Mio) were trimmed for the Illumina Universal adapter sequence AGATCGGAAGAG with an error probability of 5% and discarding all trimmed reads with length shorter than 25 nt with the help of cutadapt v1.16. The resulting trimmed paired-end reads have been referred to as trimmed read sets 1. A copy of reads1 and reads2 were then split from each other and combined into read sets treated as single-end reads. Cutadapt was applied a second time to these constructed single-end reads trimming off from the 3' read ends the 5'-sequence of the CRISPR-Cas9 insert (GTACCATCGAGCTAG) and its reverse complement (CTAGCTCGATGGTAC) with an error probability of 5% and ensuring a minimum overlap of 9 nt. Only the 5'-sequence of the insert was known to us. All untrimmed reads were discarded while trimmed reads were kept and will be referred to as read sets 2. These read sets 2 are supposed to

contain those reads that overlap the 5'end of the insert. Read sets 1 were mapped to the human reference genome sequence GRCh38 Ensembl v99 with BWA v0.7.17-r1198-dirty. Read sets 2 were mapped identically and further processed with custom in-house scripts to detect clusters of overlapping reads per sample. Each cluster of reads defines a genomic region by the minimal and maximal genome position of mapped reads. The number of reads mapping to each region in the CRISRP-Cas9 samples and the corresponding WT samples were determined and output in plain table format (Datasets EV2 and EV3). Fold changes of the read numbers (CRISPR-Cas9 vs WT), read Ids, strand information were added to this tables (Datasets EV2 and EV3) as well as information (distance, sequence, gene annotation) on the closest genome-wide match (GRCh38) of any of the three guide sequences + PAM sequence as determined with the online tool wge.stemcell.sanger.ac.uk/find_off_targets_by_seq on May 2nd, 2022.

Genomic regions with 3 or more CRISPR-Cas9 reads (NREADS_CRISPR) were considered as potential integrations and were manually inspected for gene annotations. Potential integrations in exonic regions were summarized in Dataset EV1.

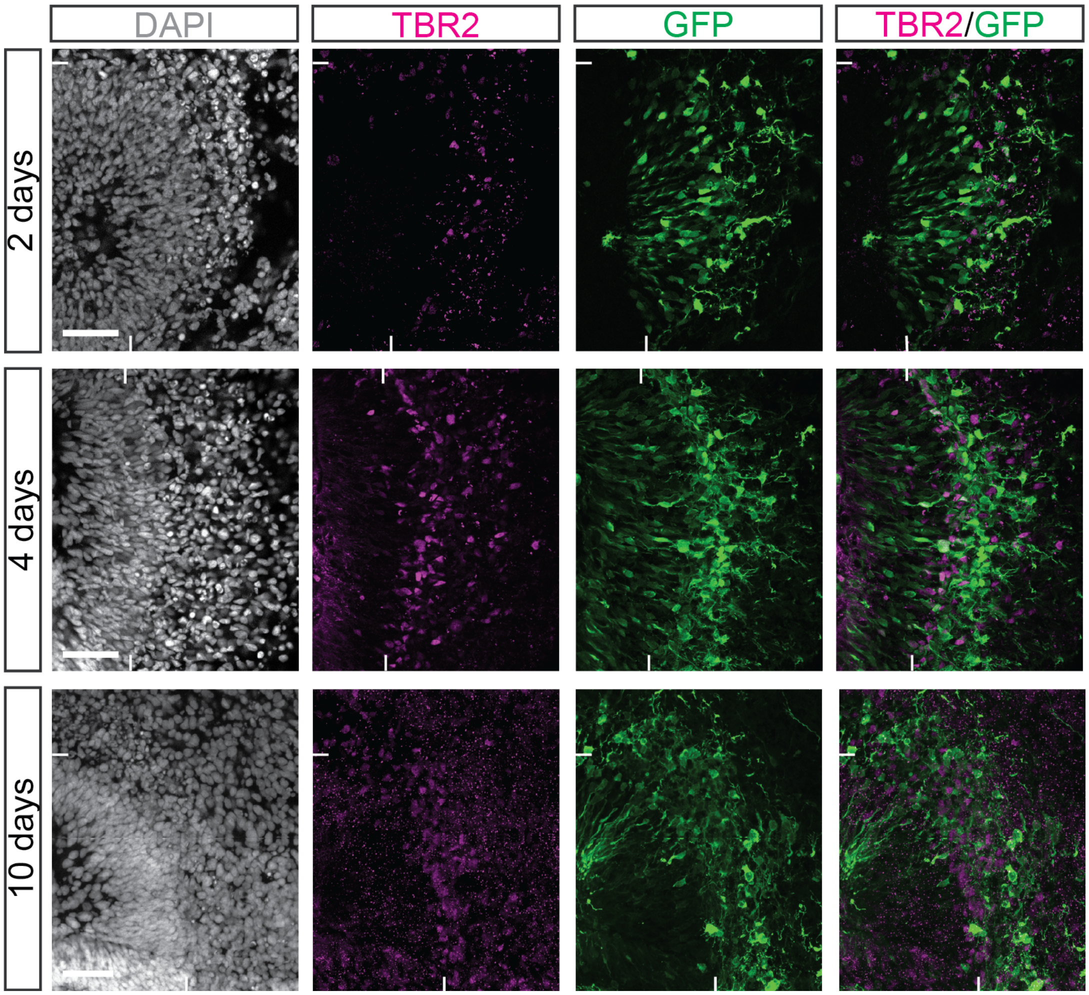

Appendix Figure S1 - Localization of TBR2-positive and GFP-positive cells in electroporated chimpanzee cerebral organoids.

Double immunofluorescence for TBR2 (magenta) and GFP (green), combined with DAPI staining (white), of a 57 days-old chimpanzee cerebral organoid 2 days after electroporation with GFP expression plasmid plus control plasmid (top row), of a 59 days-old chimpanzee cerebral organoid 4 days after electroporation with GFP expression plasmid plus control plasmid (middle row) and a 61 days-old chimpanzee cerebral organoid 10 days after electroporation with GFP expression plasmid plus control plasmid (bottom row). Tick marks indicate the border between VZ and

SVZ/NL. Note that for 10 days after electroporation the same electroporated region is depicted in Appendix Figure S2 with a different marker (CTIP2). Scale bars, 50 µm.

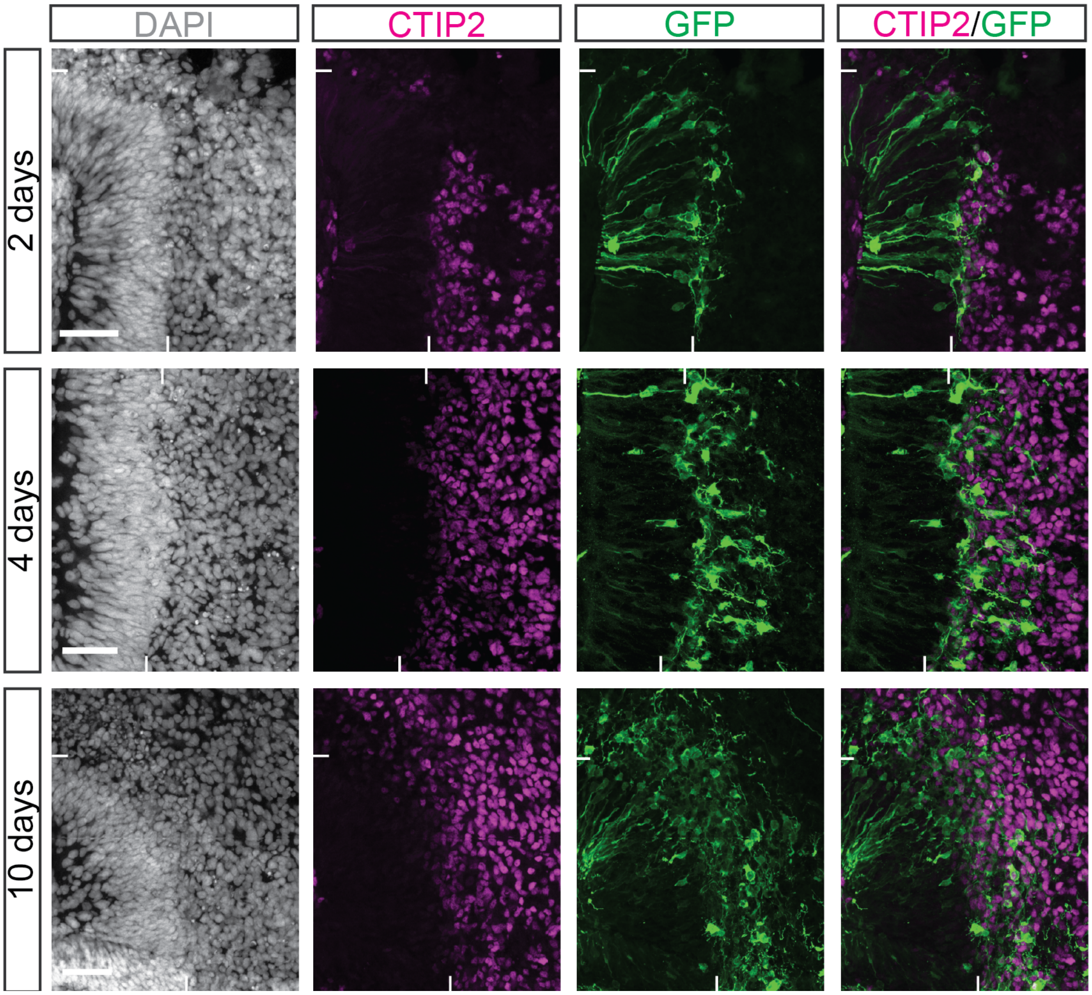

Appendix Figure S2 - Localization of CTIP2-positive and GFP-positive cells in electroporated chimpanzee cerebral organoids.

Double immunofluorescence for CTIP2 (magenta) and GFP (green), combined with DAPI staining (white), of a 57 days-old chimpanzee cerebral organoid 2 days after electroporation with GFP expression plasmid plus control plasmid (top row), of a 59 days-old chimpanzee cerebral organoid 4 days after electroporation with GFP expression plasmid plus control plasmid (middle row) and a 61 days-old chimpanzee cerebral organoid 10 days after electroporation with GFP expression plasmid plus control plasmid (bottom row). Tick marks indicate the border between VZ and SVZ/NL. Note that for 2 and 4 days after electroporation the same electroporated regions are

depicted in Appendix Figure S3 with a different marker (SATB2) and that for 10 days after electroporation the same electroporated region is depicted in Appendix Figure S1 with a different marker (TBR2). Scale bars, 50 μm.

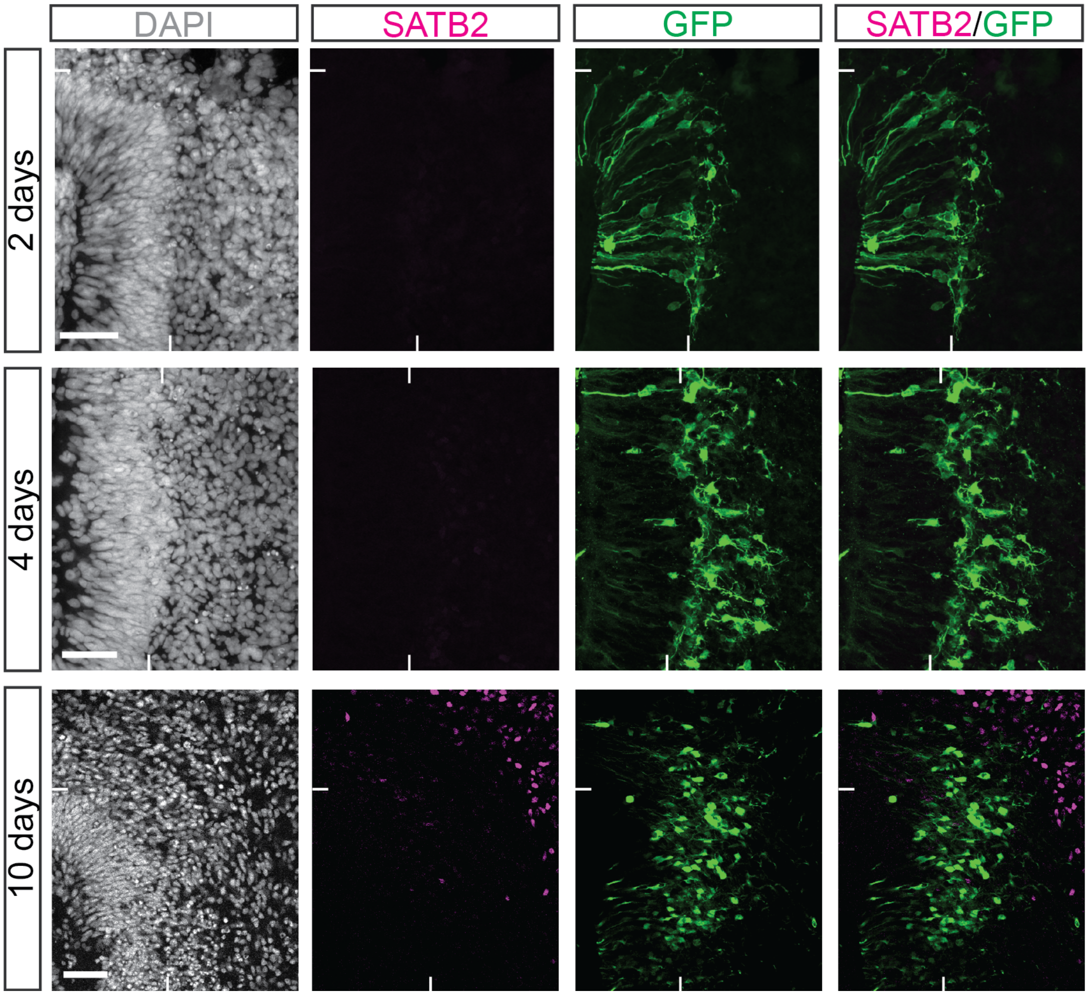

Appendix Figure S3 - Localization of SATB2-positive and GFP-positive cells in electroporated chimpanzee cerebral organoids.

Double immunofluorescence for SATB2 (magenta) and GFP (green), combined with DAPI staining (white), of a 57 days-old chimpanzee cerebral organoid 2 days after electroporation with GFP expression plasmid plus control plasmid (top row), of a 59 days-old chimpanzee cerebral organoid 4 days after electroporation with GFP expression plasmid plus control plasmid (middle row) and a 61 days-old chimpanzee cerebral organoid 10 days after electroporation with GFP expression plasmid plus control plasmid (bottom row). Tick marks indicate the border between

VZ and SVZ/NL. Note that for 2 and 4 days after electroporation the same electroporated regions are depicted in Appendix Figure S2 with a different marker (CTIP2). Scale bars, 50 µm.

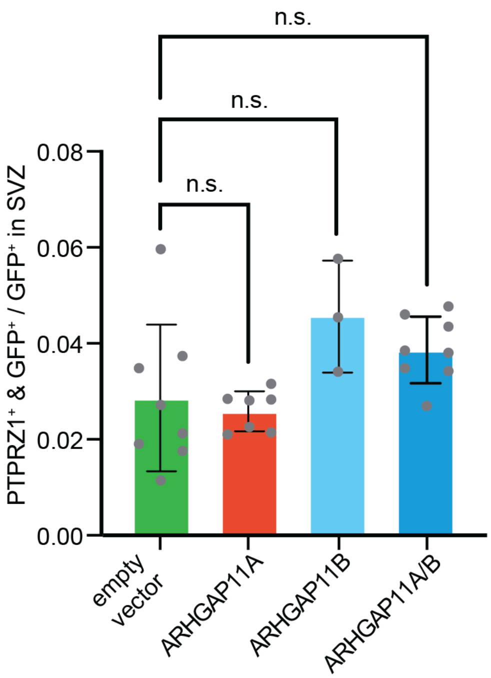

Appendix Figure S4 - Overexpression of *ARHGAP11A, ARHGAP11B* or a combination of *ARHGAP11A* plus *ARHGAP11B* in human WT forebrain organoids does not significantly increase bRG abundance.

Quantification of the proportion of GFP+ cells in the SVZ that are PTPRZ1+ in 60 days-old WT human forebrain organoids 10 days after electroporation with GFP plus either control plasmid only (empty vector, green), *ARHGAP11A* expression plasmid (ARHGAP11A, red), *ARHGAP11B* expression plasmid only (ARHGAP11B, light blue), or *ARHGAP11A* plus *ARHGAP11B* expression plasmids (ARHGAP11A/B, dark blue). Data are the mean of 8 control, 7 *ARHGAP11A*-, 3 *ARHGAP11B*- and 8 *ARHGAP11A* plus *ARHGAP11B*-transfected human forebrain organoids of two independent batches each; error bars indicate SD; n.s., not significant (one-way ANOVA with Bonferroni's multiple comparisons test).

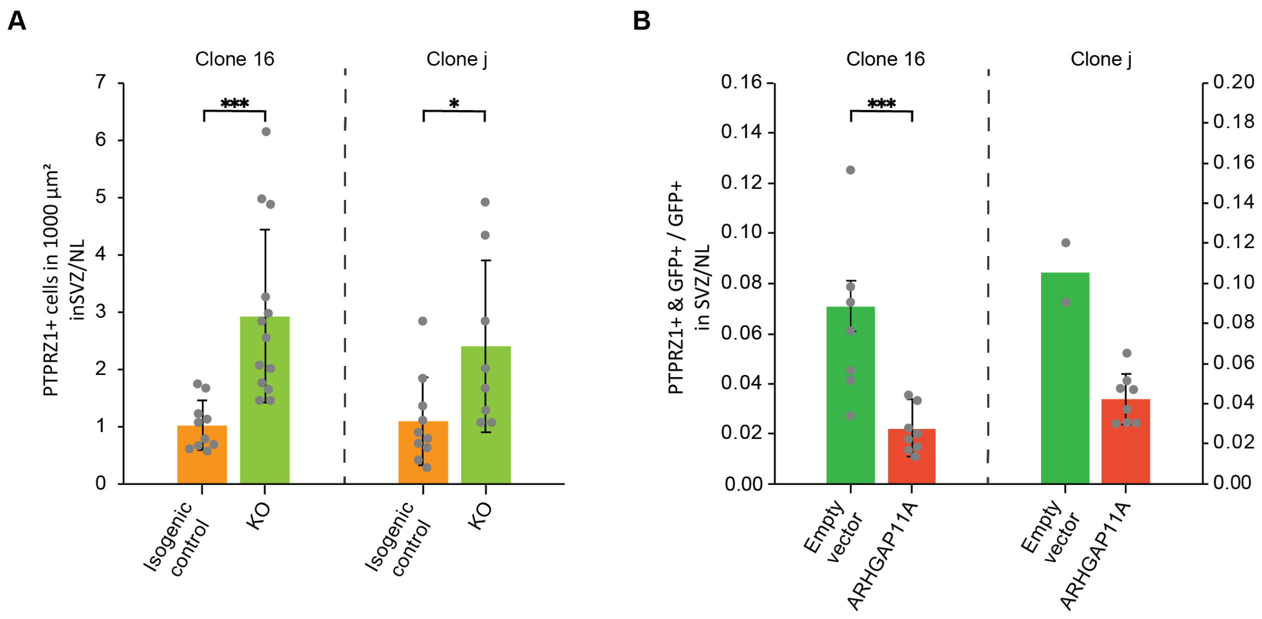

Figure S5 - Comparison of bRG levels between *ARHGAP11A* plus *ARHGAP11B* double-knockout human forebrain organoids and organoids generated from control iPSCs, and the role of ARHGAP11A.

A Quantification of PTPRZ1+ cells in a 1000 µm^2 large area in the SVZ/NL of 55 days-old isogenic control and *ARHGAP11A* plus *ARHGAP11B* double-knockout human forebrain organoids. Organoids were generated from isogenic control iPSCs to clone16 (orange) and clone 16 iPSCs (light green) (left bars), and isogenic control iPSCs to clone j (orange) and clone j iPSCs (light green) (right bars). Data are the mean of 10 isogenic control to clone 16, 13 clone 16, 10 isogenic control to clone j, and 8 clone j human forebrain organoids of two independent batches each; error bars indicate SD; *, P <0.05; ***, P <0.001 (two-sided Student's *t*-test).

Interpretation: Despite the lack of ARHGAP11B expression in the *ARHGAP11A* plus *ARHGAP11B* double-knockout human forebrain organoids, there is an increase in bRG levels in these organoids as compared to isogenic control organoids. This increase is thought to reflect an increased cNPC delamination due to the lack of ARHGAP11A action in the VZ. Increased cNPC delamination will lead to increased bRG levels. Support for this notion is provided in panel B.

B Quantification of the proportion of GFP+ cells in the SVZ/NL that are PTPRZ1+ in 60 days-old *ARHGAP11A* plus *ARHGAP11B* double-knockout human forebrain organoids. Organoids were generated from clone 16 (left) and clone j (right), and data were obtained 10 days after electroporation with GFP plus either empty vector (left bars of each pair, dark green) or *ARHGAP11A* expression plasmid (ARHGAP11A, right bars of each pair; dark red). For clone 16, data are the mean of 7 empty vector and 8 *ARHGAP11A* -transfected *ARHGAP11A* plus *ARHGAP11B* double-knockout human forebrain organoids of two independent batches each; for clone j, data are the mean of 2 empty vector and 8 *ARHGAP11A* -transfected *ARHGAP11A* plus *ARHGAP11B* double-knockout human forebrain organoids of one-three independent batches; error bars indicate SD; ***, P <0.001 (one-sided Wilcoxon rank sum test).

The data for ARHGAP11A in panel B are the same as in Fig 6.

Interpretation: When ARHGAP11A action in the *ARHGAP11A* plus *ARHGAP11B* double-knockout human forebrain organoids is restored upon electroporation of the *ARHGAP11A* expression plasmid, bRG levels are back down to normal. As ARHGAP11B action in the *ARHGAP11A* plus *ARHGAP11B* double-knockout human forebrain organoids is still lacking, bRG levels remain low.
