# Supplementary material for: Human‐specific ARHGAP11B ensures human‐like basal progenitor levels in hominid cerebral organoids
Source: EMBO Rep. 2022 Sep 13;23(11):e54728. doi: 10.15252/embr.202254728 (PMC9646322; doi:10.15252/embr.202254728)
Supplement: Supplementary file 3 — Expanded View Figures PDF [file EMBR-23-e54728-s004.pdf]

Expanded View Figures

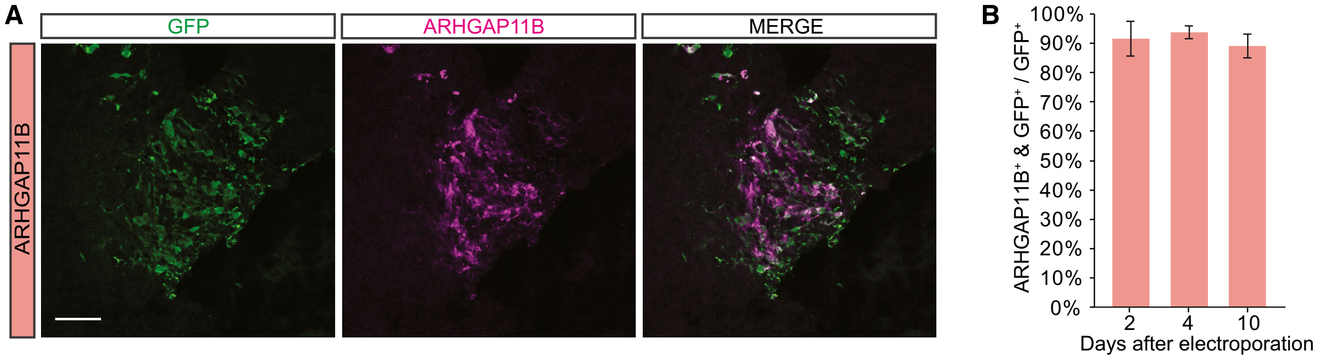

Figure EV1. GFP and *ARHGAP11B* are co-expressed when co-electroporated in chimpanzee cerebral organoids.

A Double immunofluorescence for GFP (green) and ARHGAP11B (magenta) of a 59-day-old chimpanzee cerebral organoid 4 days after electroporation with GFP expression plasmid plus *ARHGAP11B* expression plasmid. Note that GFP and ARHGAP11B immunofluorescence signals do not completely overlap, as GFP is localized in the cytoplasm, whereas ARHGAP11B is localized in mitochondria. Scale bar, 50 µm.

B Quantification of the percentage of GFP+ cells that are ARHGAP11B+ in 57-, 59-, and 61-day-old chimpanzee cerebral organoids 2, 4, and 10 days after electroporation with GFP expression plasmid plus *ARHGAP11B* expression plasmid. Data are the mean of five, six, or seven ventricle-like structures 2, 4, or 10 days after electroporation of three *ARHGAP11B*-transfected cerebral organoids each; error bars indicate SD.

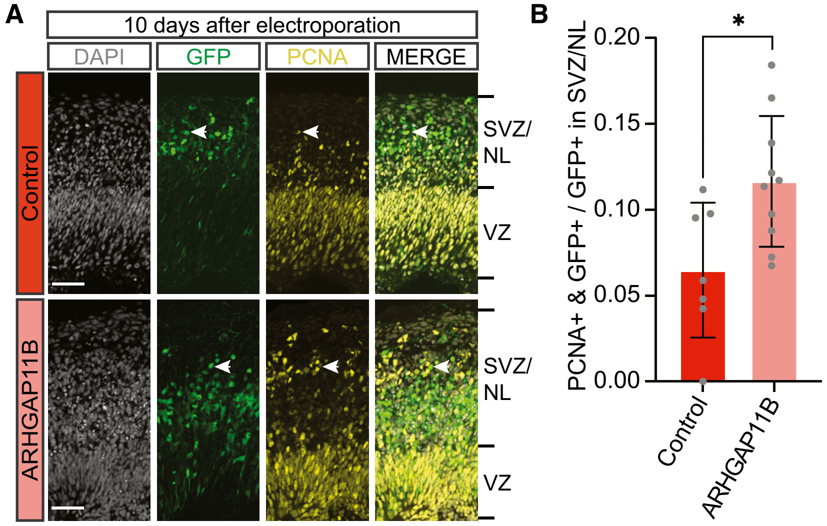

Figure EV2. Expression of *ARHGAP11B* in chimpanzee cerebral organoids increases the abundance of PCNA-positive cells in the SVZ.

A Double immunofluorescence for GFP (green) and the cycling cell marker PCNA (yellow), combined with DAPI staining (white), of a 61-day-old chimpanzee cerebral organoids 10 days after electroporation with GFP expression plasmid plus either control plasmid (top) or *ARHGAP11B* expression plasmid (bottom). Tick marks indicate the borders of the VZ and SVZ/NL; arrowheads indicate examples of GFP+ and PCNA+ double-positive cells. Note that the same electroporated regions are depicted in Fig 4 with a different marker (Hu). Scale bars, 50 µm.

B Quantification of the proportion of GFP+ cells in the SVZ/NL that are PCNA+ in 61-day-old chimpanzee cerebral organoids 10 days after electroporation with GFP expression plasmid plus either control plasmid (dark red) or *ARHGAP11B* expression plasmid (light red). Data are the mean of seven control and 10 *ARHGAP11B*-transfected cerebral organoids of two independent batches each; error bars indicate SD; $^{*}P < 0.05$ (two-sided Student's t-test).

Source data are available online for this figure.

 © 2022 The Authors

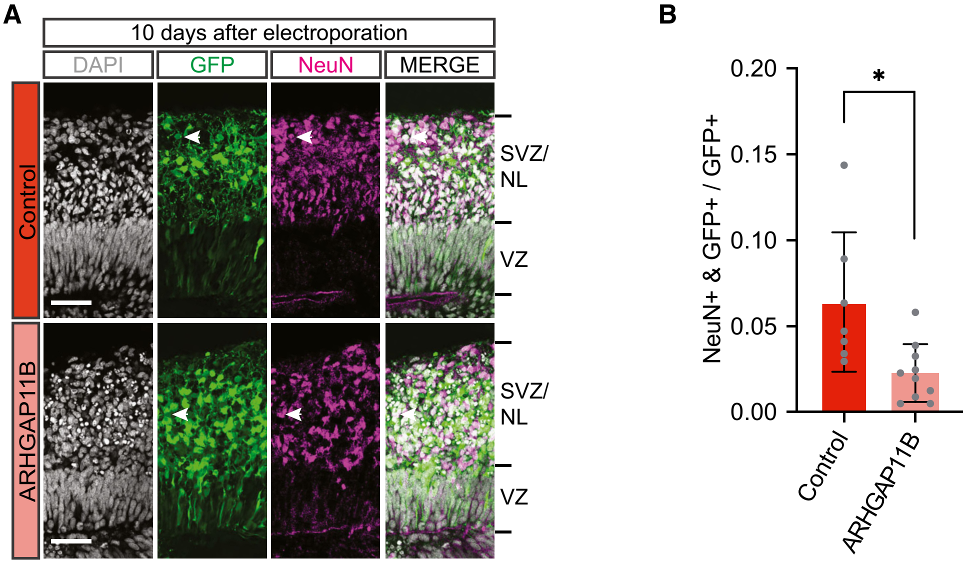

Figure EV3. Expression of *ARHGAP11B* in chimpanzee cerebral organoids decreases the abundance of NeuN-positive cells.

A Double immunofluorescence for GFP (green) and the neuron marker NeuN (magenta), combined with DAPI staining (white), of a 61-day-old chimpanzee cerebral organoid 10 days after electroporation with GFP expression plasmid plus either control plasmid (top) or *ARHGAP11B* expression plasmid (bottom). Tick marks indicate the borders of the VZ and SVZ/NL; arrowheads indicate examples of GFP+ and NeuN+ double-positive cells. Scale bars, 50 µm.

B Quantification of the proportion of GFP+ cells that are NeuN+ in 61-day-old chimpanzee cerebral organoids 10 days after electroporation with GFP expression plasmid plus either control plasmid (dark red) or *ARHGAP11B* expression plasmid (light red). Data are the mean of seven control and 10 *ARHGAP11B*-transfected cerebral organoids of two independent batches each; error bars indicate SD; *$P < 0.05$ (two-sided Student's t-test).

© 2022 The Authors

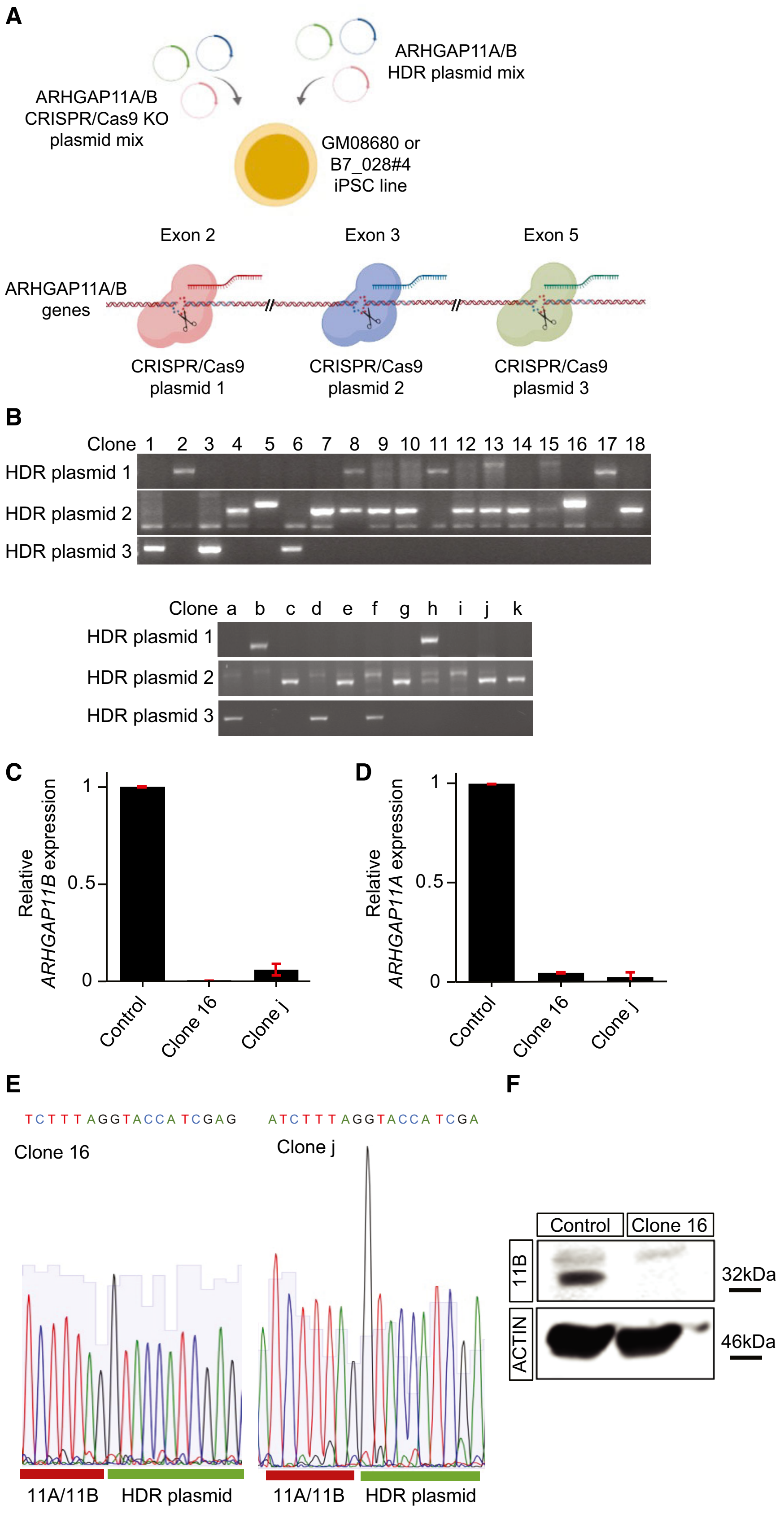

Figure EV4. Validation of the human *ARHGAP11A* plus *ARHGAP11B* double-knockout iPSC clones 16 and j.

A Schematic overview of the knockout strategy (image created with BioRender.com). A mixture of three CRISPR/Cas9 plasmids, each encoding the gRNA targeting either exon 2, 3, or 5 and SpCas9 ribonuclease, and three Homology-Directed Repair (HDR) plasmids, each containing the corresponding homology arms and encoding a puromycin resistance cassette under the control of the EF-1α promoter, were nucleofected into human GM08680 and B7_028#4 iPSCs. Due to the targeting by the CRISPR/Cas9 plasmids of the sites in exon 2, 3, and 5 of the *ARHGAP11A* and/or *ARHGAP11B* genes, each of these sites should show integration of the corresponding HDR plasmid.

B Genomic PCR validation of the indicated human iPSC clones for integration of the HDR plasmid. PCR primers were designed to recognize the integration of the HDR plasmid in exon 2 (HDR plasmid 1), exon 3 (HDR plasmid 2), or exon 5 (HDR plasmid 3) of *ARHGAP11B*. The image shows 18 of the 24 clones analyzed for the GM08680 iPSC line (clone 1–18) and 11 of the 14 clones analyzed for the B7_028#4 iPSC line (clone a-k).

C, D Analysis of the relative expression levels of the *ARHGAP11B* (C) and *ARHGAP11A* (D) mRNAs by q-RT-PCR of control, clone 16, and clone j iPSCs, showing virtually complete lack of expression for both genes in clone 16 and clone j, indicative of a homozygous *ARHGAP11A* plus *ARHGAP11B* double-knockout. Data are the mean of three independent experiments; error bars indicate SD.

E Sequencing analysis of clone 16 and clone j iPSCs confirmed the disruption of the *ARHGAP11A* plus *ARHGAP11B* genes in exon 3 in both clones due to the integration of the HDR plasmid, indicative of a double-knockout.

F Immunoblot for ARHGAP11B (11B, top) and actin (bottom) of cleared lysates from control (left) and clone 16 (right) iPSCs.

© 2022 The Authors
