# Supplementary figures and images for: Human‐specific ARHGAP11B ensures human‐like basal progenitor levels in hominid cerebral organoids
Source: EMBO Rep. 2022 Sep 13;23(11):e54728. doi: 10.15252/embr.202254728 (PMC9646322; doi:10.15252/embr.202254728)

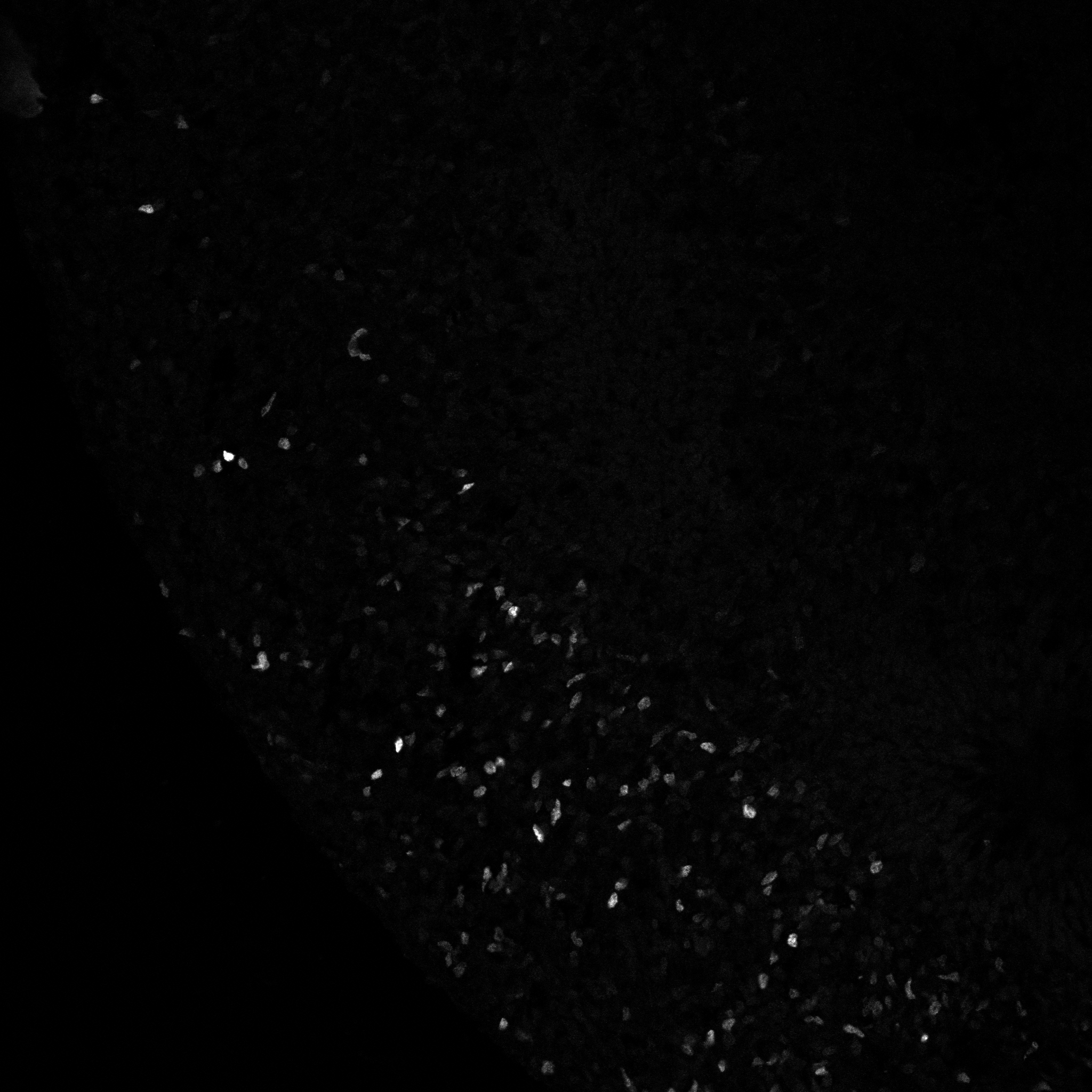

Supplement: Supplementary file 8 — Source Data for Figure 4 [file EMBR-23-e54728-s005.zip › Figure 3/3E/ARHGAP11B_15days_SATB2.tif]

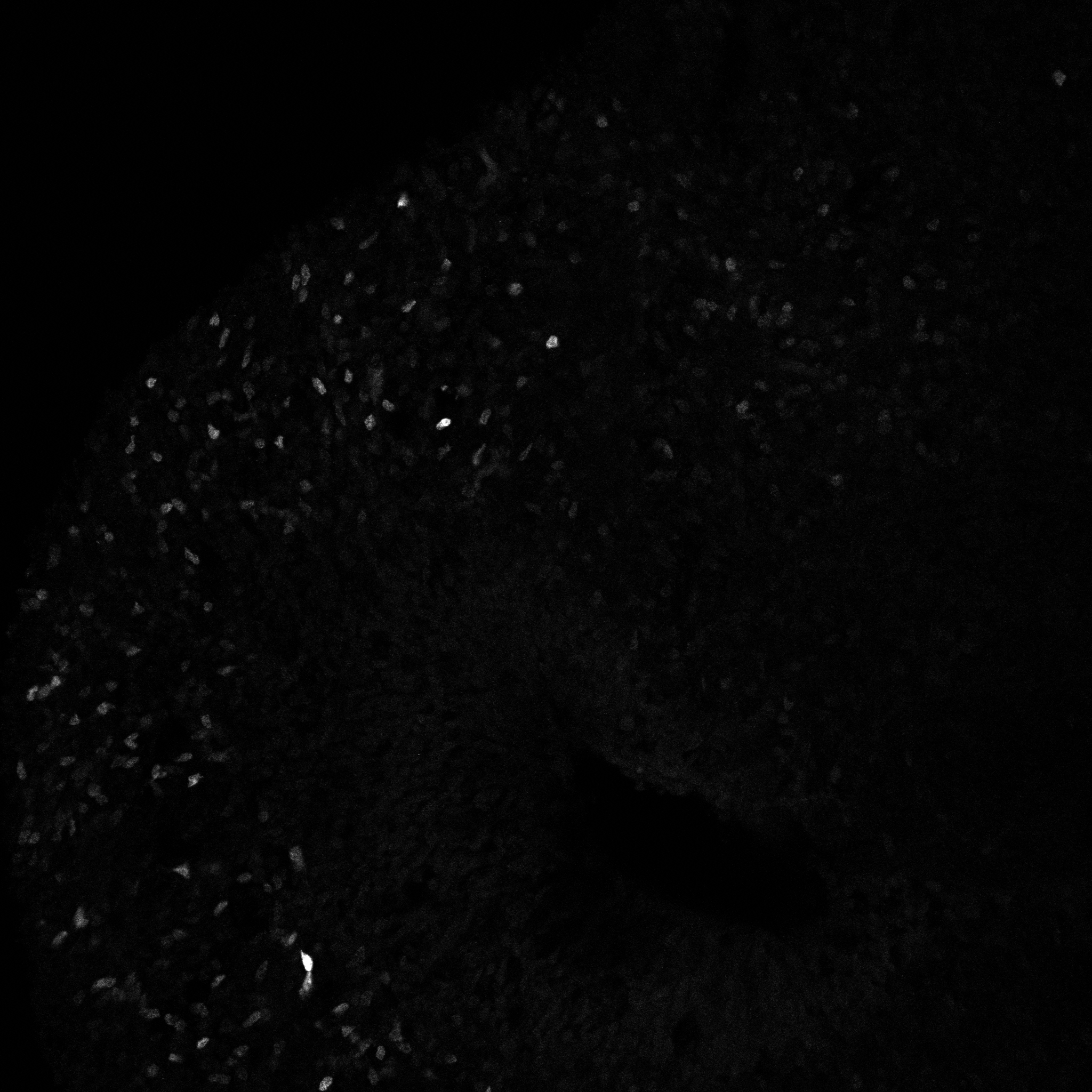

Supplement: Supplementary file 8 — Source Data for Figure 4 [file EMBR-23-e54728-s005.zip › Figure 3/3E/Control_15days_SATB2.tif]

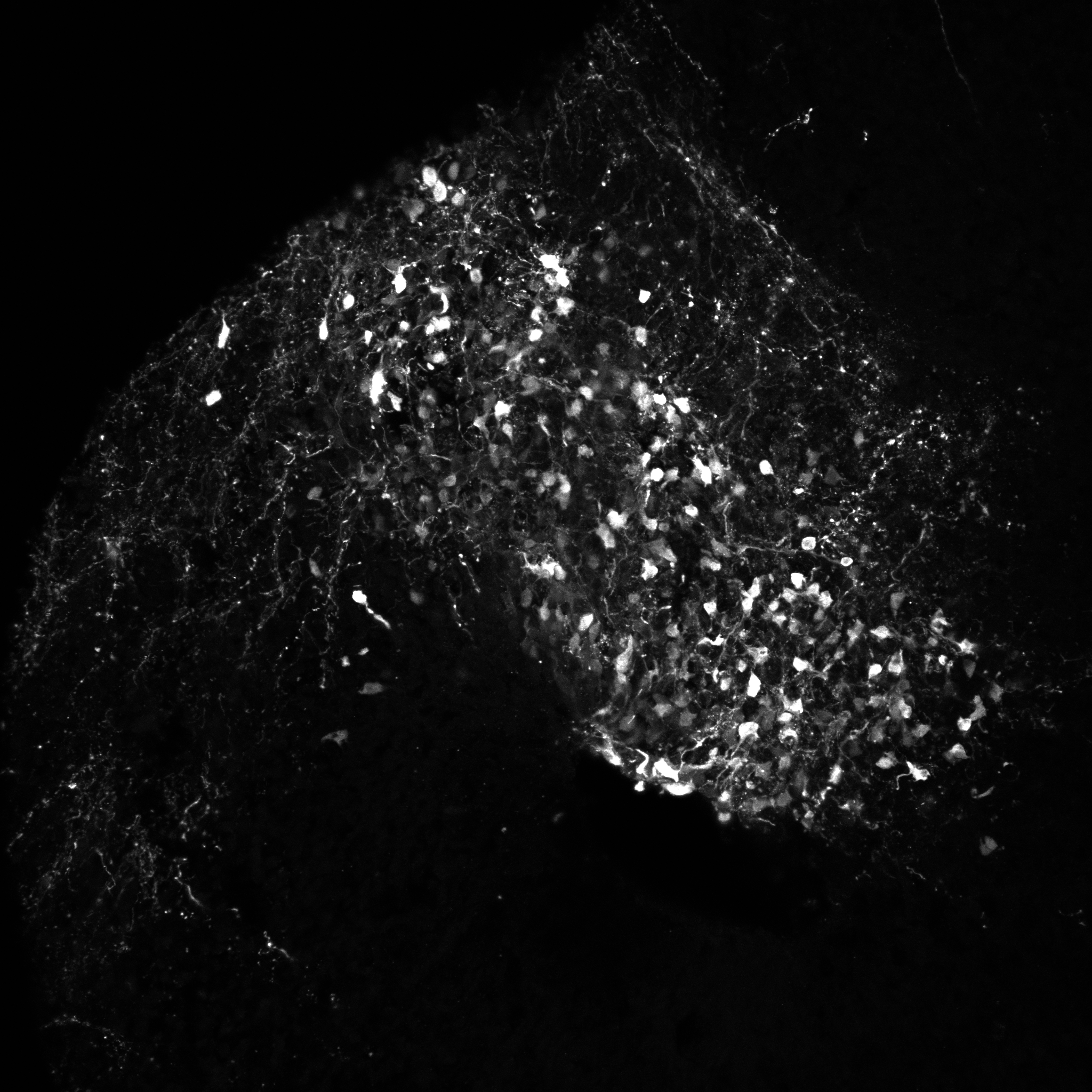

Supplement: Supplementary file 8 — Source Data for Figure 4 [file EMBR-23-e54728-s005.zip › Figure 3/3E/Control_15days_GFP.tif]

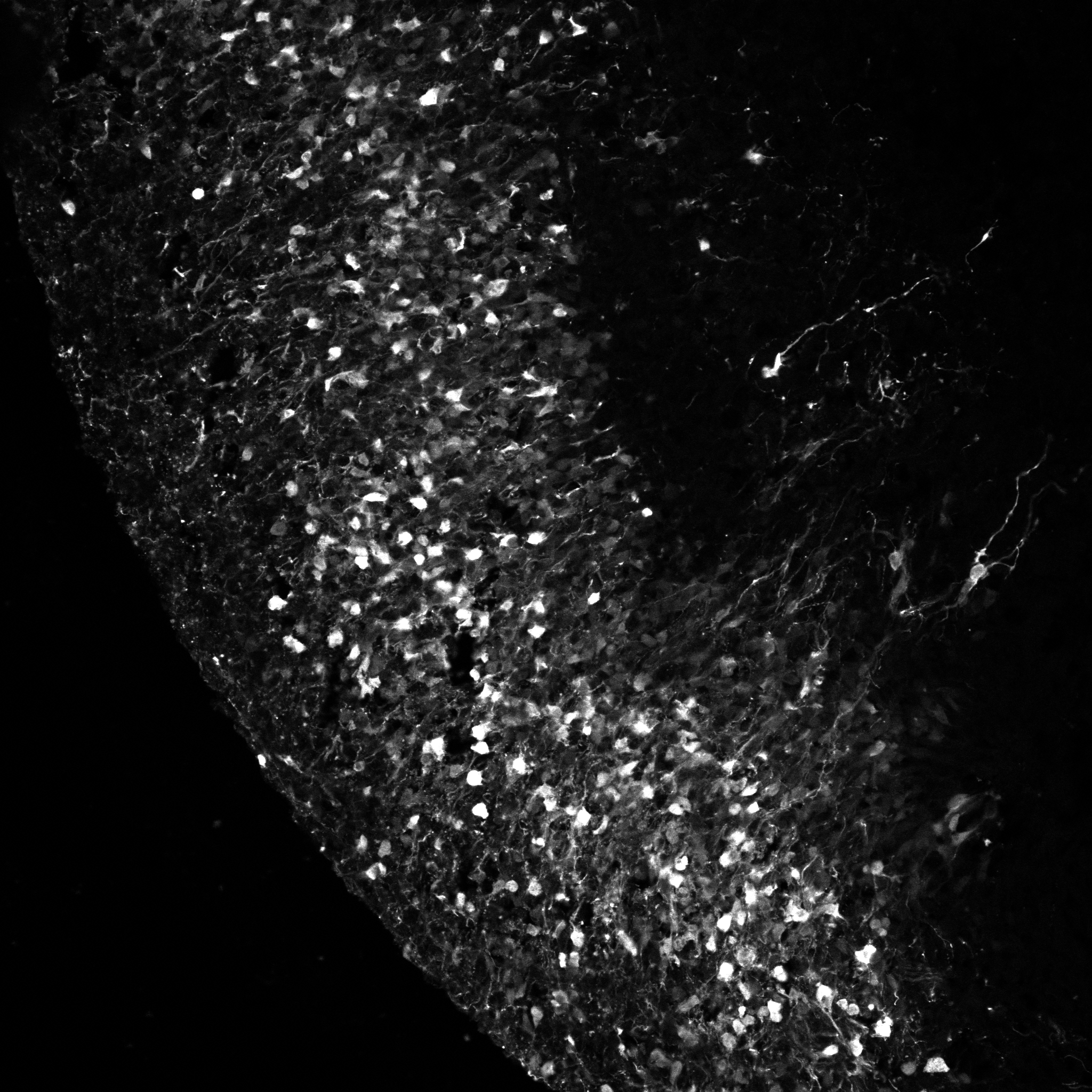

Supplement: Supplementary file 8 — Source Data for Figure 4 [file EMBR-23-e54728-s005.zip › Figure 3/3E/ARHGAP11B_15days_GFP.tif]

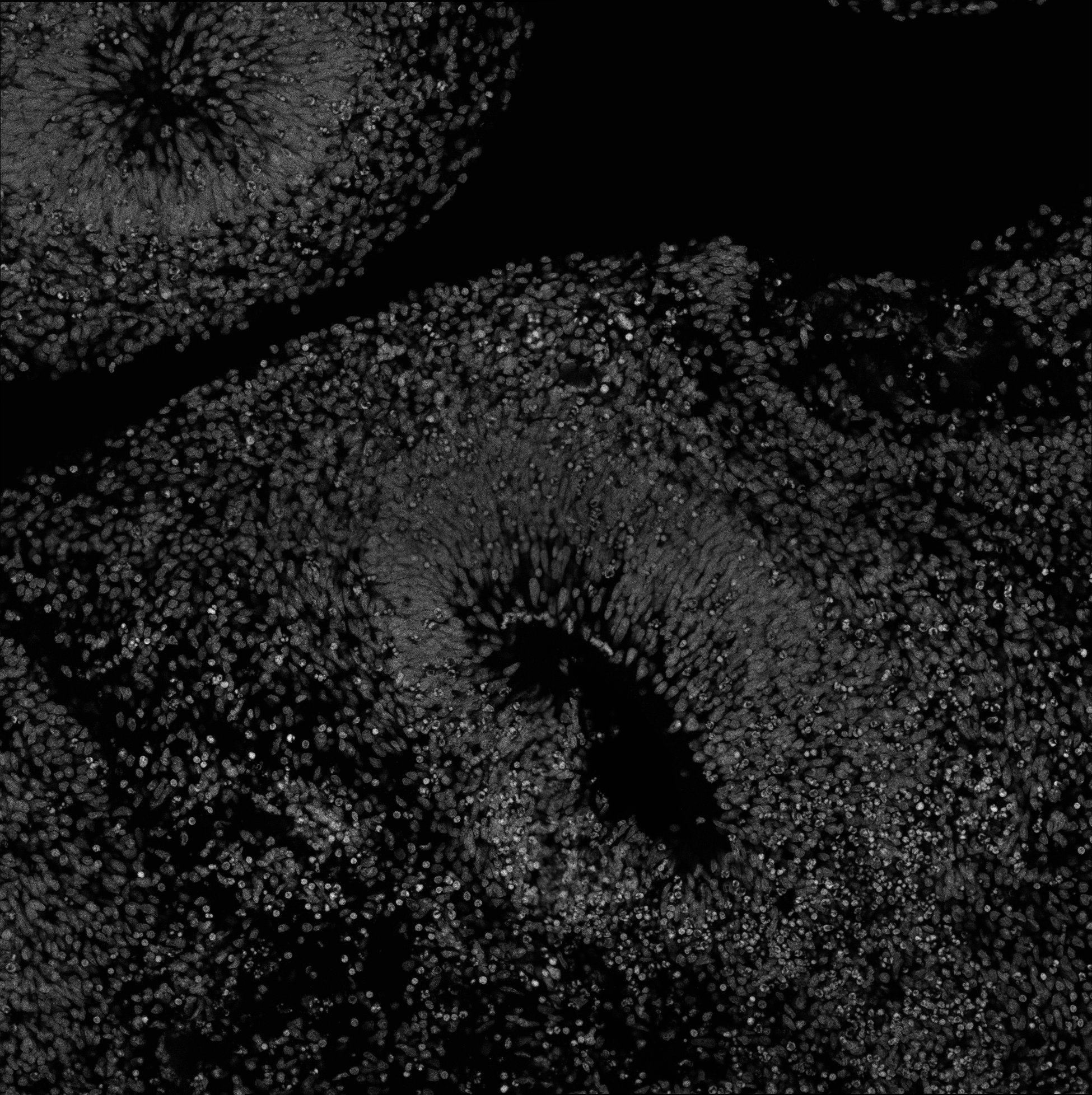

Supplement: Supplementary file 8 — Source Data for Figure 4 [file EMBR-23-e54728-s005.zip › Figure 3/3C/ARHGAP11B_4days_DAPI.tif]

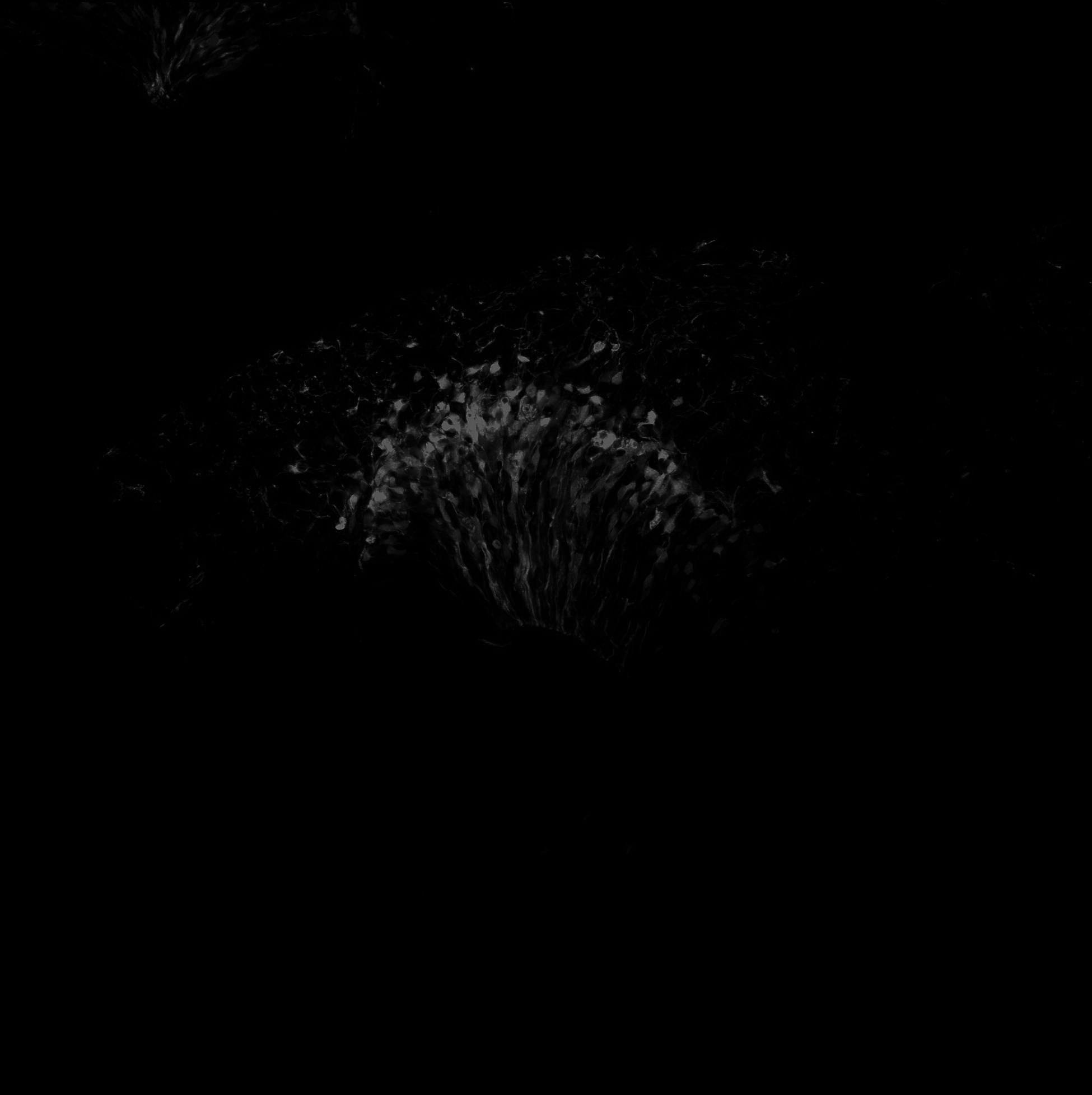

Supplement: Supplementary file 8 — Source Data for Figure 4 [file EMBR-23-e54728-s005.zip › Figure 3/3C/ARHGAP11B_4days_GFP.tif]

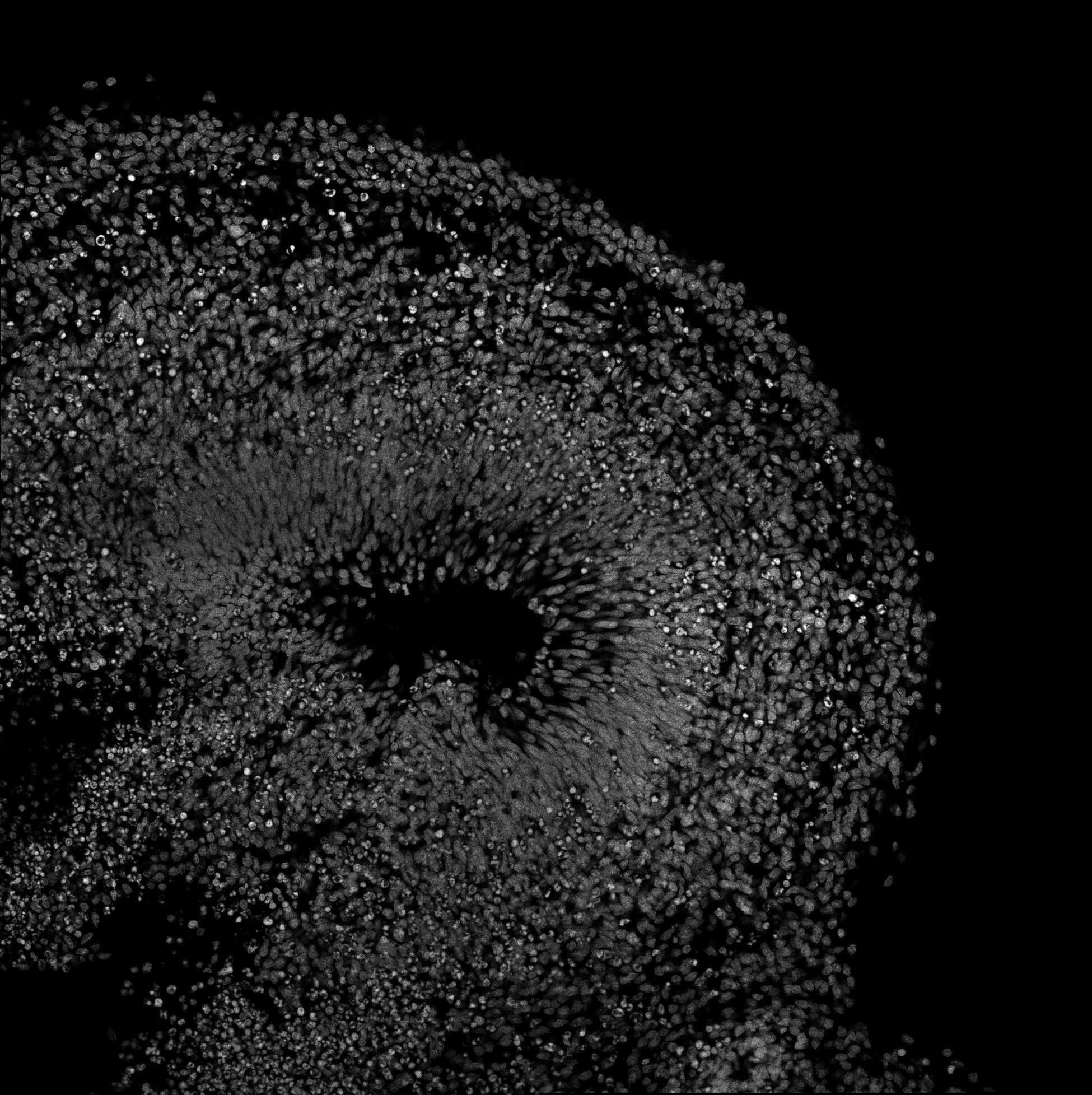

Supplement: Supplementary file 8 — Source Data for Figure 4 [file EMBR-23-e54728-s005.zip › Figure 3/3C/Control_4days_DAPI.tif]

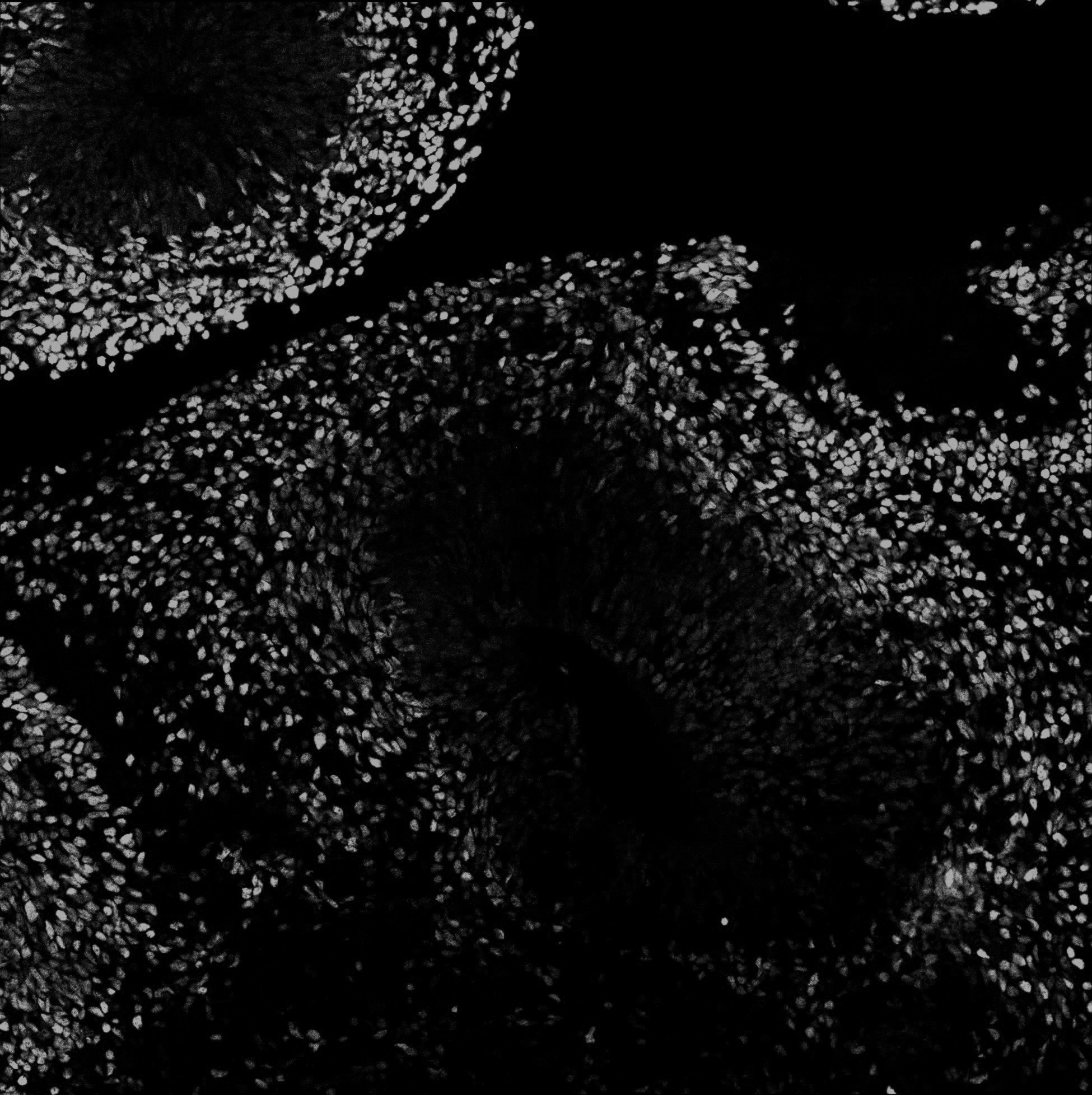

Supplement: Supplementary file 8 — Source Data for Figure 4 [file EMBR-23-e54728-s005.zip › Figure 3/3C/ARHGAP11B_4days_CTIP2.tif]

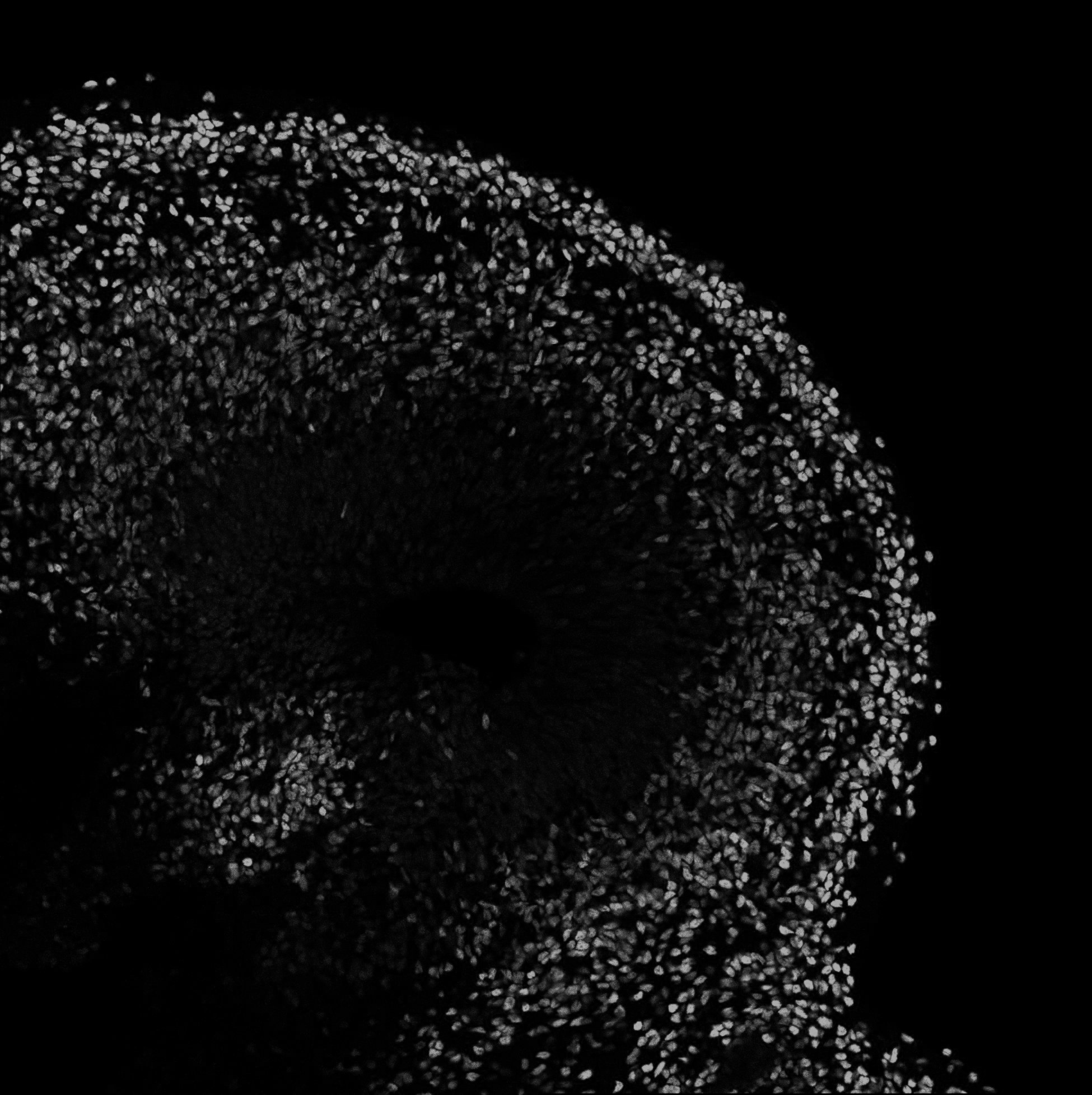

Supplement: Supplementary file 8 — Source Data for Figure 4 [file EMBR-23-e54728-s005.zip › Figure 3/3C/Control_4days_CTIP2.tif]

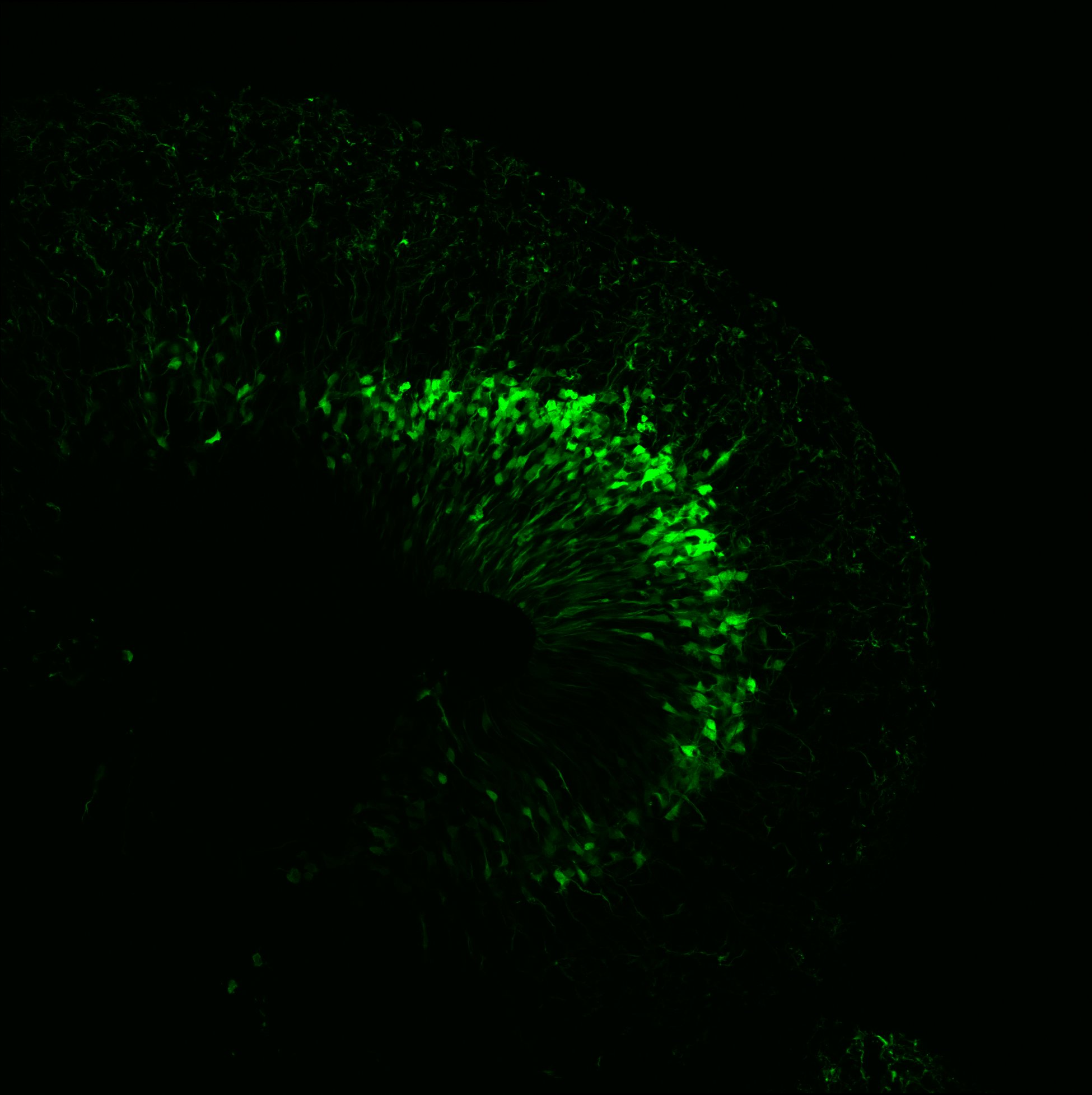

Supplement: Supplementary file 8 — Source Data for Figure 4 [file EMBR-23-e54728-s005.zip › Figure 3/3C/Control_4days_GFP.tif]

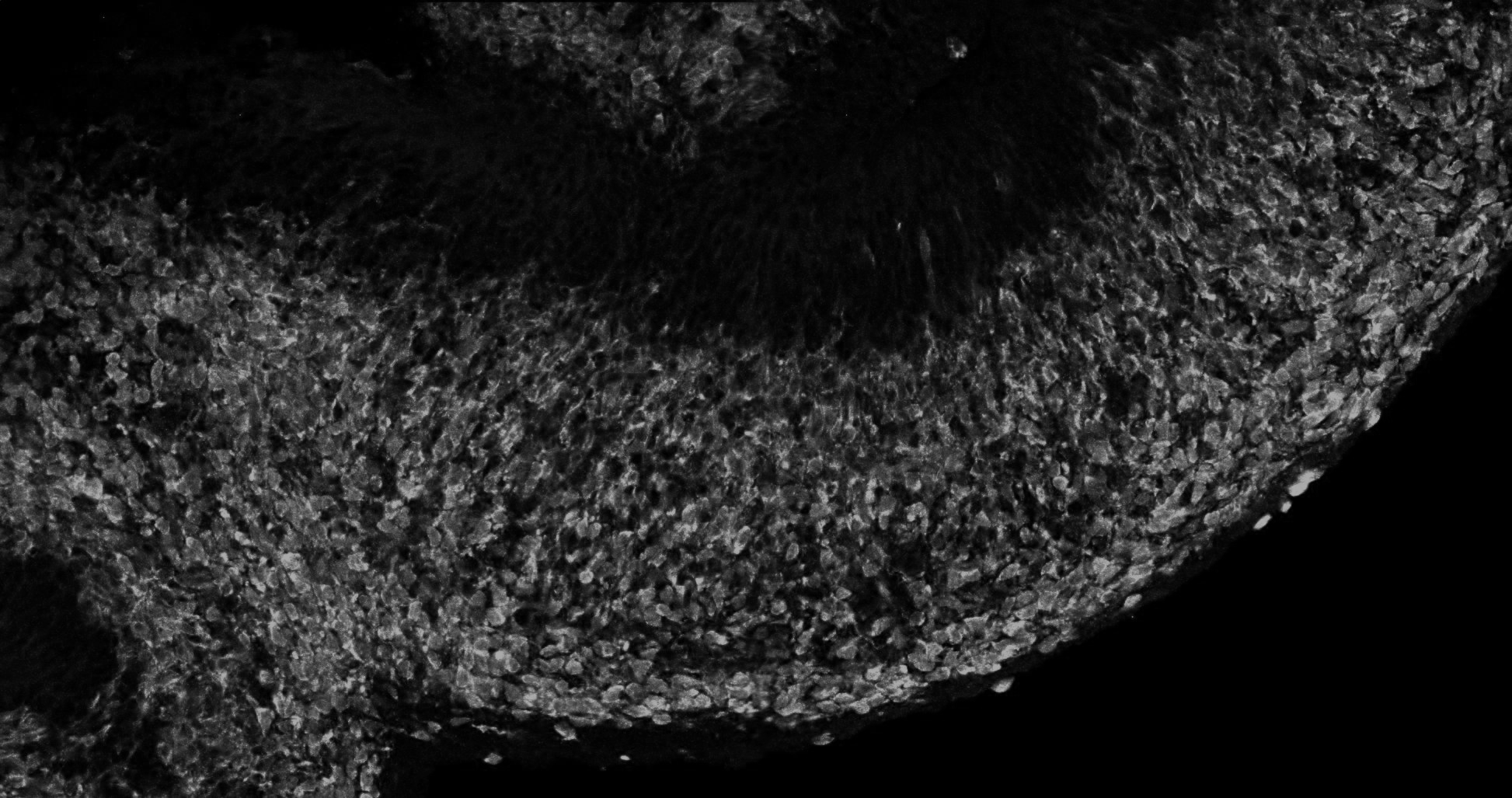

Supplement: Supplementary file 8 — Source Data for Figure 4 [file EMBR-23-e54728-s005.zip › Figure 3/3A/ARHGAP11B_10days_Hu.tif]

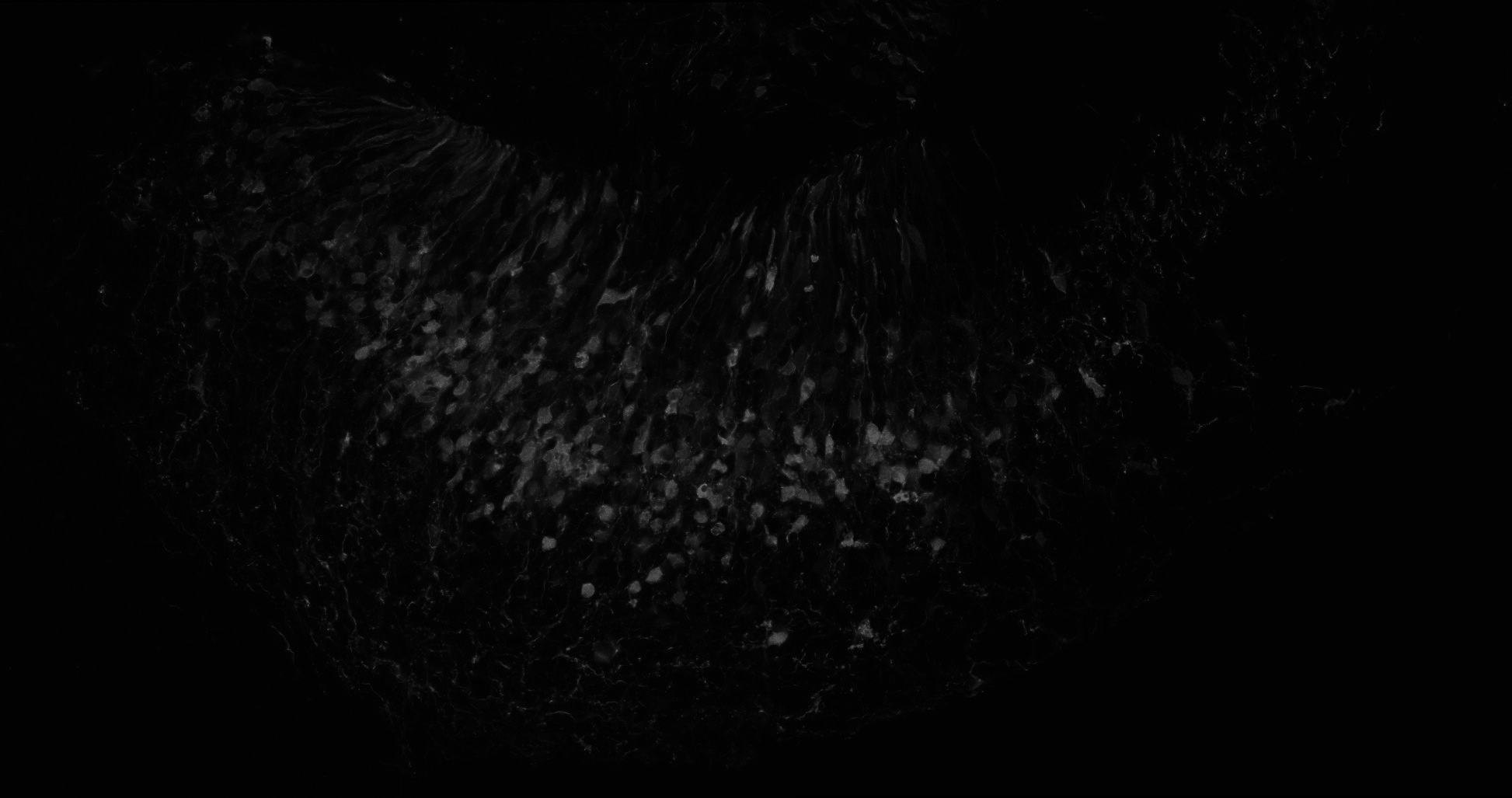

Supplement: Supplementary file 8 — Source Data for Figure 4 [file EMBR-23-e54728-s005.zip › Figure 3/3A/ARHGAP11B_10days_GFP.tif]

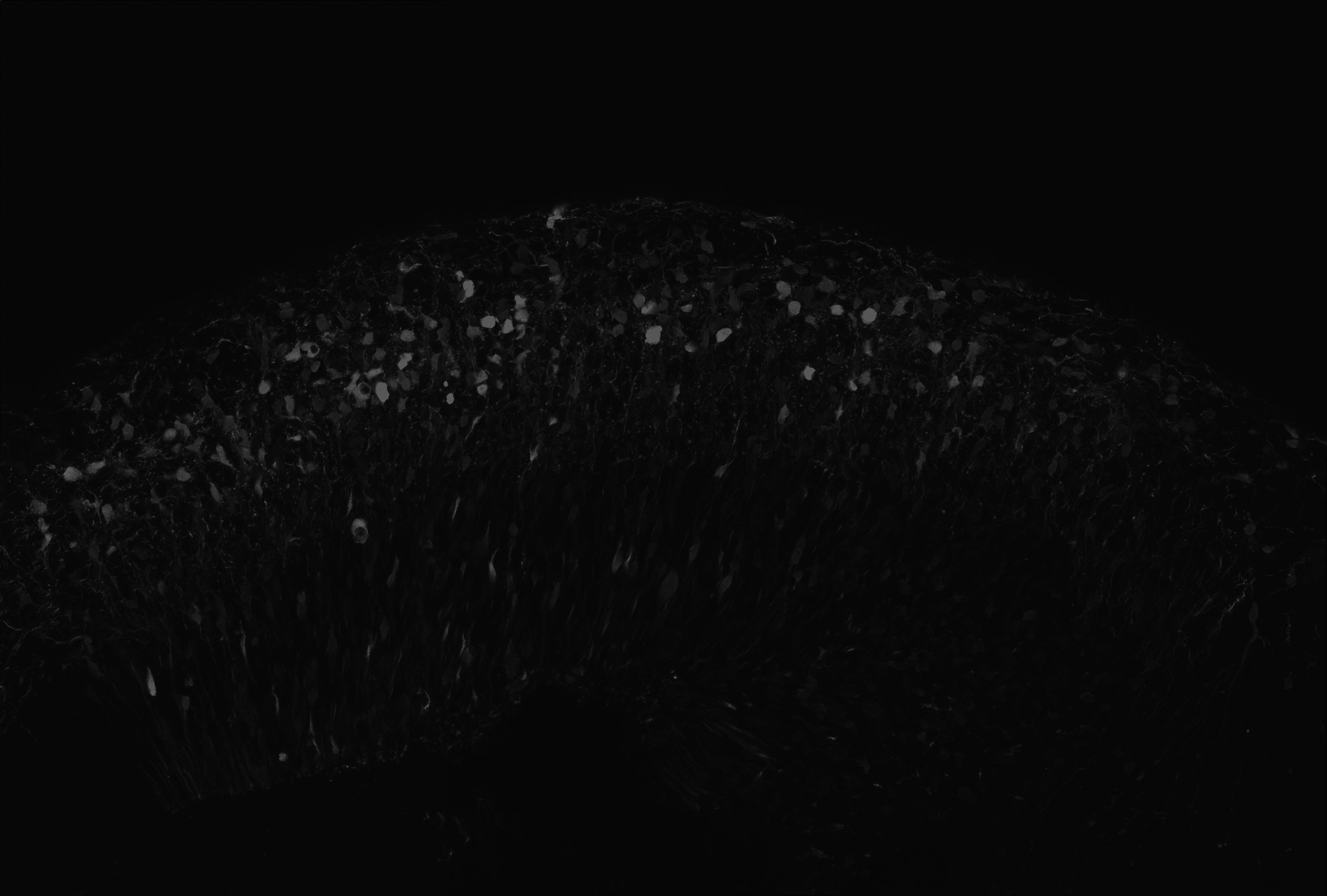

Supplement: Supplementary file 8 — Source Data for Figure 4 [file EMBR-23-e54728-s005.zip › Figure 3/3A/Control_10days_GFP.tif]

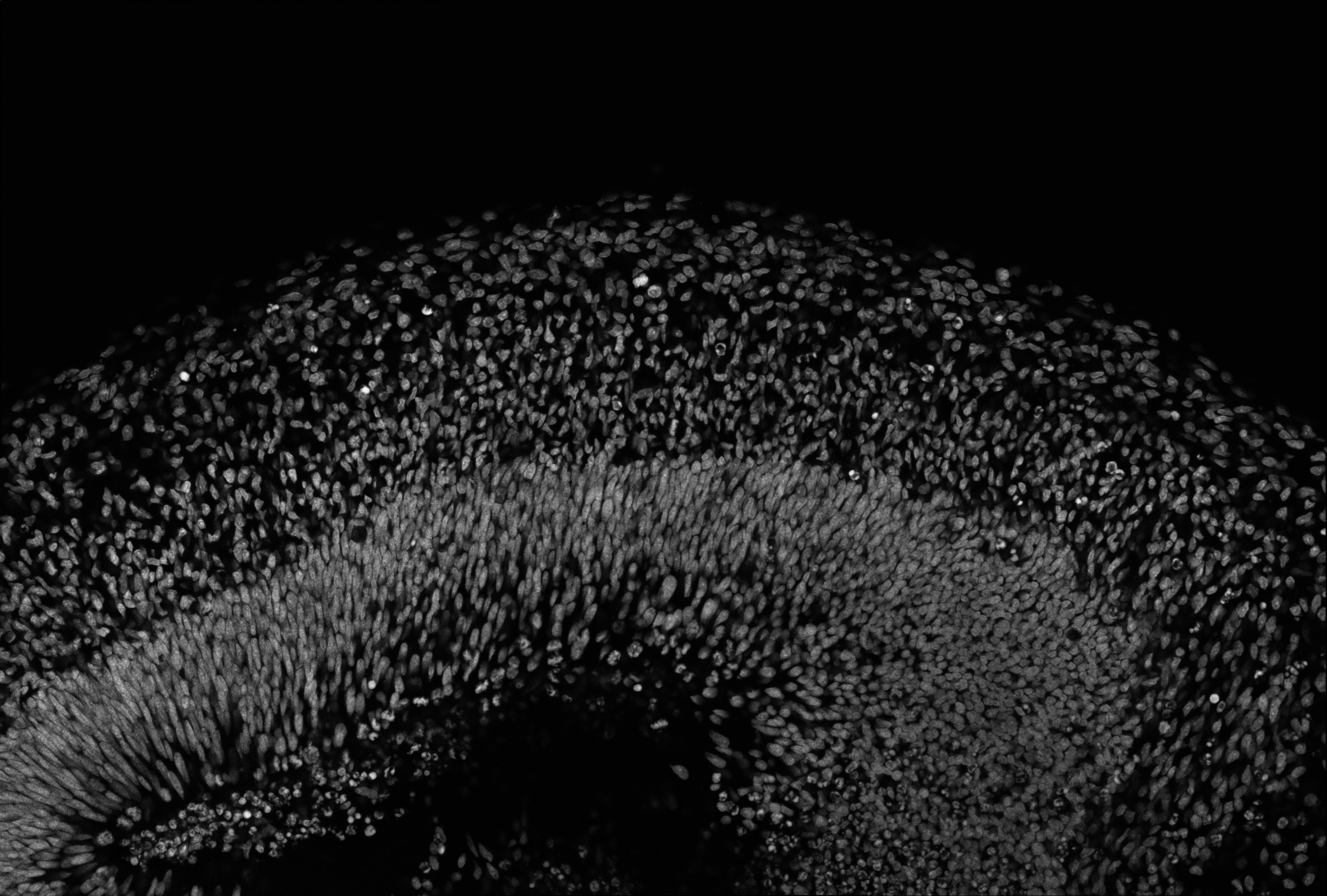

Supplement: Supplementary file 8 — Source Data for Figure 4 [file EMBR-23-e54728-s005.zip › Figure 3/3A/Control_10days_DAPI.tif]

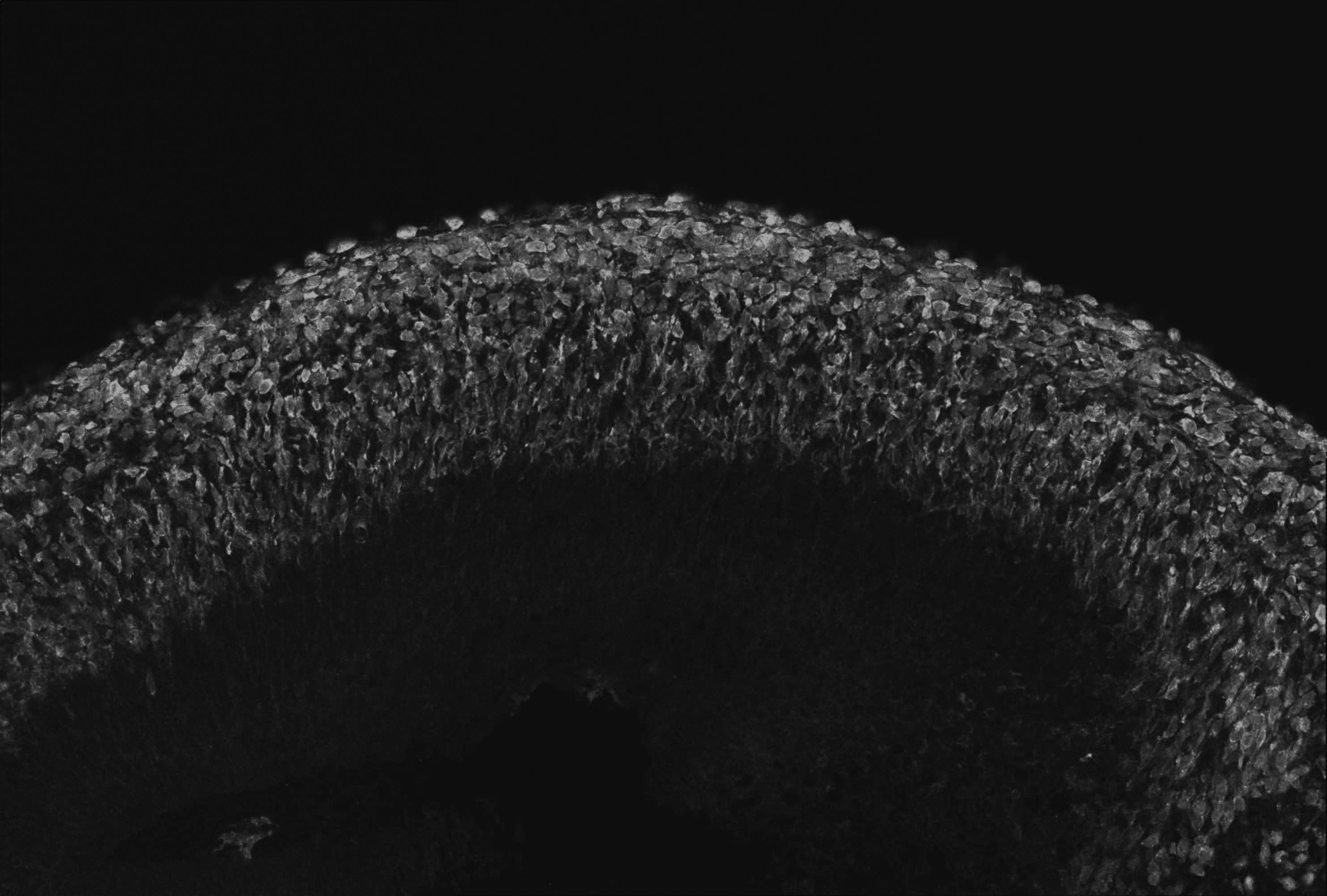

Supplement: Supplementary file 8 — Source Data for Figure 4 [file EMBR-23-e54728-s005.zip › Figure 3/3A/Control_10days_Hu.tif]

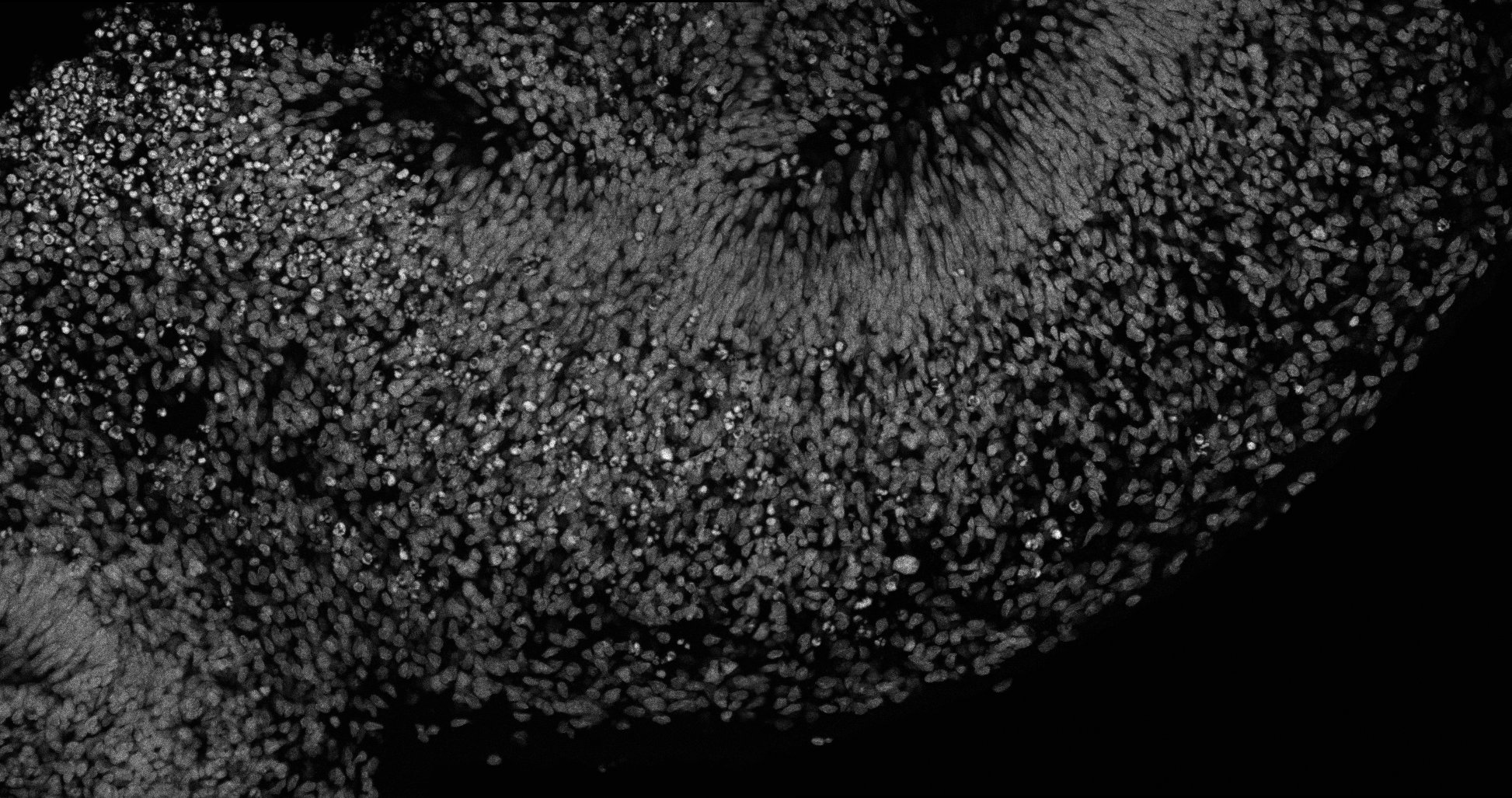

Supplement: Supplementary file 8 — Source Data for Figure 4 [file EMBR-23-e54728-s005.zip › Figure 3/3A/ARHGAP11B_10days_DAPI.tif]
