# Supplementary material for: Human‐specific ARHGAP11B ensures human‐like basal progenitor levels in hominid cerebral organoids
Source: EMBO Rep. 2022 Sep 13;23(11):e54728. doi: 10.15252/embr.202254728 (PMC9646322; doi:10.15252/embr.202254728)
Supplement: Supplementary file 9 [file EMBR-23-e54728-s006.pdf]

EMBO reports

Human-specific ARHGAP11B ensures human-like basal progenitor levels in hominid cerebral organoids

Jan Fischer, Eduardo Fernández Ortuño, Fabio Marsoner, Annasara Artioli, Jula Peters, Takashi Namba, Christina Eugster Oegema, Wieland Huttner, Julia Ladewig, and Michael Heide
DOI: 10.15252/embr.202254728

Corresponding author(s): Michael Heide (Mheide@dpz.eu) , Wieland Huttner (huttner@mpi-cbg.de), Julia Ladewig (julia.ladewig@zi-mannheim.de)

Review Timeline:

Submission Date:	25th Jan 22
Editorial Decision:	26th Jan 22
Revision Received:	24th Jun 22
Editorial Decision:	10th Aug 22
Revision Received:	18th Aug 22
Accepted:	23rd Aug 22

Editor: Esther Schnapp

Transaction Report: This manuscript was transferred to EMBO reports following peer review at The EMBO Journal.

(Note: With the exception of the correction of typographical or spelling errors that could be a source of ambiguity, letters and reports are not edited. Depending on transfer agreements, referee reports obtained elsewhere may or may not be included in this compilation. Referee reports are anonymous unless the Referee chooses to sign their reports.)

Referee #1 Review
Received: 16th Dec 21

Report for Author:
ARHGAP11B is a human-specific gene formed by the duplication of ARHGAP11A that subsequently acquired changes in splicing and gene expression. Previous studies have shown ectopic ARHGAP11B to be sufficient for increased proliferation of basal progenitors in mouse and marmoset models. Despite several important and high-profile studies, remaining unanswered questions include: the extent of ARHGAP11B's contribution to human cortical expansion, the necessity of this gene in a human context, and the molecular mechanisms by which ARHGAP11B phenotypes are realized. The current study extends previous work by studying the role of ARHGAP11A/B in the human and chimpanzee progenitor cells using brain organoids and a novel organoid electroporation technique.

The study finds that ARHGAP11B electroporation is sufficient to increase the number of basal progenitors, including basal radial glia, potentially by delaying differentiation, leading to an increased proportion of upper layer neurons, mirroring a trend associated with brain expansion in primates. Additionally, the study finds that a dominant negative ARHGAP11A impairs proliferation and basal progenitor number in human but not chimpanzee brain organoids, suggesting a greater necessity for these genes in human. Finally, homozygous CRISPR knockout of both ARHGAP11A and B paralogs in humans shows a less severe organoid basal progenitor reduction with ARHGAP11B electroporation than with ARHGAP11A, implying that ARHGAP11B is necessary for normal human organoid basal progenitor development.

The study is interesting and supports previous ARHGAP11B results in other systems, while providing new layers to our understanding of the developmental consequences of the ARHGAP11 duplication in human and chimpanzee neural progenitors, but I still have some concerns.

Major concerns:

How physiological is the upregulation of ARHGAP11B by electroporation?

The upregulation of ARHGAP11B is not benchmarked against the actual expression levels in human progenitor cells and would be reduced with each cell division through plasmid dilution. My sense is that the combination of a strong promoter and many plasmids incorporated in individual cells would lead much higher expression levels through electroporation than are found in human progenitors. Subsequently, later dilution would progressively reduce expression in progenitors in the longer time course experiments, potentially below normal levels.

On this theme, the authors should also provide more details and justification of the pCAGGS ARHGAP11B vector used - why not use the human-specific promoter as in the recent marmoset study, and for the older vector can the authors confirm that this isoform matches that expressed in the human brain, and does this also include the 3' UTR that might influence regulation?

In addition, endogenous ARHGAP11B regulation may be cell cycle-dependent (enriched in M-phase), but the ectopic expression from a constitutive promoter may lead to ectopic expression in other phases of the cell cycle that could affect interpretations by differing from physiological human expression.

The study does not provide new insights on the molecular mechanism by which ARHGAP11B influences proliferation, which may be beyond the scope, but is a major unmet need in our full understanding of the role of ARHGAP11B in human brain evolution

The study suggests a massive reduction in basal radial glia when ARHGAP11B is missing, but I have major concerns about this claim:
-First, how severe is the double knockout alone? There is no quantification of ventricle size, basal radial glia number etc in clone 16 and with electroporation of GFP only compared to wildtype lines, and this comparison would probably require multiple edited clones
-Second, the claim that ARHGAP11B is necessary for normal proliferation also requires evidence from multiple clones and/or ruling out off-target effects of the editing
-Third, for the necessity experiment it would be much better to knockout just 11A or just 11B because the over expression is not physiological. I realize it would be extremely challenging to target specific paralogs, but there may be some paralog-specific cut sites in introns. However, because of the non-physiological upregulation, the rescue experiments could simply be telling us that A has a more potent effect on proliferation than B, rather than proving B is specifically necessary in human, which is an important unmet need.

Minor concerns

-Why are the GFP and ARHGAP11B on different expression vectors - can the authors confirm the extent of co-electroporation in this model as all quantifications depend on the GFP label?

-Figure S1 would be improved with cell type markers beyond SOX2 for better supporting claims of how the cell type cohort changes following electroporation.

-From the images provided, it is difficult to see how quantifications were performed. Magnified insets in the supplement with arrows pointing to the different combinations of positive cells would help the reader evaluate and appreciate the quantifications that are central to the conclusions. There should also be more clarity on how the VZ and SVZ are defined and images should include brackets defining each region. This is a major concern as almost all analyses depend on the quantification and region definitions, which are not transparent, but hopefully should be easily addressable.

-Does ARHGAP11B overexpression in wildtype human cause NPC over-proliferation?

-Does ARHGAP11B also cause increased apical progenitor proliferation?

-How were the timepoints selected for analysis and are these comparable between species? Also, there could be more methodological details on how the authors ensure that the control and ARHGAP11B electroporation are in ventricles of similar character to start, and how does the SVZ size vary between ventricles and over time in human and chimpanzee organoids?

-Similarly, on the timepoint selection, Line 308 states implies that the first neurons are produced at day 51 in organoids, but other studies have reported neurons by 3 to 5 weeks or earlier.

-For the neuron fate changes, could ectopic ARHGAP11B expression in neurons influence their survival/identity (for example contributing to the reduction of deep layer neurons, as the earliest born neurons may inherit a high plasmid copy number for a gene not normally expressed), but then plasmid dilution could cause less influence on upper layer neuron production?

-Clarify whether ARHGAP11A220 also interferes with ARHGAP11A?

In 4B, the analysis of the fraction of BrdU+ GFP cells between human and chimp, lacks sufficient biological replication (and also does not account for potential timing differences between species) for claims about species level differences in basal progenitor number - this should be acknowledged and the claims must focus on the within species aspect for which there is substantial technical replication of the conditions. For example, line 393 "Interestingly, this reduction brought the level of these BPs down to that observed in chimpanzee" must be clarified to in this comparison of one human and one chimp individual or removed.

In 4b, was this chimpanzee phenotype from the dominant negative harder to detect simply because there were fewer dividing basal progenitors at this stage? And would this reduction in basal progenitors be true across organoids from other chimpanzee individuals as a species-specific organoid phenotype or just in the tested individual? My understanding is that the organoid field has not extensively measured variation in features of histogenesis and basal progenitor number even across human individuals.

-For Clone 16, is it worth doing Southern blots to confirm no additional integration, or sequencing to assess off-target effect, or is the point that Clone 16 provides a line for isogenic experiments testing overexpression

Other comments

-"However, the extent of ARHGAP11B's contribution to this expansion during hominid evolution is unknown" is a major question that is introduced quantitatively, but only a qualitative answer is provided. I don't think a quantitative answer is possible in this study, because with one human and one chimp line, it is not possible to measure overall species differences in basal progenitor number.

-Line 57 - could also cite earlier Sasai lab studies, such as Eiraku et al., 2008

-Lines 251 and 282 repeat almost identical language

Referee #2 Review
Received: 4th Dec 21

Report for Author:
This study by the Huttner lab investigates the effect of overexpressing the human-specific gene ARHGAP11B in iPSC-derived cerebral organoids from human and chimpanzee. They replicate the same findings already reported by the same group in mouse, ferret and marmoset, now in chimpanzee organoids, where this overexpression promotes the generation of cortical basal progenitors, a key step in the complex developmental program driving the expansion of the cerebral cortex. The study has some points of novelty, such as testing ARHGAP11B in chimp organoids or performing loss-of-function experiments in human organoids, but these are minor and overall the study brings very limited, if any, conceptual advance to this important question. There are also a number of issues that the authors should address in their manuscript:

Line 106 - "Examining human-specific genes [our results] provide corroborating evidence in support of their presumptive role in neocortical development during human evolution, but also open up new avenues to dissect their mechanism of action." This study does not really open new avenues to dissect the mechanism of action of ARHGAP11B, but only that ARHGAP11A220 is a loss-of-function.

Line 164 - Introduction is long and circular around known and unknown the functions of ARHGAP11B, and open questions, but in the end it is very unclear what the aim of the study really is. The authors should improve or establish a hierarchy in their ideas exposed in relation to the importance of the open questions, much necessary to focalize the attention of readers.

Line 263 - "bRG are thought to be key for mammalian neocortex evolution and to drive expansion and folding of the human neocortex" - Here, the authors should cite more recent and comprehensive reviews on expansion and folding of the neocortex, such as for example the nice review by Borrell and colleagues in Nat Rev Neurosci, 2019.

In spite of the use of Hopx as a marker of bRG in several studies, mounting evidence in the field shows that this is really not a bRG marker. It is in fact striking that the authors claim the utility of this as bRG marker, since the stains in Figure 2 show many aRGs in VZ positive for Hopx. Significantly, in addition to Hopx not being a marker of bRG, the authors are not showing any of the typical signs of bRG for which these are distinct from other BPs: basal radial process and lack of apical process.

Line 304 - "We conclude that the increased abundance of cycling BPs observed after a 10-day period of expression of ARHGAP11B in chimpanzee cerebral organoids reflects an increased generation of BPs from BPs, at the expense of the generation of cortical neurons from BPs" - The absence of neuron marker expression does not demonstrate progenitor identity, so this conclusion is not supported by the observations. Instead, the authors should demonstrate increased cell cycle re-entry,

or increased proportion of Ki67+ cells among GFP+ cells.

Line 321 - "the first neurons generated by BPs in chimpanzee cerebral organoids, the generation of which is reduced upon ARHGAP11B expression due to the increased generation of BPs, are of the deep-layer type" - Was there an increase in Satb2+ neurons at this early time-point? Maybe ARHGAP11B promotes a shift from deep to superficial layer neuron type, without an actual delay in neurogenesis. Otherwise, this would mean an abnormal deficit in deep layer neurons, which is not observed in human cortex. Also, the hypothesis that delayed neurogenesis would altogether skip deep-layer neurogenesis is flawed, as delayed developmental programs usually resume following all steps of neurogenesis, even if these are overall shortened.

Line 389 - "TBR2, a marker of BPs (Englund et al., 2005, Sessa et al., 2008) that in fetal human neocortex is typically expressed in the basal intermediate progenitor (bIP) subpopulation of BPs (Hevner, 2019, Mihalas et al., 2016)." - None of these two articles show that human neocortex bIPs express Tbr2, nor that it is a marker for them. Contrarily, multiple other studies show that bRGs in non-mouse species co-express Pax6 and Tbr2. Why the abundance of bRGs was not studied in these experiments?

Line 439 - "we investigated whether the ARHGAP11B knockout resulted in a decreased BP abundance in the human forebrain organoids. Immunohistochemistry for the bRG marker PTPRZ1 (Pollen et al., 2015) (Figure 5A)" - Why the authors used here a different bRG marker? Why did they wait for 10 days after electroporation instead of 4 days, as above? Were control organoids isogenic with the mutants?

Line 447 - "Together with the results of the dominant-negative ARHGAP11A220 in human cerebral organoids (Figure 4), these data demonstrate that ARHGAP11B is required to maintain the elevated levels of cycling BPs that are characteristic of human cerebral cortex tissue." - Prior to rescue, were double-KO organoids different in any way from wild-type isogenic organoids? Did the absence of ARHGAP11B affect only after GFP electroporation?

Results in Figure 5B for ARHGAP11A KO are apparently bimodally distributed, which is not the case for ARHGAP11B. What is the cause of that? Different organoid batches? Does this mean partial penetrance of the phenotype, where half of AKOs were also affected? How is this interpreted?

Discussion, line 485 - "ARHGAP11B alone is capable of increasing the abundance of cycling BPs to a human-like level and hence of providing a crucial basis for the evolutionary expansion of the human neocortex." - Did ARHGAP11B function ultimately influence the amount of cortical neurogenesis? Were human organoids smaller when blocking ARHGAP11B function, or chimp organoids larger when OE this gene? That is, were increased BPs functional? Neurogenic?

Minor point: The limits of SVZ need to be indicated in the figures across the manuscript, as well as GFP+ cells positive for the studied markers.

1st Editorial Decision 26th Jan 2022

Dear Dr. Heide,

Thank you for the transfer of your manuscript and referee reports to EMBO reports.

I have now carefully read your proposed point-by-point response and I would like to invite you to revise your manuscript along the lines you suggest.

Please note that the referee concerns must be fully addressed and their suggestions taken on board. Please address all referee concerns in a complete point-by-point response. Acceptance of the manuscript will depend on a positive outcome of a second round of review. It is EMBO reports policy to allow a single round of major revision only and acceptance or rejection of the manuscript will therefore depend on the completeness of your responses included in the next, final version of the manuscript.

We realize that it is difficult to revise to a specific deadline. In the interest of protecting the conceptual advance provided by the work, we recommend a revision within 3 months (28th Apr 2022). Please discuss the revision progress ahead of this time with the editor if you require more time to complete the revisions.

You can either publish the study as a short report or as a full article. For short reports, the revised manuscript should not exceed 27,000 characters (including spaces but excluding materials & methods and references) and 5 main plus 5 expanded view figures. The results and discussion sections must further be combined, which will help to shorten the manuscript text by eliminating some redundancy that is inevitable when discussing the same experiments twice. For a normal article there are no length limitations, but it should have more than 5 main figures and the results and discussion sections must be separate.

IMPORTANT NOTE: we perform an initial quality control of all revised manuscripts before re-review. Your manuscript will FAIL this control and the handling will be DELAYED if the following APPLIES:
1) A data availability section providing access to data deposited in public databases is missing. If you have not deposited any data, please add a sentence to the data availability section that explains that.
2) Your manuscript contains statistics and error bars based on n=2. Please use scatter blots in these cases. No statistics should be calculated if n=2.

When submitting your revised manuscript, please carefully review the instructions that follow below. Failure to include requested items will delay the evaluation of your revision.

1) a .docx formatted version of the manuscript text (including legends for main figures, EV figures and tables). Please make sure that the changes are highlighted to be clearly visible.

2) individual production quality figure files as .eps, .tif, .jpg (one file per figure). See https://wol-prod-cdn.literatumonline.com/pb-assets/embo-site/EMBOPress_Figure_Guidelines_061115-1561436025777.pdf for more info on how to prepare your figures.

3) We replaced Supplementary Information with Expanded View (EV) Figures and Tables that are collapsible/expandable online. A maximum of 5 EV Figures can be typeset. EV Figures should be cited as 'Figure EV1, Figure EV2" etc... in the text and their respective legends should be included in the main text after the legends of regular figures.

- For the figures that you do NOT wish to display as Expanded View figures, they should be bundled together with their legends in a single PDF file called *Appendix*, which should start with a short Table of Content. Appendix figures should be referred to in the main text as: "Appendix Figure S1, Appendix Figure S2" etc. See detailed instructions regarding expanded view here: <https://www.embopress.org/page/journal/14693178/authorguide#expandedview>

- Additional Tables/Datasets should be labeled and referred to as Table EV1, Dataset EV1, etc. Legends have to be provided in a separate tab in case of .xls files. Alternatively, the legend can be supplied as a separate text file (README) and zipped together with the Table/Dataset file.

4) a .docx formatted letter INCLUDING the reviewers' reports and your detailed point-by-point responses to their comments. As part of the EMBO Press transparent editorial process, the point-by-point response is part of the Review Process File (RPF), which will be published alongside your paper.

5) a complete author checklist, which you can download from our author guidelines <https://www.embopress.org/page/journal/14693178/authorguide>. Please insert information in the checklist that is also reflected in the manuscript. The completed author checklist will also be part of the RPF.

6) Please note that all corresponding authors are required to supply an ORCID ID for their name upon submission of a revised manuscript (<https://orcid.org/>). Please find instructions on how to link your ORCID ID to your account in our manuscript tracking system in our Author guidelines

<https://www.embopress.org/page/journal/14693178/authorguide#authorshipguidelines>

7) Before submitting your revision, primary datasets produced in this study need to be deposited in an appropriate public database (see https://www.embopress.org/page/journal/14693178/authorguide#datadeposition). Please remember to provide a reviewer password if the datasets are not yet public. The accession numbers and database should be listed in a formal "Data Availability" section placed after Materials & Method (see also https://www.embopress.org/page/journal/14693178/authorguide#datadeposition). Please note that the Data Availability Section is restricted to new primary data that are part of this study. * Note - All links should resolve to a page where the data can be accessed. *
If your study has not produced novel datasets, please mention this fact in the Data Availability Section.

8) We would also encourage you to include the source data for figure panels that show essential data. Numerical data should be provided as individual .xls or .csv files (including a tab describing the data). For blots or microscopy, uncropped images should be submitted (using a zip archive if multiple images need to be supplied for one panel). Additional information on source data and instruction on how to label the files are available at <https://www.embopress.org/page/journal/14693178/authorguide#sourcedata>.

9) Our journal also encourages inclusion of *data citations in the reference list* to directly cite datasets that were re-used and obtained from public databases. Data citations in the article text are distinct from normal bibliographical citations and should directly link to the database records from which the data can be accessed. In the main text, data citations are formatted as follows: "Data ref: Smith et al, 2001" or "Data ref: NCBI Sequence Read Archive PRJNA342805, 2017". In the Reference list, data citations must be labeled with "[DATASET]". A data reference must provide the database name, accession number/identifiers and a resolvable link to the landing page from which the data can be accessed at the end of the reference. Further instructions are available at https://www.embopress.org/page/journal/14693178/authorguide#referencesformat

10) Regarding data quantification (see Figure Legends: https://www.embopress.org/page/journal/14693178/authorguide#figureformat)

The following points must be specified in each figure legend:

- the name of the statistical test used to generate error bars and P values,

- the number (n) of independent experiments (please specify technical or biological replicates) underlying each data point,

- the nature of the bars and error bars (s.d., s.e.m.),

- If the data are obtained from n {less than or equal to} 2, use scatter blots showing the individual data points.

Discussion of statistical methodology can be reported in the materials and methods section, but figure legends should contain a basic description of n, P and the test applied.

- Please also include scale bars in all microscopy images.

11) The journal requires a statement specifying whether or not authors have competing interests (defined as all potential or actual interests that could be perceived to influence the presentation or interpretation of an article). In case of competing interests, this must be specified in your disclosure statement. Further information: https://www.embopress.org/competing-interests

We would also welcome the submission of cover suggestions, or motifs to be used by our Graphics Illustrator in designing a cover.

As part of the EMBO publication's Transparent Editorial Process, EMBO reports publishes online a Review Process File (RPF) to accompany accepted manuscripts. This File will be published in conjunction with your paper and will include the referee reports, your point-by-point response and all pertinent correspondence relating to the manuscript.

You are able to opt out of this by letting the editorial office know (emboreports@embo.org). If you do opt out, the Review Process File link will point to the following statement: "No Review Process File is available with this article, as the authors have chosen not to make the review process public in this case."

I look forward to seeing a revised form of your manuscript when it is ready. Please use this link to submit your revision: https://embor.msubmit.net/cgi-bin/main.plex

Best regards,
Esther

Esther Schnapp, PhD
Senior Editor
EMBO reports

Response to Reviewers – Overview of Revision

Figure	Revision	Contents	Reviewer(s)
**Fig 1**	**Revised** Panels A and C	*Indication of VZ and SVZ/NL and of examples of GFP and TBR2 double-positive, and of GFP, TBR2 and Ki67 triple-positive cells.*	#1,2
**Fig 2**	**Revised** Panels A and C	*Indication of VZ and SVZ/NL and of examples of GFP and HOPX double-positive cells.*	#1,2
**Fig 3**	**Revised** Panels A, C and E	*Indication of VZ and SVZ/NL and of examples of GFP and Hu double-positive, GFP and CTIP2 double-positive, and of GFP and SATB2 triple-positive cells.*	#1,2
**Fig 4**	**Revised** Panels A and C	*Indication of VZ and SVZ/NL and of examples of GFP and BrdU double-, and of GFP and TBR2 double-positive cells.*	#1,2
**Fig 5**	**Revised** Panel A	*Indication of VZ and SVZ/NL and of examples of GFP and PTPRZ1 double-positive cells.*	#1,2
	**Revised** Panel B	*Addition of data from a second independent ARHGAP11A and ARHGAP11B double-knockout clone.*	#1,2
**Fig EV1**	**Revised** Panel D	Indication of VZ and SVZ/NL	#1,2
**NEW Fig EV2**		*Co-electroporation efficiency of GFP and ARHGAP11B in chimpanzee cerebral organoids*	#1
**Fig EV3**	**Revised** Panel A	*Indication of VZ and SVZ/NL and of examples of GFP and PCNA double-positive cells.*	#1,2
**Fig EV4**	**Revised** Panel A	*Indication of VZ and SVZ/NL and of examples of GFP and NeuN double-positive cells.*	#1,2
**Fig EV5**	**Revised** Panel A	*Indication of the second iPSC line (B7_028#4)*	#1,2

	**Revised** Panel B	*Genomic PCR validation for integration of the HDR template of clone j*	#1,2
	**Revised** Panel C, D	*Addition of data from clone j to the analysis of the expression levels of ARHGAP11B (C) and ARHGAP11B (D)*	#1,2
	**Revised** Panel E	*Addition of a sequencing analysis for clone j*	#1,2
**NEW Appendix Fig S1**		*Localization of TBR2-positive and GFP-positive cells in electroporated chimpanzee cerebral organoids*	#1,2
**NEW Appendix Fig S2**		*Localization of CTIP2-positive and GFP-positive cells in electroporated chimpanzee cerebral organoids*	#1,2
**NEW Appendix Fig S3**		*Localization of SATB2-positive and GFP-positive cells in electroporated chimpanzee cerebral organoids*	#1,2
**NEW Appendix Fig S4**		*Quantification of the proportion of GFP-positive cells in the SVZ that are PTPRZ1-positive in 60 days-old WT human forebrain organoids 10 days after electroporation with either control plasmid only, ARHGAP11A expression plasmid, ARHGAP11B expression plasmid or ARGHAP11A plus ARHGAP11B expression plasmids.*	#1
**NEW Table EV1**		*Potential off-target integration sites of clone 16 and clone j*	#1
**NEW Table EV2**		*Read clusters of clone 16 vs. control*	#1
**NEW Table EV3**		*Read clusters of clone j vs. control*	#1

Response to the Reviewers

General Response: We would like to thank the reviewers for the time and effort they spent on the manuscript. Their constructive criticism has encouraged us to examine selected aspects more closely. We feel that the reviewers' valuable comments and suggestions allowed us to significantly improve the manuscript, and we hope that the reviewers will find this revised version acceptable for publication.

Referee #1:

Referee's comment:
ARHGAP11B is a human-specific gene formed by the duplication of ARHGAP11A that subsequently acquired changes in splicing and gene expression. Previous studies have shown ectopic ARHGAP11B to be sufficient for increased proliferation of basal progenitors in mouse and marmoset models. Despite several important and high-profile studies, remaining unanswered questions include: the extent of ARHGAP11B's contribution to human cortical expansion, the necessity of this gene in a human context, and the molecular mechanisms by which ARHGAP11B phenotypes are realized. The current study extends previous work by studying the role of ARHGAP11A/B in the human and chimpanzee progenitor cells using brain organoids and a novel organoid electroporation technique.

The study finds that ARHGAP11B electroporation is sufficient to increase the number of basal progenitors, including basal radial glia, potentially by delaying differentiation, leading to an increased proportion of upper layer neurons, mirroring a trend associated with brain expansion in primates. Additionally, the study finds that a dominant negative ARHGAP11A impairs proliferation and basal progenitor number in human but not chimpanzee brain organoids, suggesting a greater necessity for these genes in human. Finally, homozygous CRISPR knockout of both ARHGAP11A and B paralogs in humans shows a less severe organoid basal progenitor reduction with ARHGAP11B electroporation than with ARHGAP11A, implying that ARHGAP11B is necessary for normal human organoid basal progenitor development.

The study is interesting and supports previous ARHGAP11B results in other systems, while providing new layers to our understanding of the developmental consequences of the ARHGAP11 duplication in human and chimpanzee neural progenitors, but I still have some concerns.

Authors' response:
We very much appreciate the positive and insightful evaluation by the Reviewer and we thank the Reviewer for the many detailed comments and suggestions, which helped to substantially improve our manuscript. All new analyses and experimental data are described in the point-by-point response below.

Referee's comment:
Major concerns:

How physiological is the upregulation of ARHGAP11B by electroporation?

Authors' response:
The level of *ARHGAP11B* expression under the control of the constitutive promoter used is likely higher than under physiological conditions. However, previous studies with this promoter studying the effects of *ARHGAP11B* upon *in utero* electroporation in mouse and ferret embryos (Florio et al. 2015 *Science* 347, 1465; Florio et al. 2016 *Sci. Adv.* 2, e160194; Kalebic et al. 2018 *eLife* 7, e41241) have established that this is a valid approach to study the functions of ARHGAP11B. We have clarified this in the revised manuscript (see lines 682-699).

Referee's comment:
The upregulation of ARHGAP11B is not benchmarked against the actual expression levels in human progenitor cells and would be reduced with each cell division through plasmid dilution. My sense is that the combination of a strong promoter and many plasmids incorporated in individual cells would lead much higher expression levels through electroporation than are found in human progenitors. Subsequently, later dilution would progressively reduce expression in progenitors in the longer time course experiments, potentially below normal levels.

Authors' response:
While the plasmid dilution aspect mentioned by the Reviewer is, of course, correct, two further aspects should be considered in this context. First, the ARHGAP11B protein generated from the expression plasmid will also be inherited by the progeny of the transfected cells. Together with the inheritance of the expression plasmid, this should ensure sufficient ARHGAP11B protein levels in the progeny of the transfected cells to drive the machinery downstream of ARHGAP11B. Consistent with this assumption, we observe the effect of ARHGAP11B expression on the level of basal progenitors, notably basal radial glia, that is, their doubling, equally at 2, 4 and 10 days post-electroporation, although several rounds of progenitor cell division will have occurred between day 2 and day 10. Second, the cell population with the highest level of *ARHGAP11B* expression plasmid, i.e. the apical progenitors that are the primary target of electroporation, have been found to not show an increase in their pool size upon *ARHGAP11B* expression (Florio et al. 2015 *Science* 347, 1465), indicating that the receptiveness of the progenitor cell type (apical vs. basal progenitor) rather than changes in the ARHGAP11B expression level are the major determinant for ARHGAP11B's effects.

We thank the Reviewer for this insightful comment. We have addressed this issue in the revised manuscript along the lines of consideration described above (see lines 493-509).

Referee's comment:
On this theme, the authors should also provide more details and justification of the pCAGGS ARHGAP11B vector used - why not use the human-specific promoter as in the recent marmoset study, and for the older vector can the authors confirm that this isoform matches that expressed in the human brain, and does this also include the 3' UTR that might influence regulation?

Authors' response:
In line with the suggestion of the Reviewer, we indeed considered using the ≈3 kb human *ARHGAP11B* promoter rather than the CAGGS promoter. However, we found that, in contrast to the lentivirus injection into oocytes in the marmoset study, the efficiency of

ARHGAP11B expression upon electroporation of a human *ARHGAP11B* promoter–containing construct was rather low. This may reflect either the greater size of this construct as compared to the pCAGGS-*ARHGAP11B* vector, which may reduce its entry into the transfected cells, or the lack of efficient entry into the nucleus of the electroporated cells to reach the relevant transcription machinery.

In answer to the Reviewer's question, yes, the *ARHGAP11B* that is expressed from the pCAGGS vector matches the isoform expressed in the human brain. The pCAGGS-*ARHGAP11B* vector does not include the 3' UTR of *ARHGAP11B*, as this, again, would have increased the size of the expression plasmid and hence reduced the efficiency of electroporation. We have added these considerations to the revised manuscript (see lines 682-699).

<u>*Referee's comment:*</u>
In addition, endogenous ARHGAP11B regulation may be cell cycle-dependent (enriched in M-phase), but the ectopic expression from a constitutive promoter may lead to ectopic expression in other phases of the cell cycle that could affect interpretations by differing from physiological human expression.

<u>Authors' response:</u>
The previous reports studying the effects of *ARHGAP11B* expression using the constitutive CAGGS promoter in mouse and ferret embryos (Florio et al. 2015 *Science* 347, 1465; Florio et al. 2016 *Sci. Adv.* 2, e160194; Kalebic et al. 2018 *eLife* 7, e41241) vs. using the physiological human promoter in marmoset fetuses (Heide et al. 2020 *Science* 369, 546) have shown that the effects on basal progenitor levels and upper-layer neuron levels are essentially the same for these two types of *ARHGAP11B* expression. In our opinion, this implies that the use of the constitutive CAGGS promoter, despite its differences when compared to the physiological human promoter, is a valid approach to study the function of ARHGAP11B. We have explained this rationale in the revised manuscript (see lines 682-699).

To the best of our knowledge, an enrichment of *ARHGAP11B* expression in M-phase has not (yet) been reported. However, the Reviewer's comment raises an interesting scenario.

<u>*Referee's comment:*</u>
The study does not provide new insights on the molecular mechanism by which ARHGAP11B influences proliferation, which may be beyond the scope, but is a major unmet need in our full understanding of the role of ARHGAP11B in human brain evolution

<u>Authors' response:</u>
The molecular mechanism by which ARHGAP11B influences proliferation, that is, an increase in glutaminolysis due to its action in mitochondria, has previously been reported (Namba et al. 2020 *Neuron* 105, 867).

<u>*Referee's comment:*</u>
The study suggests a massive reduction in basal radial glia when ARHGAP11B is missing, but I have major concerns about this claim:
-First, how severe is the double knockout alone? There is no quantification of ventricle size,

basal radial glia number etc in clone 16 and with electroporation of GFP only compared to wildtype lines, and this comparison would probably require multiple edited clones

Authors' response:
The reason why we did not show data for the double knockout alone is that an interpretation of these data would be difficult, as it would be unclear to which extent a reduction in the level of basal radial glia would be due to loss of *ARHGAP11A* or *ARHGAP11B* or both. In our opinion, using organoids grown from the double knockout clone 16 and then transfecting their ventricles with both *ARHGAP11A* and *ARHGAP11B* (i.e., double rescue) as control is a "cleaner" approach, as it allows to study the effects of single knockout of either *ARHGAP11A* or *ARHGAP11B* by performing a single rescue via transfection of either *ARHGAP11B* or *ARHGAP11A*, respectively. In this context, we would like to refer the Reviewer also to our response below regarding the question whether *ARHGAP11B* overexpression in wildtype human organoids causes basal progenitor over-proliferation.

As to performing similar experiments with another double knockout clone derived from another genetic background, we indeed have done so for the revised version of our manuscript as we do have such a clone (please see our next response below).

Referee's comment:
-Second, the claim that ARHGAP11B is necessary for normal proliferation also requires evidence from multiple clones and/or ruling out off-target effects of the editing

Authors' response:
In line with the Reviewer's comment, for the revised version of our manuscript, we grew organoids from an independent double knockout clone (clone j) derived from another genetic background (iPSC line B7_028#4) and then transfected their ventricles with both *ARHGAP11A* and *ARHGAP11B* (control) or only with either *ARHGAP11A* or *ARHGAP11B* (single knockouts), similar to Figure 5 of our original manuscript (see new Figure 5 and EV5). We hope that the reviewer can appreciate that the newly analyzed clone behaves very similar in our rescue approach to clone 16 supporting the role of ARHGAP11B in bRG cell proliferation.

In addition, we have examined both double knockout clones for potential off-target integrations from the editing by Whole Genome Sequencing. Here we found that none of the potential off-target integrations identical between the two ARHGAP11A and ARHGAP11B double-knockout clones indicating that any effect observed for both *ARHGAP11A* and *ARHGAP11B* double knockout clones would not be due to an off-target integration but rather specific to the double knockout (see lines 449-454 and Table EV1).

Referee's comment:
-Third, for the necessity experiment it would be much better to knockout just 11A or just 11B because the over expression is not physiological. I realize it would be extremely challenging to target specific paralogs, but there may be some paralog-specific cut sites in introns. However, because of the non-physiological upregulation, the rescue experiments could simply be telling us that A has a more potent effect on proliferation than B, rather than proving B is specifically necessary in human, which is an important unmet need.

Authors' response:

Before generating double knockout clones, we actually did screen the entire *ARHGAP11A* and *ARHGAP11B* genome sequences (exons plus introns) for suitable CRISPR/Cas9 cut sites to perform a specific *ARHGAP11A* or *ARHGAP11B* single knockout, and found none. This is why we then adopted the double knockout / single rescue approach. We have clarified this point in the revised manuscript (see lines 428-430).

As to the Reviewer's comment *"the rescue experiments could simply be telling us that A has a more potent effect on proliferation than B, rather than proving B is specifically necessary in human"*, we do not see how this interpretation would be compatible with the data shown in Figure 5 of our original manuscript, as the lowest level of basal radial glia was observed upon transfection of *ARHGAP11A* into the double knockout ventricles. If the Reviewer, when using the phrase *"more potent effect on proliferation"*, is thinking of an inhibitory action of *ARHGAP11A*, then this could indeed be an alternative scenario. However, one of our groups previously reported that overexpression of *ARHGAP11A* in embryonic mouse neocortex does not lead to a significant decrease in basal progenitor levels (Fig. S12 in Florio et al. 2015 *Science* 347, 1465), so this scenario appears unlikely.

We would like to point out that we obtained essentially the same result, that is, a massive decrease in basal progenitor levels, upon both, (i) the specific *ARHGAP11B* knockout via the selective *ARHGAP11A* rescue in the double knockout ventricles, and (ii) the inhibition of ARHGAP11B function by overexpression of the dominant-negative ARHGAP11A220 (which is a specific approach as there was no effect in the *ARHGAP11B*-lacking chimpanzee organoids). When we consider these two sets of findings together, we therefore think the most plausible conclusion that can be drawn from our data is that ARHGAP11B has a major role in maintaining the elevated basal progenitor levels in human cerebral organoids.

Referee's comment:
Minor concerns

-Why are the GFP and ARHGAP11B on different expression vectors - can the authors confirm the extent of co-electroporation in this model as all quantifications depend on the GFP label?

Authors' response:
Yes, we know that upon co-transfection of the *ARHGAP11B* expression plasmid and the *GFP* expression plasmid, there is a virtually complete overlap in the cellular distribution of ARHGAP11B and GFP. We have added these data to the revised manuscript (see lines 225-230 and Fig EV2).

Referee's comment:
-Figure S1 would be improved with cell type markers beyond SOX2 for better supporting claims of how the cell type cohort changes following electroporation.

Authors' response:
We thank the Reviewer for this suggestion. As suggested, in the revised manuscript, we have added immunostainings for TBR2, CTIP2 (a deep-layer neuron marker) and SATB2 (an upper-layer neuron marker) (see Appendix Figure S1-S3).

Referee's comment:
in the supplement with arrows pointing to the different combinations of positive cells would help the reader evaluate and appreciate the quantifications that are central to the conclusions. There should also be more clarity on how the VZ and SVZ are defined and images should include brackets defining each region. This is a major concern as almost all analyses depend on the quantification and region definitions, which are not transparent, but hopefully should be easily addressable.

Authors' response:
We thank the Reviewer also for this suggestion. In the revised manuscript, we have added arrowheads pointing to the different combinations of positive cells to the relevant figure panels. Also, we have indicated the borders between VZ and SVZ/CP and explain in the Materials and Methods section (see lines 773-777) how these distinct germinal zones have been defined.

Referee's comment:
-Does ARHGAP11B overexpression in wildtype human cause NPC over-proliferation?

Authors' response:
No. We have overexpressed ARHGAP11B alone, and also ARHGAP11A alone as well as both together, in wildtype human organoids, and in none of these three conditions did we observe a significant difference in basal progenitor levels compared to mock-transfected human organoids. We have included this finding in the revised manuscript (see lines 472-474 and Appendix Fig S4).

Referee's comment:
-Does ARHGAP11B also cause increased apical progenitor proliferation?

Authors' response:
No. Previous studies from one of our labs have established that *ARHGAP11B* expression in embryonic mouse, developing ferret and fetal marmoset neocortex does not increase apical progenitor proliferation (Florio et al. 2015 *Science* 347, 1465; Florio et al. 2016 *Sci. Adv.* 2, e160194; Kalebic et al. 2018 *eLife* 7, e41241; Heide et al. 2020 *Science* 369, 546; Xing et al. 2021 *EMBO J.* 40, e107093).

Referee's comment:
-How were the timepoints selected for analysis and are these comparable between species? Also, there could be more methodological details on how the authors ensure that the control and ARHGAP11B electroporation are in ventricles of similar character to start, and how does the SVZ size vary between ventricles and over time in human and chimpanzee organoids?

Authors' response:
The time points for analysis (see Figure EV1A) were chosen based on our knowledge of cerebral organoid development over time and the time courses of VZ formation, SVZ formation, deep-layer neuron generation and upper-layer neuron generation. In our experience, these time courses are roughly comparable between human and chimpanzee cerebral organoids (in contrast to macaque organoids, consistent with a report from the

Livesey lab). The time intervals between electroporation and analysis were chosen based on our knowledge of cell lineages (apical progenitors to basal progenitors to neurons) and of progenitor cell cycle length, which has allowed us to predict when *ARHGAP11B* transfection can be expected to affect the various cell type populations under study.

The ventricles used for the comparative analyses had similar morphology with regard to the VZ (packing density of nuclei, radial organization), SVZ and neuronal layers, and exhibited a sufficiently high and roughly equal number of electroporated cells.

Consistent with previous findings (Mora-Bermúdez et al. 2016 *eLife* 5, e18683), we did not observe obvious differences in SVZ size between human and chimpanzee organoids.

We have clarified all these issues in the revised manuscript (see lines 165-168 and 773-777).

Referee's comment:
-Similarly, on the timepoint selection, Line 308 states implies that the first neurons are produced at day 51 in organoids, but other studies have reported neurons by 3 to 5 weeks or earlier.

Authors' response:
There appears to be a misunderstanding here. The Reviewer is correct, of course, that the first neurons generated in organoids are not produced only at day 51, but earlier. What we meant to say is that we investigated whether the neurons generated on day 51 are of the deep-layer neuron type, which is the first class of cortical neurons produced. We have clarified this point in the revised manuscript and have re-phrased the relevant section of the text to avoid any misunderstanding (see lines 311-319).

Referee's comment:
-For the neuron fate changes, could ectopic ARHGAP11B expression in neurons influence their survival/identity (for example contributing to the reduction of deep layer neurons, as the earliest born neurons may inherit a high plasmid copy number for a gene not normally expressed), but then plasmid dilution could cause less influence on upper layer neuron production?

Authors' response:
The scenario put forward by the Reviewer is theoretically possible, but in our opinion unlikely, for the following reasons. First, all previous studies from our and other labs have shown that ARHGAP11B, consistent with its action in mitochondria to increase glutaminolysis, only affects cycling cells, not post-mitotic cells such as neurons. Second, we did not observe any increase in apoptosis in the neuronal layers upon *ARHGAP11B* expression. We have discussed this scenario in the revised manuscript (see lines 355-363).

Referee's comment:
-Clarify whether ARHGAP11A220 also interferes with ARHGAP11A?

Authors' response:
In a previous study from one of our labs (Namba et al. 2020 *Neuron* 105, 867), it was shown that ARHGAP11A220 (a truncated version of ARHGAP11A), similar to ARHGAP11B and

in contrast to full-length ARHGAP11A, localizes to mitochondria and not (like ARHGAP11A) to the nucleus. We have now mentioned this in the revised manuscript (see lines 378-384). In light of this spatially distinct subcellular localization of ARHGAP11A and ARHGAP11A220, we find it unlikely that ARHGAP11A220 also interferes with the function of ARHGAP11A.

Referee's comment:
In 4B, the analysis of the fraction of BrdU+ GFP cells between human and chimp, lacks sufficient biological replication (and also does not account for potential timing differences between species) for claims about species level differences in basal progenitor number - this should be acknowledged and the claims must focus on the within species aspect for which there is substantial technical replication of the conditions. For example, line 393 "Interestingly, this reduction brought the level of these BPs down to that observed in chimpanzee" must be clarified to in this comparison of one human and one chimp individual or removed.

Authors' response:
In line with the Reviewer's comment, in the revised version, we have clarified that the data shown in Figure 4B, as well as those shown in Figure 4D, are from multiple human and chimpanzee organoids that however were grown from only one human iPSC line and only one chimpanzee iPSC line, respectively (see lines 406-409 and legend of Figure 4).

Referee's comment:
In 4b, was this chimpanzee phenotype from the dominant negative harder to detect simply because there were fewer dividing basal progenitors at this stage? And would this reduction in basal progenitors be true across organoids from other chimpanzee individuals as a species-specific organoid phenotype or just in the tested individual? My understanding is that the organoid field has not extensively measured variation in features of histogenesis and basal progenitor number even across human individuals.

Authors' response:
No, we do not think that the lack of an ARHGAP11A220 phenotype in the chimpanzee organoids reflects a potential difficulty in detecting such a phenotype because of the lower level of basal progenitors in chimpanzee than human, for the following reasons. First, the apparently low levels of basal progenitors in chimpanzee organoids reflect more the mode of expressing the data (proportion of GFP+ SVZ cells that are BrdU+) than the absolute abundance of basal progenitors. As can be seen from Figure 4A, there are plenty of BrdU+ basal progenitors in the chimpanzee. Second, even if the level of cycling basal progenitors in chimpanzee organoids were higher, we believe that ARHGAP11A220 would still not cause their reduction, as ARHGAP11A220 was previously shown to not reduce the basal progenitor level in another model system that like the chimpanzee lacks *ARHGAP11B*, that is, the mouse neocortex (Namba et al. 2020 *Neuron* 105, 867).

The question of whether the lower level of basal progenitors in chimpanzee organoids as compared to human organoids is only seen in the two iPSC lines used to grow the organoids (human: SC102A-1, chimpanzee: Sandra A), or would be observed also in chimpanzee vs. human organoids grown from other iPSC lines, is difficult to answer as there are very few chimpanzee iPSC lines available. In a previous study involving one of our labs (Mora-Bermúdez et al. 2016 *eLife* 5, e18683), no major difference in organoid basal progenitor

levels between the two chimpanzee iPSC lines used (PR818-5, Sandra A) was noticed. But the Reviewer is entirely correct that the organoid field has not extensively measured variation in features of histogenesis and basal progenitor levels in organoids grown from various human and chimpanzee iPSC lines. In light of this, it is certainly wise to emphasize that the data shown in Figure 4B only reflect one human iPSC line and only one chimpanzee iPSC line, which we have done in the revised manuscript (see lines 406-409, legend of Figure 4 and also our response above).

Referee's comment:
-For Clone 16, is it worth doing Southern blots to confirm no additional integration, or sequencing to assess off-target effect, or is the point that Clone 16 provides a line for isogenic experiments testing overexpression

Authors' response:
As stated in one of our responses above, we have analyzed an independent double knockout clone from another genetic background (clone j) in addition to clone 16. We have examined both double knockout clones for potential off-target integrations of the editing, and did not find any integration that is identical between the two ARHGAP11A and ARHGAP11B double-knockout clones indicating that any effect observed for both *ARHGAP11A* and *ARHGAP11B* double knockout clones would not be due to an off-target integration but rather specific to the double knockout (see lines 449-454 and Table EV1).

Referee's comment:
Other comments

-"However, the extent of ARHGAP11B's contribution to this expansion during hominid evolution is unknown" is a major question that is introduced quantitatively, but only a qualitative answer is provided. I don't think a quantitative answer is possible in this study, because with one human and one chimp line, it is not possible to measure overall species differences in basal progenitor number.

Authors' response:
Regarding this comment, we respectfully disagree with the Reviewer. The massive decrease in basal progenitor levels (which is a quantitative result) was observed with two independent approaches involving two completely different human clones, (i) the inhibition of ARHGAP11B function by overexpression of the dominant-negative ARHGAP11A220 in organoids grown from the human iPSC line SC102A-1 (Figure 4), and (ii) the specific *ARHGAP11B* knockout via the selective *ARHGAP11A* rescue in organoids grown from the double knockout clone 16 derived from the human iPSC line GM08680 (Figure 5). The results of the specific *ARHGAP11B* knockout (Figure 5) could in the revised version of the manuscript also be confirmed on an additional double knockout clone (clone j) generated from iPSCs of another genetic background. We think that, collectively, these two sets of data, including the additional data of clone j, do make the point that ARHGAP11B exerts a major role in maintaining the elevated basal progenitor levels in human cerebral organoids, which in turn implies that ARHGAP11B was a major contributor to human neocortex expansion during hominid evolution.

Referee's comment:
-Line 57 - could also cite earlier Sasai lab studies, such as Eiraku et al., 2008

Authors' response:
The Reviewer is correct. We now also included more citations of the seminal work from the Sasai lab.

Referee's comment:
-Lines 251 and 282 repeat almost identical language

Authors' response:
In the revised manuscript, we have rephrased the text in the two paragraphs concerned to avoid duplication and redundancy.

Referee #2:

Referee's comment:

This study by the Huttner lab investigates the effect of overexpressing the human-specific gene ARHGAP11B in iPSC-derived cerebral organoids from human and chimpanzee. They replicate the same findings already reported by the same group in mouse, ferret and marmoset, now in chimpanzee organoids, where this overexpression promotes the generation of cortical basal progenitors, a key step in the complex developmental program driving the expansion of the cerebral cortex. The study has some points of novelty, such as testing ARHGAP11B in chimp organoids or performing loss-of-function experiments in human organoids, but these are minor and overall the study brings very limited, if any, conceptual advance to this important question. There are also a number of issues that the authors should address in their manuscript:

Authors' response:

We respectfully disagree with this rather negative evaluation by this Reviewer, who in our humble opinion appears to underrate the novelty of our findings. First, we do not just *"replicate"* previous findings; rather, our study is the first to show a gain-of-function effect of the human-specific gene *ARHGAP11B* in cerebral organoids, a model system that becomes increasingly important for studies of cortical development, and is the first to show this for the chimpanzee, our closest living relative. This set of findings demonstrates that ARHGAP11B is sufficient to increase basal progenitors, the progenitors with a key role in neocortex expansion, to the elevated level characteristic of humans. Second, our study is the first to demonstrate the loss-of-function effect for ARHGAP11B using two independent approaches, (i) the inhibition of ARHGAP11B protein function by dominant-negative ARHGAP11A220, and (ii) the knockout of the *ARHGAP11B* gene. Both approaches demonstrate that *ARHGAP11B* is essential to ensure the elevated BP levels characteristic of human neocortical tissue and imply, for the first time, that this human-specific gene was a major contributor to the evolutionary expansion of the human neocortex. These findings are therefore not just *"minor points of novelty"* and do not constitute merely a *"very limited conceptual advance"*, but rather the opposite.

As a side note, we would like to politely point out that our study is not just *"by the Huttner lab"*, but actually involves three labs, the new lab of Michael Heide at the German Primate Center in Göttingen, Julia Ladewig's lab at the Central Institute of Mental Health in Mannheim, and Wieland Huttner's lab at the MPI-CBG in Dresden where Michael Heide started his work.

Referee's comment:

Line 106 - "Examining human-specific genes [our results] provide corroborating evidence in support of their presumptive role in neocortical development during human evolution, but also open up new avenues to dissect their mechanism of action." This study does not really open new avenues to dissect the mechanism of action of ARHGAP11B, but only that ARHGAP11A220 is a loss-of-function.

Authors' response:

There appears to be a misunderstanding here. We mean that in general the study of human-specific genes in human and chimpanzee organoids will open up new avenues to dissect, in

the future, their mechanism of action. This would apply not only to the human-specific genes *ARHGAP11B* and *NOTCH2NL*, where previous studies have already provided some insight into the mechanism of action but further investigation is necessary, but also to other human-specific genes whose mechanism of action is essentially unknown. We have clarified this point in the revised manuscript (see lines 99-102).

Referee's comment:
Line 164 - Introduction is long and circular around known and unknown the functions of ARHGAP11B, and open questions, but in the end it is very unclear what the aim of the study really is. The authors should improve or establish a hierarchy in their ideas exposed in relation to the importance of the open questions, much necessary to focalize the attention of readers.

Authors' response:
We agree with the Reviewer that the flow of the Introduction could be improved and could be more to the point, and that some of its paragraphs could, and should, be condensed. In the revised manuscript, we have provided a shortened and more focused Introduction.

Referee's comment:
Line 263 - "bRG are thought to be key for mammalian neocortex evolution and to drive expansion and folding of the human neocortex" - Here, the authors should cite more recent and comprehensive reviews on expansion and folding of the neocortex, such as for example the nice review by Borrell and colleagues in Nat Rev Neurosci, 2019.

Authors' response:
This criticism by the Reviewer is correct. We apologize for not having cited the review by Llinares-Benadero & Borrell in *Nature Reviews Neuroscience* 2019, which indeed is an outstanding review on cortical folding. We now have cited this important review in the revised manuscript, as well as other recent insightful reviews by Borrell and colleagues.

Referee's comment:
In spite of the use of Hopx as a marker of bRG in several studies, mounting evidence in the field shows that this is really not a bRG marker. It is in fact striking that the authors claim the utility of this as bRG marker, since the stains in Figure 2 show many aRGs in VZ positive for Hopx. Significantly, in addition to Hopx not being a marker of bRG, the authors are not showing any of the typical signs of bRG for which these are distinct from other BPs: basal radial process and lack of apical process.

Authors' response:
The Reviewer is correct that HOPX is also expressed in aRG in the VZ. We integrated in the revised version on the manuscript the information that HOPX is a radial glial marker. However, as was indicated by the labeling of the ordinates in panels B and D of Figure 2 and in Figure 2 legend, we had confined our quantification to the SVZ, where HOPX is preferentially expressed in bRG rather than basal intermediate progenitors (bIPs). We have better explained this in the revised manuscript (see lines 265-269).

Due to the overall low abundance of mitotic bRG in chimpanzee organoids, we did not perform phospho-vimentin immunofluorescence, the "classical" way to distinguish mitotic bRG from mitotic bIPs, as correctly noted by the Reviewer.

Referee's comment:
Line 304 - "We conclude that the increased abundance of cycling BPs observed after a 10-day period of expression of ARHGAP11B in chimpanzee cerebral organoids reflects an increased generation of BPs from BPs, at the expense of the generation of cortical neurons from BPs" - The absence of neuron marker expression does not demonstrate progenitor identity, so this conclusion is not supported by the observations. Instead, the authors should demonstrate increased cell cycle re-entry, or increased proportion of Ki67+ cells among GFP+ cells.

Authors' response:
In line with the Reviewer's request *"...the authors should demonstrate... increased proportion of Ki67+ cells among GFP+ cells"*, Figure EV3 of our original manuscript actually did show an increase in the proportion of GFP+ cells in the SVZ that are PCNA+ (equivalent to Ki67+) 10 days after electroporation. As this result could be missed by readers (as it apparently was by the Reviewer), we have better emphasized this important point in the revised manuscript (see lines 274-284).

Referee's comment:
Line 321 - "the first neurons generated by BPs in chimpanzee cerebral organoids, the generation of which is reduced upon ARHGAP11B expression due to the increased generation of BPs, are of the deep-layer type" - Was there an increase in Satb2+ neurons at this early time-point? Maybe ARHGAP11B promotes a shift from deep to superficial layer neuron type, without an actual delay in neurogenesis. Otherwise, this would mean an abnormal deficit in deep layer neurons, which is not observed in human cortex. Also, the hypothesis that delayed neurogenesis would altogether skip deep-layer neurogenesis is flawed, as delayed developmental programs usually resume following all steps of neurogenesis, even if these are overall shortened.

Authors' response:
In answer to the Reviewer's question: At the early time point of analysis (4 days after electroporation), only a minimal number of the GFP+ cells were Satb2+ (Appendix Fig S3), and we did not detect an increase in the proportion of GFP+ cells that were Satb2+. We have added this finding to the revised manuscript (see lines 181-214 and 331-334). This finding implies that the scenario put forward by the Reviewer *"Maybe ARHGAP11B promotes a shift from deep to superficial layer neuron type..."* does not appear to be the case.

We respectfully disagree with the Reviewer's conclusion that our finding would imply *"...an abnormal deficit in deep layer neurons..."*, as a delay in the onset of cortical neurogenesis (without any deficit in deep-layer neurons) due to a prolongation of cortical progenitor proliferation is a characteristic hallmark of more highly developed brains, including human.

Finally, regarding the Reviewer's comment *"Also, the hypothesis that delayed neurogenesis would altogether skip deep-layer neurogenesis is flawed..."*, we never proposed such a hypothesis.

Referee's comment:
Line 389 - "TBR2, a marker of BPs (Englund et al., 2005, Sessa et al., 2008) that in fetal human neocortex is typically expressed in the basal intermediate progenitor (bIP) subpopulation of BPs (Hevner, 2019, Mihalas et al., 2016)." - None of these two articles show that human neocortex bIPs express Tbr2, nor that it is a marker for them. Contrarily, multiple other studies show that bRGs in non-mouse species co-express Pax6 and Tbr2. Why the abundance of bRGs was not studied in these experiments?

Authors' response:
We respectfully disagree with the Reviewer's statement that *"...multiple other studies show that bRGs in non-mouse species co-express Pax6 and Tbr2."* In our experience, bRG in fetal human neocortex express PAX6 but do not express TBR2 (see Figure 3g in Fietz et al. 2010 *Nature Neurosci.* 13, 690-699). In the revised manuscript, we have referred to this original publication (rather than to Hevner, 2019 and Mihalas et al., 2016). The lack of TBR2 expression in human bRG in turn implies that the TBR2 expression observed in the fetal human SVZ is a typical feature of the other class of BPs, i.e. basal intermediate progenitors (bIPs), as stated in our manuscript.

The effect of loss of ARHGAP11B function on bRG levels was studied upon *ARHGAP11B* knockout (see Figure 5B).

Referee's comment:
Line 439 - "we investigated whether the ARHGAP11B knockout resulted in a decreased BP abundance in the human forebrain organoids. Immunohistochemistry for the bRG marker PTPRZ1 (Pollen et al., 2015) (Figure 5A)" - Why the authors used here a different bRG marker? Why did they wait for 10 days after electroporation instead of 4 days, as above? Were control organoids isogenic with the mutants?

Authors' response:
We used the bRG marker PTPRZ1 in the analysis of the SVZ upon *ARHGAP11B* knockout (Figure 5) to complement the analysis of the inhibition of ARHGAP11B function by dominant-negative ARHGAP11A220 (Figure 4), where TBR2 was used as marker, which – as explained in our response above – predominantly identifies the bIPs among the human BPs.

We analyzed the SVZ upon *ARHGAP11B* knockout 10 days after electroporation of human organoids because we knew from the overexpression of *ARHGAP11B* in chimpanzee organoids (Figure 2) that the effect on bRG levels is stronger after 10 days than 4 days. We have explained this in the revised manuscript (see lines 463-467).

Yes, the control organoids were isogenic with the mutant organoids.

Referee's comment:
Line 447 - "Together with the results of the dominant-negative ARHGAP11A220 in human cerebral organoids (Figure 4), these data demonstrate that ARHGAP11B is required to maintain the elevated levels of cycling BPs that are characteristic of human cerebral cortex tissue." - Prior to rescue, were double-KO organoids different in any way from wild-type isogenic organoids? Did the absence of ARHGAP11B affect only after GFP electroporation?

Authors' response:

Yes, the bRG level in the double-KO organoids prior to rescue was different from that of wild-type isogenic organoids. The reason why we did not show data for the double knockout prior to rescue is that an interpretation of these data would be difficult, as it would be unclear to which extent a change in the level of basal radial glia would be due to loss of *ARHGAP11A* or *ARHGAP11B* or both.

Referee's comment:
Results in Figure 5B for ARHGAP11A KO are apparently bimodally distributed, which is not the case for ARHGAP11B. What is the cause of that? Different organoid batches? Does this mean partial penetrance of the phenotype, where half of AKOs were also affected? How is this interpreted?

Authors' response:
While we agree with the Reviewer that the distribution of the *ARHGAP11A* KO data seems to be bimodal, we note all the data points are in the same range as in the control. We do not think that this calls for any deep interpretation, as it may simply reflect experimental variation. For the revised version of our manuscript, we also grew organoids from an independent double knockout clone and then repeat the various rescue experiments, similar to Figure 5 of our original manuscript. Here we do not see any bimodal distribution of the *ARHGAP11A* KO data supporting the notion that the data simply reflect experimental variations (see new Figure 5B).

Referee's comment:
Discussion, line 485 - "ARHGAP11B alone is capable of increasing the abundance of cycling BPs to a human-like level and hence of providing a crucial basis for the evolutionary expansion of the human neocortex." - Did ARHGAP11B function ultimately influence the amount of cortical neurogenesis? Were human organoids smaller when blocking ARHGAP11B function, or chimp organoids larger when OE this gene? That is, were increased BPs functional? Neurogenic?

Authors' response:
As organoid electroporation is limited to only some ventricle-like structures of an organoid, and only to parts of a targeted ventricle-like structure (see Figure EV1B), the potential effects of ARHGAP11B loss-of-function and gain-of function on organoid size cannot be examined in a meaningful way by the present approaches.

Referee's comment:
Minor point: The limits of SVZ need to be indicated in the figures across the manuscript, as well as GFP+ cells positive for the studied markers.

Authors' response:
We thank the Reviewer for this suggestion. We have added arrowheads pointing to the different combinations of positive cells, and have indicated the boundaries between VZ and SVZ/CP.

1st Revision - Editorial Decision 10th Aug 2022

Dear Dr. Heide,

Thank you for the submission of your revised manuscript. We have now received the enclosed report from referee 1, who also assessed your response to referee 2's concerns. Referee 1 still has a few more minor suggestions that I would like you to address and incorporate before we can proceed with the official acceptance of your manuscript.

A few editorial requests will also need to be addressed:

- Your manuscript has 5 main figures but is layed out as a full article. Please either add one more main figure or combine the results and discussion sections and shorten the full manuscript text to below 27.000 characters (including spaces but excluding materials & methods and references).

- Please update the conflict of interest subheading to "Disclosure and Competing Interest Statement"

- Fig 3A is called out before Fig 2, please correct.

- Tables EV2 & EV3 callouts are missing, please add.

- Tables EV2 and EV3 are in .bed format which is not a format supported by EMBO Press. Can you please upload the tables as excel or word file?

- The Appendix table of content is missing page numbers, please add. The nomenclature needs to be corrected to "Appendix Figure S#".

- Please check whether the link to your deposited DNA sequencing data in the Data Availability Section is correct and functional.

I would like to suggest a few minor changes to the abstract that needs to be written in present tense:

The human-specific gene ARHGAP11B has been implicated in human neocortex expansion. However, the extent of ARHGAP11B's contribution to this expansion during hominid evolution is unknown. Here we address this issue by genetic manipulation of ARHGAP11B levels and function in chimpanzee and human cerebral organoids. ARHGAP11B expression in chimpanzee cerebral organoids doubles basal progenitor levels, the class of cortical progenitors with a key role in neocortex expansion. Conversely, interference with ARHGAP11B's function in human cerebral organoids decreases basal progenitors down to the chimpanzee level. Moreover, ARHGAP11A or ARHGAP11B rescue experiments in ARHGAP11A plus ARHGAP11B double-knockout human forebrain organoids indicate that lack of ARHGAP11B, but not of ARHGAP11A, decreases the abundance of basal radial glia - the basal progenitor type thought to be of particular relevance for neocortex expansion. Taken together, our findings demonstrate that ARHGAP11B is necessary and sufficient to ensure the elevated basal progenitor levels that characterize the fetal human neocortex, suggesting that this human-specific gene was a major contributor to neocortex expansion during human evolution.

The synopsis image you sent is very nice, but the text is not readable at the final image size of 550x340 pixels. I attach the final image to this email for your information. Can you please send us a new synopsis image that is may be less crowded and with a larger font?

I look forward to seeing a final version of your manuscript as soon as possible. Please use this link to submit your revision:
https://embor.msubmit.net/cgi-bin/main.plex

Best regards,
Esther

Esther Schnapp, PhD
Senior Editor
EMBO reports

Referee #1:

This study provides a crucial addition to our understanding of the role of ARHGAP11B in brain evolution by demonstrating sufficiency in chimpanzee and necessity in human developmental models for the role of ARHGAP11B in basal progenitor

proliferation. My concerns have largely been addressed and I congratulate the authors on improving an already strong study.

The authors have added text qualifications to address my major concern about non-physiological levels of ARHGAP11B caused by the electroporation method and strong promoter that would shrink over time due to plasmid dilution. These text qualifications are sufficient, as I appreciate that quantitative experimental comparison of ARHGAP11B levels in electroporated cells to endogenous levels would be challenging. As a subtle point, I would make this technical distinction slightly more clear in the abstract or bullet points because the method of modeling human-specific mutations matters for interpretation of the magnitude of the effect. For example, line 33 could say "over-expression" instead of just "expression."

The replication of results from Clone 16 in the independent Clone J provides strong validation and I applaud the authors for executing these difficult experiments. However, I am still not fully understanding the decision not to show the data from the double knockout. The stated explanation that "it would be unclear to which extent a reduction in the level of basal radial glia would be due to loss of ARHGAP11A or ARHGAP11B or both" is exactly why the individual rescue experiments were also performed, but the double knockout still strikes me as useful (and commonly used control) for interpretation. Maybe I am missing something, but if the authors collected that data, I would suggest at least sharing it in the supplement.

The data that ARHGAP11B overexpression does not have strong effects in human organoids (EV4) adds further confidence to the authors' interpretations.

I also went through reviewer 2's comments in detail during my re-review, and I feel that the comments have largely been addressed.

1. the limited conceptual advance - I think reviewer 2 overstated this - showing necessity in human bRG cells was important and missing from previous studies, and I also highlighted this novelty in my initial review
2. I agree that the study does not point to molecular mechanisms - the authors seem to be satisfied with the glutaminolysis model, which I have not closely read (though I can imagine future experiments to test if that is the mechanism of action), but mechanism is beyond the scope of demonstrating the role in human/chimp context
3. the introduction has now been condensed
4. I agree with the authors that bRG cells do not express TBR2 and the authors have added various new stains requested
5. Reviewer 2 also asked "Prior to rescue, were double-KO organoids different in any way from wild-type isogenic organoids?" - and the authors dodged showing this data, stating that it would be difficult to interpret. I find this point frustrating as I mentioned in my initial and second review. The double KO is a natural control for the rescue of individual genes, and if the authors already collected this data, why not present it - at least in a table or the supplement, especially if both reviewers asked for it? The authors argued for a "clean" experiment, but I think omitting it may make for a "clean" figure. Nonetheless, the authors have used multiple orthogonal methods to support their conclusions, and characterizing a whole second clone in revision was a great addition, so I don't want to get hung up on this point.

Response to Editor

Editor's Comment:
A few editorial requests will also need to be addressed:

Authors' Response:
Done, as described point-by-point below.

Editor's Comment:
- Your manuscript has 5 main figures but is layed out as a full article. Please either add one more main figure or combine the results and discussion sections and shorten the full manuscript text to below 27.000 characters (including spaces but excluding materials & methods and references).

Authors' Response:
We decided to go for the first option and have now added the previous Fig EV1 as the new Fig 1.
Previous Figs 1-5 are now Figs 2-6.
Previous Figs EV2-5 are now Figs EV1-4.

Editor's Comment:
- Please update the conflict of interest subheading to "Disclosure and Competing Interest Statement".

Authors' Response:
Done.

Editor's Comment:
- Fig 3A is called out before Fig 2, please correct.

Authors' Response:
Corrected.

Editor's Comment:
- Tables EV2 & EV3 callouts are missing, please add.

Authors' Response:
Added.

Editor's Comment:
- Tables EV2 and EV3 are in .bed format which is not a format supported by EMBO

Press. Can you please upload the tables as excel or word file?

Authors' Response:
Yes, we will do that.

Editor's Comment:
- The Appendix table of content is missing page numbers, please add. The nomenclature needs to be corrected to "Appendix Figure S#".

Authors' Response:
Page numbers have now been added to the Appendix table of content.
The nomenclature of all Appendix Figures is now as requested.

Editor's Comment:
- Please check whether the link to your deposited DNA sequencing data in the Data Availability Section is correct and functional.

Authors' Response:
The link to our deposited DNA sequencing data in the Data Availability Section is correct and functional.

Editor's Comment:
I would like to suggest a few minor changes to the abstract that needs to be written in present tense:
The human-specific gene ARHGAP11B has been implicated in human neocortex expansion. However, the extent of ARHGAP11B's contribution to this expansion during hominid evolution is unknown. Here we address this issue by genetic manipulation of ARHGAP11B levels and function in chimpanzee and human cerebral organoids. ARHGAP11B expression in chimpanzee cerebral organoids doubles basal progenitor levels, the class of cortical progenitors with a key role in neocortex expansion. Conversely, interference with ARHGAP11B's function in human cerebral organoids decreases basal progenitors down to the chimpanzee level. Moreover, ARHGAP11A or ARHGAP11B rescue experiments in ARHGAP11A plus ARHGAP11B double-knockout human forebrain organoids indicate that lack of ARHGAP11B, but not of ARHGAP11A, decreases the abundance of basal radial glia - the basal progenitor type thought to be of particular relevance for neocortex expansion. Taken together, our findings demonstrate that ARHGAP11B is necessary and sufficient to ensure the elevated basal progenitor levels that characterize the fetal human neocortex, suggesting that this human-specific gene was a major contributor to neocortex expansion during human evolution.

Authors' Response:
All your changes to the abstract have been implemented.

Editor's Comment:
The synopsis image you sent is very nice, but the text is not readable at the final image size of 550x340 pixels. I attach the final image to this email for your information. Can you please send us a new synopsis image that is may be less crowded and with a larger font?

Authors' Response:
As requested, we have prepared a new synopsis image that is less crowded and illustrates the findings of our study in a more reader-friendly way.

Response to Reviewers

Reviewer #1

<u>*Reviewer's Comment:*</u>
This study provides a crucial addition to our understanding of the role of ARHGAP11B in brain evolution by demonstrating sufficiency in chimpanzee and necessity in human developmental models for the role of ARHGAP11B in basal progenitor proliferation. My concerns have largely been addressed and I congratulate the authors on improving an already strong study.

<u>Authors' Response:</u>
We very much appreciate the Reviewer's positive evaluation and would like to explicitly thank the Reviewer for the constructive comments that have greatly helped us to improve our study.

<u>*Reviewer's Comment:*</u>
The authors have added text qualifications to address my major concern about non-physiological levels of ARHGAP11B caused by the electroporation method and strong promoter that would shrink over time due to plasmid dilution. These text qualifications are sufficient, as I appreciate that quantitative experimental comparison of ARHGAP11B levels in electroporated cells to endogenous levels would be challenging. As a subtle point, I would make this technical distinction slightly more clear in the abstract or bullet points because the method of modeling human-specific mutations matters for interpretation of the magnitude of the effect. For example, line 33 could say "over-expression" instead of just "expression."

<u>Authors' Response:</u>
We appreciate that the Reviewer finds our text qualifications sufficient.

The Reviewer has raised a fair point regarding the use of the term "expression", with which we agree. In the revised manuscript, we have therefore clarified this point more and have used the term "overexpression" in many places where we felt this was relevant, in particular in the context of Fig. 6 (double-KO of *ARHGAP11A* plus *ARHGAP11B* followed by ARHGAP11A and/or ARHGAP11B rescue). However, in the abstract we prefer to keep the term "expression", as we wish to make the basic point that we express ARHGAP11B in the chimpanzee where it normally does not exist.

<u>*Reviewer's Comment:*</u>
The replication of results from Clone 16 in the independent Clone J provides strong validation and I applaud the authors for executing these difficult experiments. However, I am still not fully understanding the decision not to show the data from the double knockout. The stated explanation that "it would be unclear to which extent a reduction in the level of basal radial glia would be due to loss of ARHGAP11A or ARHGAP11B or both" is exactly why the individual rescue experiments were also performed, but the

double knockout still strikes me as useful (and commonly used control) for interpretation. Maybe I am missing something, but if the authors collected that data, I would suggest at least sharing it in the supplement.

Authors' Response:
We appreciate that the Reviewer finds that the addition of the Clone J data provides strong validation.

As to showing the data on the bRG levels in the *ARHGAP11A* plus *ARHGAP11B* double-knockout organoids, we now show these data in comparison to organoids generated from control iPSCs, for both clone 16 and clone J, in the new Appendix Figure S5A.

The data suggest that in addition to the established action of ARHGAP11B to increase bRG levels, there apparently is an opposite action of ARHGAP11A to reduce cNPC delamination and, consequently, bRG levels. This presumptive action of ARHGAP11A in the VZ is lacking in the *ARHGAP11A* plus *ARHGAP11B* double-knockout organoids, which is why the bRG level is higher than in organoids generated from control iPSCs. Support for this notion is provided in panel B of the new Appendix Figure S5.

Panel B of the new Appendix Figure S5 shows that when ARHGAP11A action in the *ARHGAP11A* plus *ARHGAP11B* double-knockout human forebrain organoids is restored upon electroporation of the *ARHGAP11A* expression plasmid, bRG levels are back down to normal. As ARHGAP11B action in the *ARHGAP11A* plus *ARHGAP11B* double-knockout human forebrain organoids is still lacking, bRG levels remain low.

Reviewer's Comment:
The data that ARHGAP11B overexpression does not have strong effects in human organoids (EV4) adds further confidence to the authors' interpretations.

Authors' Response:
We thank the Reviewer for this positive statement.

Reviewer's Comment:
I also went through reviewer 2's comments in detail during my re-review, and I feel that the comments have largely been addressed.

Authors' Response:
We sincerely thank the Reviewer for this effort.

Reviewer's Comment:
1. the limited conceptual advance - I think reviewer 2 overstated this - showing necessity in human bRG cells was important and missing from previous studies, and I also highlighted this novelty in my initial review.

Authors' Response:
We thank the Reviewer for making this point.

Reviewer's Comment:
2. I agree that the study does not point to molecular mechanisms - the authors seem to be satisfied with the glutaminolysis model, which I have not closely read (though I can imagine future experiments to test if that is the mechanism of action), but mechanism is beyond the scope of demonstrating the role in human/chimp context.

Authors' Response:
We thank the Reviewer for this assessment.

Reviewer's Comment:
3. the introduction has now been condensed.

Authors' Response:
We thank the Reviewer for this statement.

Reviewer's Comment:
4. I agree with the authors that bRG cells do not express TBR2 and the authors have added various new stains requested.

Authors' Response:
We thank the Reviewer for this statement.

Reviewer's Comment:
5. Reviewer 2 also asked "Prior to rescue, were double-KO organoids different in any way from wild-type isogenic organoids?" - and the authors dodged showing this data, stating that it would be difficult to interpret. I find this point frustrating as I mentioned in my initial and second review. The double KO is a natural control for the rescue of individual genes, and if the authors already collected this data, why not present it - at least in a table or the supplement, especially if both reviewers asked for it? The authors argued for a "clean" experiment, but I think omitting it may make for a "clean" figure. Nonetheless, the authors have used multiple orthogonal methods to support their conclusions, and characterizing a whole second clone in revision was a great addition, so I don't want to get hung up on this point.

Authors' Response:
As requested by both Reviewers and as described in detail in our response to Reviewer #1 above, the new Appendix Figure S5A now shows the bRG levels in the double-KO organoids in comparison to that of wild-type isogenic organoids, and the legend to this figure panel offers the explanation that in addition to the established action of

ARHGAP11B to increase bRG levels, there apparently is an opposite action of ARHGAP11A to reduce cNPC delamination and, consequently, bRG levels. This presumptive action of ARHGAP11A in the VZ is lacking in the *ARHGAP11A* plus *ARHGAP11B* double-knockout organoids, which is why the bRG level is higher than in organoids generated from control iPSCs. Support for this notion is provided in panel B of the new Appendix Figure S5.

Panel B of the new Appendix Figure S5 shows that when ARHGAP11A action in the *ARHGAP11A* plus *ARHGAP11B* double-knockout human forebrain organoids is restored upon electroporation of the *ARHGAP11A* expression plasmid, bRG levels are back down to normal. As ARHGAP11B action in the *ARHGAP11A* plus *ARHGAP11B* double-knockout human forebrain organoids is still lacking, bRG levels remain low.

Dr. Michael Heide
Deutsches Primatenzentrum
Kellnerweg 4
Göttingen, Lower Saxony 37033
Germany

Dear Dr. Heide,

I am very pleased to accept your manuscript for publication in the next available issue of EMBO reports. Thank you for your contribution to our journal.

Please note that under the DEAL agreement of German scientific institutions with our publisher Wiley, you could be eligible for publication of your article in the open access format in a way that is free of charge for the authors. Please contact either the administration at your institution or our publishers at Wiley (emboreports@wiley.com) for further questions.

At the end of this email I include important information about how to proceed. Please ensure that you take the time to read the information and complete and return the necessary forms to allow us to publish your manuscript as quickly as possible.

As part of the EMBO publication's Transparent Editorial Process, EMBO reports publishes online a Review Process File to accompany accepted manuscripts. As you are aware, this File will be published in conjunction with your paper and will include the referee reports, your point-by-point response and all pertinent correspondence relating to the manuscript.

If you do NOT want this File to be published, please inform the editorial office within 2 days, if you have not done so already, otherwise the File will be published by default [contact: emboreports@embo.org]. If you do opt out, the Review Process File link will point to the following statement: "No Review Process File is available with this article, as the authors have chosen not to make the review process public in this case." Please note that the author checklist will still be published even if you opt out of the transparent process.

Thank you again for your contribution to EMBO reports and congratulations on a successful publication. Please consider us again in the future for your most exciting work.

Best regards,
Esther

Esther Schnapp, PhD
Senior Editor
EMBO reports

****

THINGS TO DO NOW:

Once your article has been received by Wiley for production, the corresponding author will receive an email from Wiley's Author Services system which will ask them to log in and will present them with the appropriate license for completion.

You will receive proofs by e-mail approximately 2-3 weeks after all relevant files have been sent to our Production Office; you should return your corrections within 2 days of receiving the proofs.

Please inform us if there is likely to be any difficulty in reaching you at the above address at that time. Failure to meet our deadlines may result in a delay of publication, or publication without your corrections.

All further communications concerning your paper should quote reference number EMBOR-2022-54728V3 and be addressed to emboreports@wiley.com.

Should you be planning a Press Release on your article, please get in contact with emboreports@wiley.com as early as possible, in order to coordinate publication and release dates.

EMBO Press Author Checklist

Corresponding Author Name: Wieland B. Huttner, Julia Ladewig, Michael Heide
Journal Submitted to: EMBO Reports
Manuscript Number: EMBOR-2022-54728

USEFUL LINKS FOR COMPLETING THIS FORM
The EMBO Journal - Author Guidelines
EMBO Reports - Author Guidelines
Molecular Systems Biology - Author Guidelines
EMBO Molecular Medicine - Author Guidelines

Reporting Checklist for Life Science Articles (updated January 2022)
This checklist is adapted from Materials Design Analysis Reporting (MDAR) Checklist for Authors. MDAR establishes a minimum set of requirements in transparent reporting in the life sciences (see Statement of Task: 10.31222/osf.io/9sm4x). Please follow the journal's guidelines in preparing your manuscript.
Please note that a copy of this checklist will be published alongside your article.

Abridged guidelines for figures
1. Data
The data shown in figures should satisfy the following conditions:
- the data were obtained and processed according to the field's best practice and are presented to reflect the results of the experiments in an accurate and unbiased manner.
- ideally, figure panels should include only measurements that are directly comparable to each other and obtained with the same assay.
- plots include clearly labeled error bars for independent experiments and sample sizes. Unless justified, error bars should not be shown for technical replicates.
- if n<5, the individual data points from each experiment should be plotted. Any statistical test employed should be justified.
- Source Data should be included to report the data underlying figures according to the guidelines set out in the authorship guidelines on Data Presentation.

2. Captions
Each figure caption should contain the following information, for each panel where they are relevant:
- a specification of the experimental system investigated (eg cell line, species name).
- the assay(s) and method(s) used to carry out the reported observations and measurements.
- an explicit mention of the biological and chemical entity(ies) that are being measured.
- an explicit mention of the biological and chemical entity(ies) that are altered/varied/perturbed in a controlled manner.
- the exact sample size (n) for each experimental group/condition, given as a number, not a range;
- a description of the sample collection allowing the reader to understand whether the samples represent technical or biological replicates (including how many animals, litters, cultures, etc.).
- a statement of how many times the experiment shown was independently replicated in the laboratory.
- definitions of statistical methods and measures:
  - common tests, such as t-test (please specify whether paired vs. unpaired), simple χ2 tests, Wilcoxon and Mann-Whitney tests, can be unambiguously identified by name only, but more complex techniques should be described in the methods section;
  - are tests one-sided or two-sided?
  - are there adjustments for multiple comparisons?
  - exact statistical test results, e.g., P values = x but not P values < x;
  - definition of 'center values' as median or average;
  - definition of error bars as s.d. or s.e.m.

Please complete ALL of the questions below.
Select "Not Applicable" only when the requested information is not relevant for your study.

Materials

Newly Created Materials	Information included in the manuscript?	In which section is the information available? (Reagents and Tools Table, Materials and Methods, Figures, Data Availability Section)
New materials and reagents need to be available; do any restrictions apply?	Not Applicable	

Antibodies	Information included in the manuscript?	In which section is the information available? (Reagents and Tools Table, Materials and Methods, Figures, Data Availability Section)
For **antibodies** provide the following information:<br>- Commercial antibodies: RRID (if possible) or supplier name, catalogue number and or/clone number<br>- Non-commercial: RRID or citation	Yes	Materials and Methods

DNA and RNA sequences	Information included in the manuscript?	In which section is the information available? (Reagents and Tools Table, Materials and Methods, Figures, Data Availability Section)
**Short novel DNA or RNA including primers, probes**: provide the sequences.	Yes	Materials and Methods

Cell materials	Information included in the manuscript?	In which section is the information available? (Reagents and Tools Table, Materials and Methods, Figures, Data Availability Section)
**Cell lines:** Provide species information, strain. Provide accession number in repository **OR** supplier name, catalog number, clone number, and/**OR** RRID.	Yes	Materials and Methods
**Primary cultures:** Provide species, strain, sex of origin, genetic modification status.	Not Applicable	
Report if the cell lines were recently **authenticated** (e.g., by STR profiling) and tested for mycoplasma contamination.	Not Applicable	

Experimental animals	Information included in the manuscript?	In which section is the information available? (Reagents and Tools Table, Materials and Methods, Figures, Data Availability Section)
**Laboratory animals or Model organisms:** Provide species, strain, sex, age, genetic modification status. Provide accession number in repository **OR** supplier name, catalog number, clone number, **OR** RRID.	Not Applicable	
**Animal observed in or captured from the field:** Provide species, sex, and age where possible.	Not Applicable	
Please detail **housing and husbandry conditions**.	Not Applicable	

Plants and microbes	Information included in the manuscript?	In which section is the information available? (Reagents and Tools Table, Materials and Methods, Figures, Data Availability Section)
**Plants:** provide species and strain, ecotype and cultivar where relevant, unique accession number if available, and source (including location for collected wild specimens).	Not Applicable	
**Microbes:** provide species and strain, unique accession number if available, and source.	Not Applicable	

Human research participants	Information included in the manuscript?	In which section is the information available? (Reagents and Tools Table, Materials and Methods, Figures, Data Availability Section)
If collected and within the bounds of privacy constraints report on age, sex and gender or ethnicity for all study participants.	Not Applicable	

Core facilities	Information included in the manuscript?	In which section is the information available? (Reagents and Tools Table, Materials and Methods, Figures, Data Availability Section)
If your work benefited from core facilities, was their service mentioned in the acknowledgments section?	Yes	Acknowledgments

Design

Study protocol	Information included in the manuscript?	In which section is the information available? (Reagents and Tools Table, Materials and Methods, Figures, Data Availability Section)
If study protocol has been **pre-registered, provide DOI in the manuscript**. For clinical trials, provide the trial registration number **OR** cite DOI.	Not Applicable	
Report the **clinical trial registration number** (at ClinicalTrials.gov or equivalent), where applicable.	Not Applicable	

Laboratory protocol	Information included in the manuscript?	In which section is the information available? (Reagents and Tools Table, Materials and Methods, Figures, Data Availability Section)
Provide DOI OR other citation details if **external detailed step-by-step protocols** are available.	Not Applicable	

Experimental study design and statistics	Information included in the manuscript?	In which section is the information available? (Reagents and Tools Table, Materials and Methods, Figures, Data Availability Section)
Include a statement about **sample size** estimate even if no statistical methods were used.	Not Applicable	
Were any steps taken to minimize the effects of subjective bias when allocating animals/samples to treatment (e.g. **randomization procedure**)? If yes, have they been described?	Not Applicable	
Include a statement about **blinding** even if no blinding was done.	Yes	Materials and Methods
Describe **inclusion/exclusion criteria** if samples or animals were excluded from the analysis. Were the criteria pre-established? If sample or data points were omitted from analysis, report if this was due to attrition or intentional exclusion and provide justification.	Not Applicable	
For every figure, are **statistical tests** justified as appropriate? Do the data meet the assumptions of the tests (e.g., normal distribution)? Describe any methods used to assess it. Is there an estimate of variation within each group of data? Is the variance similar between the groups that are being statistically compared?	Yes	Materials and Methods, figure legends

Sample definition and in-laboratory replication	Information included in the manuscript?	In which section is the information available? (Reagents and Tools Table, Materials and Methods, Figures, Data Availability Section)
In the figure legends: state number of times the experiment was **replicated** in laboratory.	Yes	Figure legends
In the figure legends: define whether data describe **technical or biological replicates**.	Yes	Figure legends

Ethics

Ethics	Information included in the manuscript?	In which section is the information available? (Reagents and Tools Table, Materials and Methods, Figures, Data Availability Section)
Studies involving **human participants**: State details of **authority granting ethics approval** (IRB or equivalent committee(s), provide reference number for approval.	Not Applicable	
Studies involving **human participants**: Include a statement confirming that **informed consent** was obtained from all subjects and that the experiments conformed to the principles set out in the WMA Declaration of Helsinki and the Department of Health and Human Services Belmont Report.	Not Applicable	
Studies involving **human participants:** For publication of **patient photos**, include a statement confirming that consent to publish was obtained.	Not Applicable	
Studies involving experimental **animals**: State details of **authority granting ethics approval** (IRB or equivalent committee(s), provide reference number for approval. Include a statement of compliance with ethical regulations.	Not Applicable	
Studies involving **specimen and field samples:** State if relevant **permits** obtained, provide details of authority approving study; if none were required, explain why.	Not Applicable	

Dual Use Research of Concern (DURC)	Information included in the manuscript?	In which section is the information available? (Reagents and Tools Table, Materials and Methods, Figures, Data Availability Section)
Could your study fall under dual use research restrictions? Please check biosecurity documents and list of **select agents and toxins** (CDC): https://www.selectagents.gov/sat/list.htm	Not Applicable	
If you used a select agent, is the security level of the lab appropriate and reported in the manuscript?	Not Applicable	
If a study is subject to dual use research of concern regulations, is the name of the **authority granting approval and reference number** for the regulatory approval provided in the manuscript?	Not Applicable	

Reporting

The MDAR framework recommends adoption of discipline-specific guidelines, established and endorsed through community initiatives. Journals have their own policy about requiring specific guidelines and recommendations to complement MDAR.

Adherence to community standards	Information included in the manuscript?	In which section is the information available? (Reagents and Tools Table, Materials and Methods, Figures, Data Availability Section)
State if relevant guidelines or checklists (e.g., **ICMJE, MIBBI, ARRIVE, PRISMA**) have been followed or provided.	Not Applicable	
For **tumor marker prognostic studies**, we recommend that you follow the **REMARK** reporting guidelines (see link list at top right). See author guidelines, under 'Reporting Guidelines'. Please confirm you have followed these guidelines.	Not Applicable	
For **phase II and III randomized controlled trials**, please refer to the **CONSORT** flow diagram (see link list at top right) and submit the CONSORT checklist (see link list at top right) with your submission. See author guidelines, under 'Reporting Guidelines'. Please confirm you have submitted this list.	Not Applicable	

Data Availability

Data availability	Information included in the manuscript?	In which section is the information available? (Reagents and Tools Table, Materials and Methods, Figures, Data Availability Section)
Have **primary datasets** been deposited according to the journal's guidelines (see 'Data Deposition' section) and the respective accession numbers provided in the Data Availability Section?	Yes	Data Availability Section
Were **human clinical and genomic datasets** deposited in a public access-controlled repository in accordance to ethical obligations to the patients and to the applicable consent agreement?	Not Applicable	
Are **computational models** that are central and integral to a study available without restrictions in a machine-readable form? Were the relevant accession numbers or links provided?	Not Applicable	
If publicly available data were reused, provide the respective **data citations in the reference list**.	Not Applicable	
