# Supplementary material for: Human-specific ARHGAP11B ensures human-like basal progenitor levels in hominid cerebral organoids
Source: EMBO Rep. Author manuscript; Available in PMC 2022 Nov 14. (PMC9646322; doi:10.15252/embr.202254728)
Supplement: Supplementary file 1 [file EMS155265-supplement-Fig_EV1.pdf]

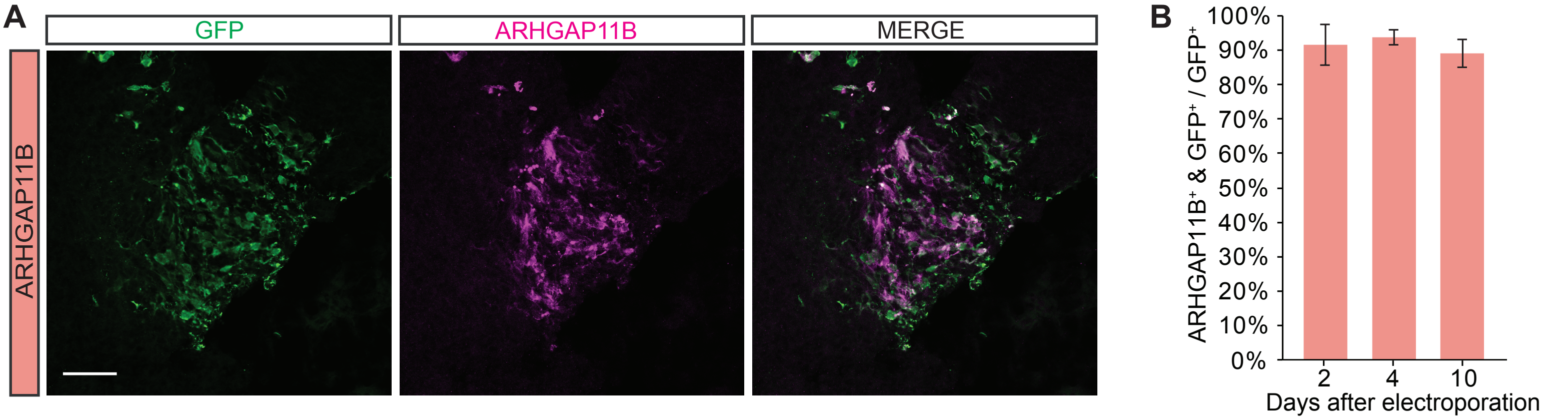
A
GFP
ARHGAP11B
MERGE
ARHGAP11B
B
100%
90%
80%
70%
60%
50%
40%
30%
20%
10%
0%
ARHGAP11B⁺ & GFP⁺ / GFP⁺
2
4
10
Days after electroporation

Figure EV1
