# Supplementary material for: Human-specific ARHGAP11B ensures human-like basal progenitor levels in hominid cerebral organoids
Source: EMBO Rep. Author manuscript; Available in PMC 2022 Nov 14. (PMC9646322; doi:10.15252/embr.202254728)
Supplement: Supplementary file 3 [file EMS155265-supplement-Fig_EV3.pdf]

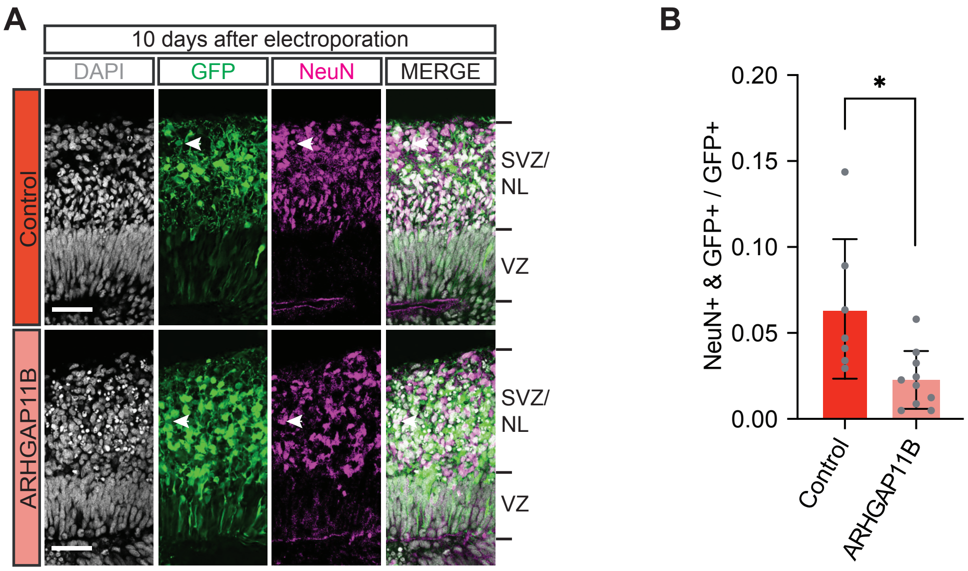
A
10 days after electroporation
DAPI
GFP
NeuN
MERGE
Control
ARHGAP11B
SVZ/
NL
VZ
SVZ/
NL
VZ
B
NeuN+ & GFP+ / GFP+
0.20
0.15
0.10
0.05
0.00
*
Control
ARHGAP11B

Figure EV3
