# Supplementary material for: Human-specific ARHGAP11B ensures human-like basal progenitor levels in hominid cerebral organoids
Source: EMBO Rep. Author manuscript; Available in PMC 2022 Nov 14. (PMC9646322; doi:10.15252/embr.202254728)
Supplement: Supplementary file 4 [file EMS155265-supplement-Fig_EV4.pdf]

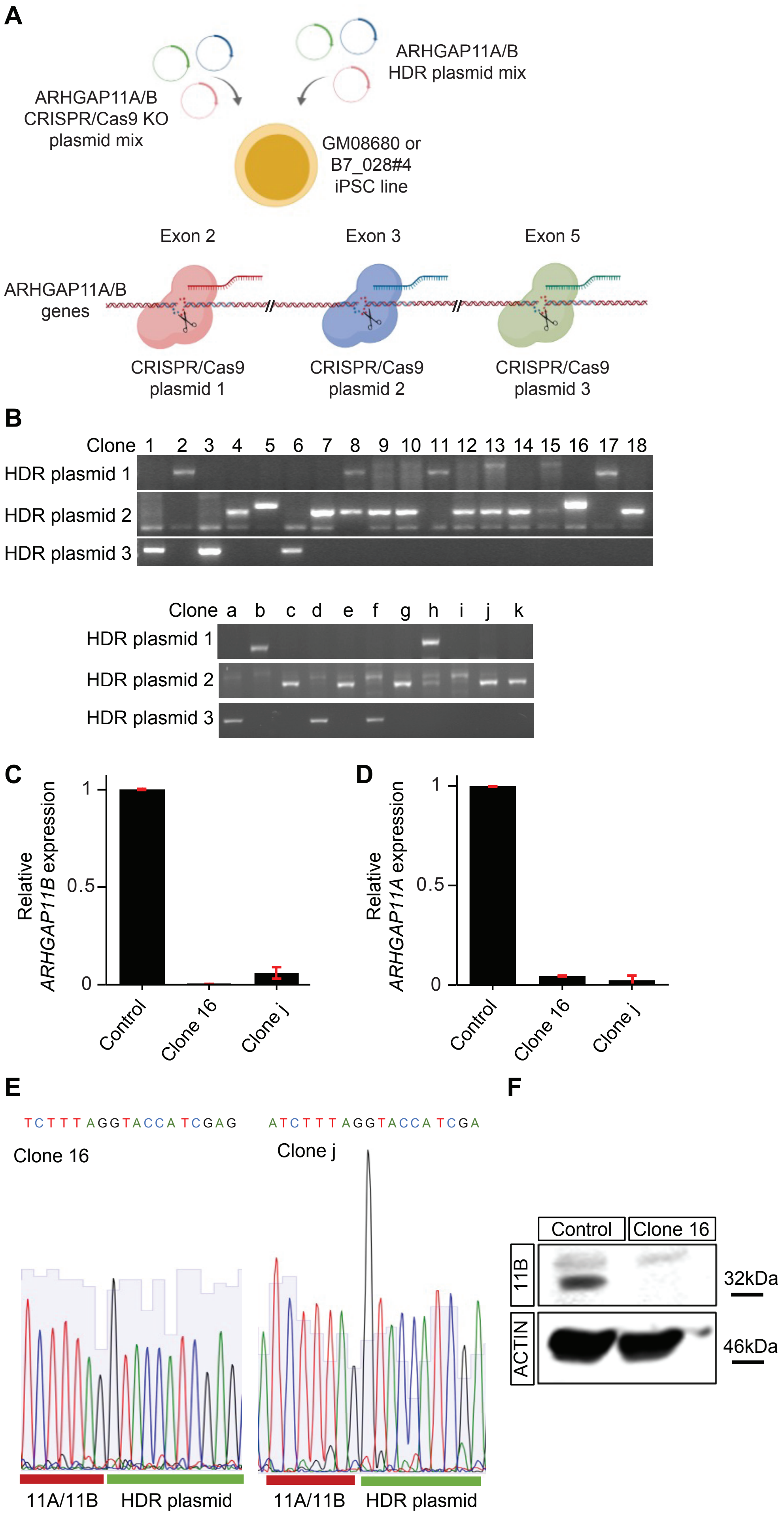
A
ARHGAP11A/B
CRISPR/Cas9 KO
plasmid mix
ARHGAP11A/B
HDR plasmid mix
GM08680 or
B7_028#4
iPSC line
Exon 2
Exon 3
Exon 5
ARHGAP11A/B
genes
CRISPR/Cas9
plasmid 1
CRISPR/Cas9
plasmid 2
CRISPR/Cas9
plasmid 3
B
Clone 1 2 3 4 5 6 7 8 9 10 11 12 13 14 15 16 17 18
HDR plasmid 1
HDR plasmid 2
HDR plasmid 3
Clone a b c d e f g h i j k
HDR plasmid 1
HDR plasmid 2
HDR plasmid 3
C
Relative
ARHGAP11B expression
1
0.5
0
Control
Clone 16
Clone j
D
Relative
ARHGAP11A expression
1
0.5
0
Control
Clone 16
Clone j
E
TCTTTAGGTACCATCGAG
Clone 16
ATCTTTAGGTACCATCGA
Clone j
11A/11B
HDR plasmid
11A/11B
HDR plasmid
F
Control
Clone 16
11B
ACTIN
32kDa
46kDa

Figure EV4
