# Supplementary material for: Human-specific ARHGAP11B ensures human-like basal progenitor levels in hominid cerebral organoids
Source: EMBO Rep. Author manuscript; Available in PMC 2022 Nov 14. (PMC9646322; doi:10.15252/embr.202254728)
Supplement: Supplementary file 8 [file EMS155265-supplement-Supplementary_Material.docx]

**Expanded View Figure legends**

**Figure EV1 - GFP and** ***ARHGAP11B*** **are co-expressed when co-electroporated in chimpanzee cerebral organoids.**

A Double immunofluorescence for GFP (green) and ARHGAP11B (magenta) of a 59 days-old chimpanzee cerebral organoid 4 days after electroporation with GFP expression plasmid plus *ARHGAP11B* expression plasmid. Note that GFP and ARHGAP11B immunofluorescence signals do not completely overlap, as GFP is localized in the cytoplasm whereas ARHGAP11B is localized in mitochondria. Scale bar, 50 µm.

B Quantification of the percentage of GFP+ cells that are ARHGAP11B+ in 57-, 59- and 61-days old chimpanzee cerebral organoids 2, 4 and 10 days after electroporation with GFP expression plasmid plus *ARHGAP11B* expression plasmid. Data are the mean of 5, 6 or 7 ventricle-like structures 2, 4 or 10 days after electroporation of 3 *ARHGAP11B*-transfected cerebral organoids each; error bars indicate SD. 

**Figure EV2 - Expression of** ***ARHGAP11B*** **in chimpanzee cerebral organoids increases the abundance of PCNA-positive cells in the SVZ.**

A Double immunofluorescence for GFP (green) and the cycling cell marker PCNA (yellow), combined with DAPI staining (white), of a 61 days-old chimpanzee cerebral organoids 10 days after electroporation with GFP expression plasmid plus either control plasmid (top) or *ARHGAP11B* expression plasmid (bottom). Tick marks indicate the borders of the VZ and SVZ/NL; arrowheads indicate examples of GFP+ and PCNA+ double-positive cells. Note that the same electroporated regions are depicted in Figure 4 with a different marker (Hu). Scale bar, 50 µm.

B Quantification of the proportion of GFP+ cells in the SVZ/NL that are PCNA+ in 61-days old chimpanzee cerebral organoids 10 days after electroporation with GFP expression plasmid plus either control plasmid (dark red) or *ARHGAP11B* expression plasmid (light red). Data are the mean of 7 control and 10 *ARHGAP11B*-transfected cerebral organoids of two independent batches each; error bars indicate SD; *, P <0.05 (two-sided Student’s *t*-test).

**Figure EV3 - Expression of** ***ARHGAP11B*** **in chimpanzee cerebral organoids decreases the abundance of NeuN-positive cells.**

A Double immunofluorescence for GFP (green) and the neuron marker NeuN (magenta), combined with DAPI staining (white), of a 61 days-old chimpanzee cerebral organoid 10 days after electroporation with GFP expression plasmid plus either control plasmid (top) or *ARHGAP11B* expression plasmid (bottom). Tick marks indicate the borders of the VZ and SVZ/NL; arrowheads indicate examples of GFP+ and NeuN+ double-positive cells. Scale bar, 50 µm.

B Quantification of the proportion of GFP+ cells that are NeuN+ in 61-days old chimpanzee cerebral organoids 10 days after electroporation with GFP expression plasmid plus either control plasmid (dark red) or *ARHGAP11B* expression plasmid (light red). Data are the mean of 7 control and 10 *ARHGAP11B*-transfected cerebral organoids of two independent batches each; error bars indicate SD; *, P <0.05 (two-sided Student’s *t*-test).

**Figure EV4 - Validation of the human** ***ARHGAP11A*** **plus** ***ARHGAP11B*** **double-knockout iPSC clones 16 and j.**

A Schematic overview of the knockout strategy (image created with BioRender.com). A mixture of three CRISPR/Cas9 plasmids, each encoding the gRNA targeting either exon 2, 3 or 5 and SpCas9 ribonuclease, and three Homology-Directed Repair (HDR) plasmids, each containing the corresponding homology arms and encoding a puromycin resistance cassette under the control of the EF-1α promoter, were nucleofected into human GM08680 and B7_028#4 iPSCs. Due to the targeting by the CRISPR/Cas9 plasmids of the sites in exon 2, 3 and 5 of the *ARHGAP11A* and/or *ARHGAP11B* genes, each of these sites should show integration of the corresponding HDR plasmid.

B Genomic PCR validation of the indicated human iPSC clones for integration of the HDR plasmid. PCR primers were designed to recognize the integration of the HDR plasmid in exon 2 (HDR plasmid 1), exon 3 (HDR plasmid 2) or exon 5 (HDR plasmid 3) of *ARHGAP11B*. The image shows 18 of the 24 clones analyzed for the GM08680 iPSC line (clone 1-18) and 11 of the 14 clones analyzed for the B7_028#4 iPSC line (clone a-k). 

C, D Analysis of the relative expression levels of the *ARHGAP11B* (C) and *ARHGAP11A* (D) mRNAs by q-RT-PCR of control, clone 16 and clone j iPSCs, showing virtually complete lack of expression for both genes in clone 16 and clone j, indicative of a homozygous *ARHGAP11A* plus *ARHGAP11B* double-knockout. Data are the mean of 3 independent experiments; error bars indicate SD.

E Sequencing analysis of clone 16 and clone j iPSCs confirmed the disruption of the *ARHGAP11A* plus *ARHGAP11B* genes in exon 3 in both clones due to the integration of the HDR plasmid, indicative of a double-knockout.

F Immunoblot for ARHGAP11B (11B, top) and actin (bottom) of cleared lysates from control (left) and clone 16 (right) iPSCs.
